# Supplementary material for: What Drives the Choice of Local Seasonal Food? Analysis of the Importance of Different Key Motives
Source: Foods. 2021 Nov 6;10(11):2715. doi: 10.3390/foods10112715 (PMC8623070; doi:10.3390/foods10112715)
Supplement: Supplementary file 1 [file foods-10-02715-s001.zip › S1_Factor_Loadings.pdf]

S1: Factor loadings

	Factors							
	1	2	3	4	5	6	7	8
CET1			.725					
CET2			.655					
CET3			.691					
CET4			.536					
GCV1						.713		
GCV2						.697		
GCV4						.662		
LID1		.735						
LID2		.591						
LID3		.743						
LID4		.685						
AUTH1					.660			
AUTH2					.679			
AUTH3					.669			
AUTH4					.586			
PHB1	.751							
HB2	.761							
HB3	.636							
HB4	.702							
HB5	.789							
HB6	.680							
PRI1								.787
PRI2								.762
GAT1							.850	
GAT2							.540	
GAT3							.766	
GID1				.872				
GID2				.624				
GID3				.839				
GID4				.540				

Extraction Method: Principal Axis Factoring.
Rotation Method: Varimax with Kaiser Normalization.
Rotation converged in 6 iterations.
