# Supplementary material for: What Drives the Choice of Local Seasonal Food? Analysis of the Importance of Different Key Motives
Source: Foods. 2021 Nov 6;10(11):2715. doi: 10.3390/foods10112715 (PMC8623070; doi:10.3390/foods10112715)
Supplement: Supplementary file 1 [file foods-10-02715-s001.zip › S2_SEM_Apples_Tomatoes.pdf]

S2: SEM

1 Apples Measurement Model (Summated Scores)

```
                                DATE:  8/19/2021
                                  TIME: 12:57


                          L I S R E L  9.30 (64 Bit)

                                       BY

                         Karl G. Jˆreskog & Dag Sˆrbom



                    This program is published exclusively by
                    Scientific Software International, Inc.
                         http://www.ssicentral.com

        Copyright by Scientific Software International, Inc., 1981-2017
          Use of this program is subject to the terms specified in the
                        Universal Copyright Convention.
```

!LEGEND for the expression used in this file:
PHB-Healthiness Bias summated factor scores, BIAS-Healthiness Bias construct
LocID-Local Identity summated factor scores, LOCALISM-Local Identity construct
CETsum-Consumer Ethnocentrism summated factor scores, ETHNO-Consumer Ethnocentrism construct
GloIDsum-Global Identity summated factor scores, GID-Global Identity construct
AUTH-Authenticity summated factor scores, AUTHENCI-Authenticity construct
GCVsum-Green Consumer Value summated factor scores, GREEN-Green Consumer Values construct
GloAttsum-Global Attitude summated factor scores, GLOBAL-Global Attitude construct
Pricesum-Price Consciousness/Sensibility summated factor scores, PRICESEN-Price Consciousness/Sensibility construct
tn_at-Part worth utility of the local choice, NUTZEN-part worth utility

The following lines were read from file C:\Users\Petra Riefler\Desktop\Apples_Measurement_Model_summatedscores.spl:

```
 !Data
 !ƒpfel
 !Model 2, 16.08.2021 mit factor scores um Multikoll zu beheben
 Observed Variables: PHBsum LocIDsum CETsum GloIDsum AUTHsum GCVsum GloAttsum Pricesum tn_at age gender
edu urban HOUSESIZ food health
 Covariance Matrix:
 0.911
 -0.011 0.756
 0.051 0.086 0.758
 -0.068 -0.025 -0.014 0.791
 0.023 0.128 0.062 0.014 0.807
 0.060 0.006 -0.028 0.038 0.015 0.763
 0.033 -0.003 -0.055 0.034 0.066 0.003 0.745
 -0.043 -0.010 0.010 -0.008 0.024 0.003 0.014 0.746
 4.396 1.291 5.070 1.266 2.644 1.163 -1.519 -4.956 466.543
 0.867 1.933 1.839 -0.585 1.485 2.591 -0.918 -0.975 43.107 243.992
 0.002 -0.004 0.011 0.007 0.021 0.011 0.063 0.008 0.114 0.013 0.251
 -0.034 -0.157 -0.146 0.153 -0.208 0.184 -0.020 -0.069 -1.176 0.198 0.071 1.325
 0.062 0.070 0.011 0.058 -0.033 0.022 0.006 0.070 0.338 -0.311 0.025 -0.032 0.630
 -0.016 -0.061 -0.044 -0.047 -0.104 0.007 0.051 0.133 -1.423 -5.824 -0.001 0.257 0.175 1.182
 0.210 0.153 0.169 0.082 0.103 0.236 0.128 -0.194 5.040 3.992 0.038 0.174 -0.039 -0.188 1.596
 0.223 -0.100 0.093 0.016 0.194 0.535 -0.044 -0.211 7.466 2.917 0.113 0.259 -0.022 -0.049 0.551 2.328
 Sample Size: 251
 Latent Variables: ETHNO GREEN AUTHENTICITY BIAS LOCALISM PRICESENS NUTZEN GLOBAL GID
 Relationships:
 CETsum=1*ETHNO
 set the error variance of CETsum equal to 0.121
 GCVsum = 1*GREEN
 set the error variance of GCVsum equal to 0.163
 AUTHsum = 1*AUTHENTICITY
 set the error variance of AUTHsum equal to 0.098
 LocIDsum = 1*LOCALISM
 set the error variance of LocIDsum equal to 0.158
 Pricesum=1*PRICESENS
```

```
set the error variance of Pricesum equal to 0.208
GloAttsum = 1*GLOBAL
set the error variance of GloAttsum equal to 0.210
GloIDsum=1*GID
set the error variance of GloIDsum equal to 0.178
PHBsum = 1*BIAS
set the error variance of PHBsum equal to 0.105
tn_at=1*NUTZEN
set the error variance of tn_at equal to 0
Options: ND=3 SC RS MI
Path Diagram
End of Problem

Sample Size =   251

!HV Daten aus Studie 1
```

Covariance Matrix

	PHBsum	LocIDsum	CETsum	GloIDsum	AUTHsum	GCVsum
PHBsum	0.911					
LocIDsum	-0.011	0.756				
CETsum	0.051	0.086	0.758			
GloIDsum	-0.068	-0.025	-0.014	0.791		
AUTHsum	0.023	0.128	0.062	0.014	0.807	
GCVsum	0.060	0.006	-0.028	0.038	0.015	0.763
GloAttsu	0.033	-0.003	-0.055	0.034	0.066	0.003
Pricesum	-0.043	-0.010	0.010	-0.008	0.024	0.003
tn_at	4.396	1.291	5.070	1.266	2.644	1.163

Covariance Matrix

	GloAttsu	Pricesum	tn_at

```
GloAttsu      0.745
Pricesum      0.014      0.746
   tn_at     -1.519     -4.956    466.543
```

Total Variance = 472.820 Generalized Variance = 47.642

Largest Eigenvalue = 466.722 Smallest Eigenvalue = 0.588

Condition Number = 28.182

!HV Daten aus Studie 1

Number of Iterations = 0

LISREL Estimates (Maximum Likelihood)

Measurement Equations

```
  PHBsum = 1.000*BIAS, Errorvar.= 0.105, R≤ = 0.885

LocIDsum = 1.000*LOCALISM, Errorvar.= 0.158, R≤ = 0.791

  CETsum = 1.000*ETHNO, Errorvar.= 0.121, R≤ = 0.840

GloIDsum = 1.000*GID, Errorvar.= 0.178, R≤ = 0.775

 AUTHsum = 1.000*AUTHENTI, Errorvar.= 0.0980, R≤ = 0.879

  GCVsum = 1.000*GREEN, Errorvar.= 0.163, R≤ = 0.786

GloAttsu = 1.000*GLOBAL, Errorvar.= 0.210, R≤ = 0.718
```

Pricesum = 1.000*PRICESEN, Errorvar.= 0.208, R≤ = 0.721

tn_at = 1.000*NUTZEN,, R≤ = 1.000

Covariance Matrix of Independent Variables

	ETHNO	GREEN	AUTHENTI	BIAS	LOCALISM	PRICESEN
ETHNO	0.637					
	(0.068)					
	9.414					
GREEN	-0.028	0.600				
	(0.048)	(0.068)				
	-0.583	8.809				
AUTHENTI	0.062	0.015	0.709			
	(0.050)	(0.050)	(0.072)			
	1.252	0.303	9.842			
BIAS	0.051	0.060	0.023	0.806		
	(0.053)	(0.053)	(0.054)	(0.081)		
	0.971	1.137	0.425	9.911		
LOCALISM	0.086	0.006	0.128	-0.011	0.598	
	(0.048)	(0.048)	(0.050)	(0.052)	(0.067)	
	1.788	0.125	2.562	-0.210	8.861	
PRICESEN	0.010	0.003	0.024	-0.043	-0.010	0.538
	(0.047)	(0.048)	(0.049)	(0.052)	(0.047)	(0.067)
	0.211	0.063	0.490	-0.825	-0.211	8.079
NUTZEN	5.070	1.163	2.644	4.396	1.291	-4.956
	(1.229)	(1.193)	(1.236)	(1.331)	(1.188)	(1.218)

	4.124	0.975	2.139	3.304	1.087	-4.068
GLOBAL	-0.055	0.003	0.066	0.033	-0.003	0.014
	(0.048)	(0.048)	(0.049)	(0.052)	(0.047)	(0.047)
	-1.156	0.063	1.344	0.634	-0.063	0.297
GID	-0.014	0.038	0.014	-0.068	-0.025	-0.008
	(0.049)	(0.049)	(0.050)	(0.054)	(0.049)	(0.048)
	-0.286	0.774	0.278	-1.265	-0.512	-0.165

Covariance Matrix of Independent Variables

	NUTZEN	GLOBAL	GID
NUTZEN	466.543		
	(41.646)		
	11.203		
GLOBAL	-1.519	0.535	
	(1.181)	(0.067)	
	-1.287	8.045	
GID	1.266	0.034	0.613
	(1.215)	(0.049)	(0.071)
	1.042	0.701	8.682

Log-likelihood Values

	Estimated Model	Saturated Model
Number of free parameters(t)	45	45
-2ln(L)	3228.790	3228.790
AIC (Akaike, 1974)*	3318.790	3318.790

BIC (Schwarz, 1978)*   3477.436   3477.436

*LISREL uses AIC= 2t - 2ln(L) and BIC = tln(N)- 2ln(L)

Goodness-of-Fit Statistics

Degrees of Freedom for (C1)-(C2)   0
Maximum Likelihood Ratio Chi-Square (C1)   0.0 (P = 1.0000)
Browne's (1984) ADF Chi-Square (C2_NT)   0.0 (P = 1.0000)

The Model is Saturated, the Fit is Perfect !

!HV Daten aus Studie 1

Modification Indices and Expected Change

No Non-Zero Modification Indices for LAMBDA-X

No Non-Zero Modification Indices for PHI

No Non-Zero Modification Indices for THETA-DELTA

!HV Daten aus Studie 1

Standardized Solution

LAMBDA-X

	ETHNO	GREEN	AUTHENTI	BIAS	LOCALISM	PRICESEN
PHBsum	- -	- -	- -	0.898	- -	- -
LocIDsum	- -	- -	- -	- -	0.773	- -
CETsum	0.798	- -	- -	- -	- -	- -

GloIDsum	- -	- -	- -	- -	- -	- -
AUTHsum	- -	- -	0.842	- -	- -	- -
GCVsum	- -	0.775	- -	- -	- -	- -
GloAttsu	- -	- -	- -	- -	- -	- -
Pricesum	- -	- -	- -	- -	- -	0.733
tn_at	- -	- -	- -	- -	- -	- -

LAMBDA-X

	NUTZEN	GLOBAL	GID
PHBsum	- -	- -	- -
LocIDsum	- -	- -	- -
CETsum	- -	- -	- -
GloIDsum	- -	- -	0.783
AUTHsum	- -	- -	- -
GCVsum	- -	- -	- -
GloAttsu	- -	0.731	- -
Pricesum	- -	- -	- -
tn_at	21.600	- -	- -

PHI

	ETHNO	GREEN	AUTHENTI	BIAS	LOCALISM	PRICESEN
ETHNO	1.000					
GREEN	-0.045	1.000				
AUTHENTI	0.092	0.023	1.000			
BIAS	0.071	0.086	0.030	1.000		
LOCALISM	0.139	0.010	0.197	-0.016	1.000	
PRICESEN	0.017	0.005	0.039	-0.065	-0.018	1.000
NUTZEN	0.294	0.070	0.145	0.227	0.077	-0.313
GLOBAL	-0.094	0.005	0.107	0.050	-0.005	0.026
GID	-0.022	0.063	0.021	-0.097	-0.041	-0.014

PHI

	NUTZEN	GLOBAL	GID
NUTZEN	1.000		
GLOBAL	-0.096	1.000	
GID	0.075	0.059	1.000

!HV Daten aus Studie 1

Completely Standardized Solution

LAMBDA-X

	ETHNO	GREEN	AUTHENTI	BIAS	LOCALISM	PRICESEN
PHBsum	- -	- -	- -	0.941	- -	- -
LocIDsum	- -	- -	- -	- -	0.889	- -
CETsum	0.917	- -	- -	- -	- -	- -
GloIDsum	- -	- -	- -	- -	- -	- -
AUTHsum	- -	- -	0.937	- -	- -	- -
GCVsum	- -	0.887	- -	- -	- -	- -
GloAttsu	- -	- -	- -	- -	- -	- -
Pricesum	- -	- -	- -	- -	- -	0.849
tn_at	- -	- -	- -	- -	- -	- -

LAMBDA-X

	NUTZEN	GLOBAL	GID
PHBsum	- -	- -	- -
LocIDsum	- -	- -	- -
CETsum	- -	- -	- -
GloIDsum	- -	- -	0.880
AUTHsum	- -	- -	- -

GCVsum	- -	- -	- -
GloAttsu	- -	0.847	- -
Pricesum	- -	- -	- -
tn_at	1.000	- -	- -

PHI

	ETHNO	GREEN	AUTHENTI	BIAS	LOCALISM	PRICESEN
ETHNO	1.000					
GREEN	-0.045	1.000				
AUTHENTI	0.092	0.023	1.000			
BIAS	0.071	0.086	0.030	1.000		
LOCALISM	0.139	0.010	0.197	-0.016	1.000	
PRICESEN	0.017	0.005	0.039	-0.065	-0.018	1.000
NUTZEN	0.294	0.070	0.145	0.227	0.077	-0.313
GLOBAL	-0.094	0.005	0.107	0.050	-0.005	0.026
GID	-0.022	0.063	0.021	-0.097	-0.041	-0.014

PHI

	NUTZEN	GLOBAL	GID
NUTZEN	1.000		
GLOBAL	-0.096	1.000	
GID	0.075	0.059	1.000

THETA-DELTA

PHBsum	LocIDsum	CETsum	GloIDsum	AUTHsum	GCVsum
0.115	0.209	0.160	0.225	0.121	0.214

THETA-DELTA

```
           GloAttsu   Pricesum      tn_at
           --------   --------   --------
              0.282      0.279       - -

W_A_R_N_I_N_G: THETA-DELTA is not positive definite

                          Time used 0.016 seconds
```

1 Tomatoes Measurement Model (Summated scores)

DATE: 8/19/2021
TIME: 12:51

L I S R E L 9.30 (64 Bit)

BY

Karl G. Jˆreskog & Dag Sˆrbom

This program is published exclusively by
Scientific Software International, Inc.
http://www.ssicentral.com

Copyright by Scientific Software International, Inc., 1981-2017
Use of this program is subject to the terms specified in the
Universal Copyright Convention.

!LEGEND for the expression used in this file:
PHB-Healthiness Bias summated factor scores, BIAS-Healthiness Bias construct
LocID-Local Identity summated factor scores, LOCALISM-Local Identity construct
CETsum-Consumer Ethnocentrism summated factor scores, ETHNO-Consumer Ethnocentrism construct
GloIDsum-Global Identity summated factor scores, GID-Global Identity construct
AUTH-Authenticity summated factor scores, AUTHENCI-Authenticity construct
GCVsum-Green Consumer Value summated factor scores, GREEN-Green Consumer Values construct
GloAttsum-Global Attitude summated factor scores, GLOBAL-Global Attitude construct
Pricesum-Price Consciousness/Sensibility summated factor scores, PRICESEN-Price Consciousness/Sensibility construct
tn_at-Part worth utility of the local choice, NUTZEN-part worth utility
The following lines were read from file C:\Users\Petra Riefler\Desktop\Tomaten_Measurement_Model_summatedscores.spl:

```
!HV Daten aus Studie 1
!Tomaten
!Model 2, 16.08.2021 mit factor scores um Multikoll zu beheben
Observed Variables: PHBsum LocIDsum CETsum GloIDsum AUTHsum GCVsum GloAttsum Pricesum tn_at age gender
edu urban HOUSESIZ food health
Covariance Matrix:
0.800
0.067 0.818
0.054 0.003 0.790
0.071 0.010 0.006 0.955
0.085 0.016 -0.003 -0.035 0.739
0.000 0.028 0.135 0.054 0.051 0.736
-0.034 0.031 0.012 0.015 -0.046 0.020 0.903
0.053 0.063 -0.059 0.026 -0.022 -0.092 0.044 0.812
3.471 2.536 7.973 -0.528 1.691 4.084 -0.356 -7.458 453.020
0.462 0.244 0.617 -2.879 2.337 0.516 -2.909 -1.246 35.130 265.632
0.040 -0.008 0.088 -0.007 -0.004 0.063 0.071 0.040 0.839 -1.257 0.250
-0.134 -0.028 -0.112 0.170 -0.115 0.108 0.052 -0.151 -0.188 -0.959 0.003 1.359
0.019 0.078 -0.033 -0.091 -0.004 -0.029 -0.049 0.041 0.280 -0.482 0.020 -0.117 0.672
0.074 0.070 -0.027 0.169 -0.039 -0.104 0.073 0.058 -1.273 -6.878 0.016 0.063 0.126 1.315
0.123 0.079 0.198 0.131 0.088 0.353 0.090 -0.168 4.828 2.449 0.025 0.082 -0.094 -0.162 1.403
0.160 0.113 0.160 0.039 0.133 0.433 -0.077 -0.139 4.961 3.952 0.120 0.251 -0.013 -0.070 0.570 2.295
Sample Size: 253
Latent Variables: ETHNO GREEN AUTHENTICITY BIAS LOCALISM PRICESENS NUTZEN GLOBAL GID
Relationships:
CETsum=1*ETHNO
set the error variance of CETsum equal to 0.121
GCVsum = 1*GREEN
set the error variance of GCVsum equal to 0.163
AUTHsum = 1*AUTHENTICITY
set the error variance of AUTHsum equal to 0.098
LocIDsum = 1*LOCALISM
set the error variance of LocIDsum equal to 0.158
Pricesum=1*PRICESENS
set the error variance of Pricesum equal to 0.208
```

```
GloAttsum = 1*GLOBAL
set the error variance of GloAttsum equal to 0.210
GloIDsum=1*GID
set the error variance of GloIDsum equal to 0.178
PHBsum = 1*BIAS
set the error variance of PHBsum equal to 0.105
tn_at=1*NUTZEN
set the error variance of tn_at equal to 0
Options: ND=3 SC RS MI
Path Diagram
End of Problem

Sample Size =   253

!HV Daten aus Studie 1

        Covariance Matrix

              PHBsum   LocIDsum     CETsum   GloIDsum    AUTHsum     GCVsum
            --------   --------   --------   --------   --------   --------
  PHBsum       0.800
LocIDsum       0.067      0.818
  CETsum       0.054      0.003      0.790
GloIDsum       0.071      0.010      0.006      0.955
 AUTHsum       0.085      0.016     -0.003     -0.035      0.739
  GCVsum        - -       0.028      0.135      0.054      0.051      0.736
GloAttsu      -0.034      0.031      0.012      0.015     -0.046      0.020
Pricesum       0.053      0.063     -0.059      0.026     -0.022     -0.092
   tn_at       3.471      2.536      7.973     -0.528      1.691      4.084

        Covariance Matrix

            GloAttsu   Pricesum      tn_at
            --------   --------   --------
GloAttsu       0.903
```

```
Pricesum      0.044      0.812
   tn_at     -0.356     -7.458    453.020

Total Variance = 459.573 Generalized Variance = 49.559

Largest Eigenvalue = 453.368 Smallest Eigenvalue = 0.520

Condition Number = 29.520


!HV Daten aus Studie 1

Number of Iterations = 0

LISREL Estimates (Maximum Likelihood)

        Measurement Equations


  PHBsum = 1.000*BIAS, Errorvar.= 0.105, R≤ = 0.869

LocIDsum = 1.000*LOCALISM, Errorvar.= 0.158, R≤ = 0.807

  CETsum = 1.000*ETHNO, Errorvar.= 0.121, R≤ = 0.847

GloIDsum = 1.000*GID, Errorvar.= 0.178, R≤ = 0.814

 AUTHsum = 1.000*AUTHENTI, Errorvar.= 0.0980, R≤ = 0.867

  GCVsum = 1.000*GREEN, Errorvar.= 0.163, R≤ = 0.779

GloAttsu = 1.000*GLOBAL, Errorvar.= 0.210, R≤ = 0.767

Pricesum = 1.000*PRICESEN, Errorvar.= 0.208, R≤ = 0.744
```

tn_at = 1.000*NUTZEN,, R≤ = 1.000

Covariance Matrix of Independent Variables

	ETHNO	GREEN	AUTHENTI	BIAS	LOCALISM	PRICESEN
ETHNO	0.669					
	(0.070)					
	9.525					
GREEN	0.135	0.573				
	(0.049)	(0.065)				
	2.773	8.756				
AUTHENTI	-0.003	0.051	0.641			
	(0.048)	(0.046)	(0.066)			
	-0.062	1.097	9.756			
BIAS	0.054	- -	0.085	0.695		
	(0.050)	(0.048)	(0.049)	(0.071)		
	1.078	0.000	1.748	9.771		
LOCALISM	0.003	0.028	0.016	0.067	0.660	
	(0.051)	(0.049)	(0.049)	(0.051)	(0.073)	
	0.059	0.574	0.327	1.313	9.075	
PRICESEN	-0.059	-0.092	-0.022	0.053	0.063	0.604
	(0.050)	(0.049)	(0.049)	(0.051)	(0.051)	(0.072)
	-1.169	-1.880	-0.452	1.044	1.226	8.366
NUTZEN	7.973	4.084	1.691	3.471	2.536	-7.458
	(1.291)	(1.176)	(1.155)	(1.217)	(1.221)	(1.294)
	6.177	3.472	1.464	2.853	2.077	-5.765

GLOBAL	0.012	0.020	-0.046	-0.034	0.031	0.044
	(0.053)	(0.051)	(0.051)	(0.053)	(0.054)	(0.054)
	0.226	0.390	-0.894	-0.636	0.573	0.816
GID	0.006	0.054	-0.035	0.071	0.010	0.026
	(0.055)	(0.053)	(0.053)	(0.055)	(0.056)	(0.055)
	0.110	1.022	-0.662	1.288	0.180	0.469

Covariance Matrix of Independent Variables

	NUTZEN	GLOBAL	GID
NUTZEN	453.020		
	(40.278)		
	11.247		
GLOBAL	-0.356	0.693	
	(1.272)	(0.080)	
	-0.280	8.632	
GID	-0.528	0.015	0.777
	(1.308)	(0.058)	(0.085)
	-0.404	0.257	9.151

Log-likelihood Values

	Estimated Model	Saturated Model
Number of free parameters(t)	45	45
-2ln(L)	3264.502	3264.502
AIC (Akaike, 1974)*	3354.502	3354.502
BIC (Schwarz, 1978)*	3513.505	3513.505

*LISREL uses AIC= 2t - 2ln(L) and BIC = tln(N)- 2ln(L)

Goodness-of-Fit Statistics

Degrees of Freedom for (C1)-(C2)	0
Maximum Likelihood Ratio Chi-Square (C1)	0.0 (P = 1.0000)
Browne's (1984) ADF Chi-Square (C2_NT)	0.0 (P = 1.0000)

The Model is Saturated, the Fit is Perfect !

!HV Daten aus Studie 1

Modification Indices and Expected Change

No Non-Zero Modification Indices for LAMBDA-X

No Non-Zero Modification Indices for PHI

No Non-Zero Modification Indices for THETA-DELTA

!HV Daten aus Studie 1

Standardized Solution

LAMBDA-X

	ETHNO	GREEN	AUTHENTI	BIAS	LOCALISM	PRICESEN
PHBsum	- -	- -	- -	0.834	- -	- -
LocIDsum	- -	- -	- -	- -	0.812	- -
CETsum	0.818	- -	- -	- -	- -	- -
GloIDsum	- -	- -	- -	- -	- -	- -

AUTHsum	- -	- -	0.801	- -	- -	- -
GCVsum	- -	0.757	- -	- -	- -	- -
GloAttsu	- -	- -	- -	- -	- -	- -
Pricesum	- -	- -	- -	- -	- -	0.777
tn_at	- -	- -	- -	- -	- -	- -

LAMBDA-X

	NUTZEN	GLOBAL	GID
PHBsum	- -	- -	- -
LocIDsum	- -	- -	- -
CETsum	- -	- -	- -
GloIDsum	- -	- -	0.881
AUTHsum	- -	- -	- -
GCVsum	- -	- -	- -
GloAttsu	- -	0.832	- -
Pricesum	- -	- -	- -
tn_at	21.284	- -	- -

PHI

	ETHNO	GREEN	AUTHENTI	BIAS	LOCALISM	PRICESEN
ETHNO	1.000					
GREEN	0.218	1.000				
AUTHENTI	-0.005	0.084	1.000			
BIAS	0.079	- -	0.127	1.000		
LOCALISM	0.005	0.046	0.025	0.099	1.000	
PRICESEN	-0.093	-0.156	-0.035	0.082	0.100	1.000
NUTZEN	0.458	0.253	0.099	0.196	0.147	-0.451
GLOBAL	0.018	0.032	-0.069	-0.049	0.046	0.068
GID	0.008	0.081	-0.050	0.097	0.014	0.038

PHI

	NUTZEN	GLOBAL	GID
NUTZEN	1.000		
GLOBAL	-0.020	1.000	
GID	-0.028	0.020	1.000

!HV Daten aus Studie 1

Completely Standardized Solution

LAMBDA-X

	ETHNO	GREEN	AUTHENTI	BIAS	LOCALISM	PRICESEN
PHBsum	- -	- -	- -	0.932	- -	- -
LocIDsum	- -	- -	- -	- -	0.898	- -
CETsum	0.920	- -	- -	- -	- -	- -
GloIDsum	- -	- -	- -	- -	- -	- -
AUTHsum	- -	- -	0.931	- -	- -	- -
GCVsum	- -	0.882	- -	- -	- -	- -
GloAttsu	- -	- -	- -	- -	- -	- -
Pricesum	- -	- -	- -	- -	- -	0.862
tn_at	- -	- -	- -	- -	- -	- -

LAMBDA-X

	NUTZEN	GLOBAL	GID
PHBsum	- -	- -	- -
LocIDsum	- -	- -	- -
CETsum	- -	- -	- -
GloIDsum	- -	- -	0.902
AUTHsum	- -	- -	- -
GCVsum	- -	- -	- -

GloAttsu	- -	0.876	- -
Pricesum	- -	- -	- -
tn_at	1.000	- -	- -

PHI

	ETHNO	GREEN	AUTHENTI	BIAS	LOCALISM	PRICESEN
ETHNO	1.000					
GREEN	0.218	1.000				
AUTHENTI	-0.005	0.084	1.000			
BIAS	0.079	- -	0.127	1.000		
LOCALISM	0.005	0.046	0.025	0.099	1.000	
PRICESEN	-0.093	-0.156	-0.035	0.082	0.100	1.000
NUTZEN	0.458	0.253	0.099	0.196	0.147	-0.451
GLOBAL	0.018	0.032	-0.069	-0.049	0.046	0.068
GID	0.008	0.081	-0.050	0.097	0.014	0.038

PHI

	NUTZEN	GLOBAL	GID
NUTZEN	1.000		
GLOBAL	-0.020	1.000	
GID	-0.028	0.020	1.000

THETA-DELTA

PHBsum	LocIDsum	CETsum	GloIDsum	AUTHsum	GCVsum
0.131	0.193	0.153	0.186	0.133	0.221

THETA-DELTA

GloAttsu	Pricesum	tn_at

```
           --------  --------  --------
              0.233     0.256      - -

W_A_R_N_I_N_G: THETA-DELTA is not positive definite

                          Time used 0.016 seconds
```

2 Apples Multiitem for Evaluation of Reliability and Validity incl Fit Statistics

DATE: 9/15/2021
TIME: 8:56

L I S R E L 9.30 (64 Bit)

BY

Karl G. Jˆreskog & Dag Sˆrbom

This program is published exclusively by
Scientific Software International, Inc.
http://www.ssicentral.com

Copyright by Scientific Software International, Inc., 1981-2017
Use of this program is subject to the terms specified in the
Universal Copyright Convention.

!LEGEND for the expression used in this file:
cet-Consumerethnocentrism item, ETHNO-Consumerethnocentrism construct
env-Green Consumer Value item, GREEN-Green Consumer Value construct
aut-Authenticity item, AUTHENTI-Authenticity construct
qual-Healthiness Bias item, QUALITY-Healthiness Bias construct
localID-Local Identity item, LOCALISM-Local Identity construct
tn_at-Part worth utility of the local choice
price-Price Consciousness item, PRICESENS-Price Consciousness/Sensibility construct
globatt-Global Attitude item, GLOBAL-Global Attitude construct
globID-Global Identity item, GID-Global Identity construct

The following lines were read from file C:\Users\Petra Riefler\Desktop\Apples_Measurement_Model_Multiitem.spl:

```
 !Data
 !Apples
 !Update 15.9.
 Observed Variables: cet1 cet2 cet3 cet4 env1 env2 env3 env4 env5 aut1 aut2 aut3 aut4 qual1 qual2 qual3
qual4 qual5 qual6 localID1 localID2 localID3 localID4 tn_at price1 price2 globatt1 globatt2 globatt3
globID1 globID2 globID3 globID4
 Covariance Matrix:
 2.151
 1.519 2.341
 1.461 1.334 2.116
 1.206 1.113 1.349 1.892
 0.548 0.568 0.734 0.643 1.434
 0.587 0.671 0.739 0.700 1.118 1.818
 -0.026 0.030 0.227 0.349 0.336 0.496 2.863
 0.487 0.607 0.569 0.513 0.960 1.097 0.507 1.788
 0.289 0.472 0.468 0.607 0.635 0.842 1.013 0.686 2.830
 0.791 0.915 0.804 0.761 0.666 0.537 0.154 0.616 0.093 1.666
 0.836 0.876 0.895 0.797 0.665 0.538 0.243 0.545 0.176 1.082 1.423
 0.864 0.865 0.830 0.758 0.603 0.475 0.050 0.427 0.094 1.132 1.136 1.488
 0.976 0.905 0.882 0.846 0.619 0.567 0.151 0.471 0.074 1.124 1.160 1.174 1.672
 0.891 0.926 0.898 0.845 0.584 0.578 0.258 0.525 0.232 0.759 0.776 0.745 0.866 1.782
 0.846 0.865 1.038 0.863 0.682 0.669 0.442 0.547 0.370 0.844 0.846 0.850 0.906 1.391 1.699
 0.653 0.573 0.700 0.888 0.527 0.430 0.362 0.450 0.221 0.692 0.631 0.628 0.760 1.079 1.065 1.829
 0.907 0.843 1.057 0.861 0.670 0.565 0.211 0.430 0.208 0.734 0.824 0.796 0.836 1.278 1.283 0.968 1.680
 0.626 0.585 0.700 0.768 0.561 0.497 0.327 0.501 0.199 0.695 0.659 0.637 0.733 1.030 1.058 1.095 1.020
1.439
 0.735 0.577 0.664 0.681 0.499 0.454 0.206 0.442 -0.034 0.523 0.600 0.578 0.647 1.019 1.009 0.902 0.883
1.040 1.693
 0.737 0.781 0.845 0.887 0.553 0.487 -0.087 0.363 -0.037 0.753 0.747 0.855 0.940 0.656 0.616 0.477 0.557
0.470 0.479 2.235
 0.985 0.904 0.916 0.842 0.543 0.404 -0.128 0.493 -0.037 0.899 0.888 0.946 1.102 0.822 0.667 0.532 0.689
0.492 0.366 1.386 2.262
 0.727 0.770 0.740 0.815 0.491 0.381 0.076 0.365 0.001 0.726 0.782 0.785 0.861 0.652 0.570 0.531 0.601
0.537 0.513 1.228 1.091 1.506
```

0.621 0.670 0.639 0.701 0.480 0.353 0.175 0.350 0.282 0.726 0.777 0.743 0.824 0.557 0.440 0.437 0.545
0.526 0.352 0.971 1.044 0.929 1.423
9.565 10.504 8.779 11.300 3.062 7.005 7.111 4.420 9.438 6.528 5.934 6.003 6.670 5.991 8.016 5.675 7.322
6.564 4.899 2.800 3.311 3.732 5.863 466.543
-0.267 -0.160 -0.177 -0.580 -0.206 -0.230 -0.599 -0.053 -0.775 0.015 -0.083 0.032 -0.028 -0.159 -0.005
-0.157 -0.168 -0.237 -0.243 0.171 0.189 0.024 -0.126 -6.731 2.312
-0.195 -0.235 -0.091 -0.417 -0.192 -0.428 -0.562 -0.198 -1.105 0.023 -0.099 -0.058 -0.099 -0.026 -0.036
-0.045 -0.030 -0.130 -0.077 0.276 0.188 -0.056 -0.162 -10.638 1.599 2.638
-0.169 -0.208 -0.133 0.054 0.143 0.047 -0.061 -0.003 -0.357 0.065 0.213 0.081 0.161 -0.004 0.004 0.302
-0.041 0.067 0.036 0.161 0.109 0.102 -0.029 -3.152 0.152 0.209 1.782
-0.138 -0.031 -0.090 -0.062 -0.017 -0.065 -0.457 -0.050 -0.580 0.263 0.110 0.125 0.085 0.111 0.085
-0.122 0.083 -0.049 0.016 0.196 0.308 -0.150 -0.126 -4.516 0.491 0.762 0.858 2.507
-0.104 -0.122 -0.071 0.120 0.120 0.102 -0.038 0.227 -0.120 0.386 0.274 0.173 0.273 0.061 0.151 0.173
-0.014 0.140 0.097 0.077 0.164 0.073 0.108 0.090 0.189 0.215 1.068 0.869 1.690
0.243 0.356 0.500 0.410 0.723 0.811 0.433 0.628 1.154 0.439 0.314 0.318 0.422 0.278 0.522 0.280 0.379
0.297 0.270 0.031 0.039 0.003 0.120 7.466 -0.364 -0.502 -0.114 -0.231 0.086 2.328
0.037 -0.011 -0.001 0.030 -0.006 0.028 0.171 0.051 0.052 0.029 0.042 0.012 0.040 0.009 0.014 0.045 0.037
-0.012 -0.022 0.008 -0.044 0.015 0.018 0.114 0.033 0.018 0.097 0.053 0.077 0.113 0.251
0.203 0.232 0.298 0.235 0.323 0.256 0.023 0.166 0.280 0.273 0.201 0.271 0.151 0.095 0.276 0.143 0.108
0.090 0.091 0.189 0.217 0.185 0.245 2.490 0.030 -0.184 -0.056 -0.152 -0.027 0.176 0.025 1.077
-0.313 -0.346 -0.108 -0.100 0.178 0.180 0.312 0.045 0.133 -0.192 -0.286 -0.274 -0.298 -0.139 -0.097
-0.027 -0.116 -0.120 -0.025 -0.347 -0.301 -0.122 -0.216 -1.176 -0.040 -0.168 -0.011 -0.125 -0.003 0.259
0.071 0.041 1.325
0.012 -0.020 0.120 0.058 0.020 0.052 0.031 0.116 0.014 0.043 0.005 0.025 0.053 0.071 0.035 0.135 0.124
0.103 0.045 0.115 0.114 0.061 0.132 0.338 0.135 0.109 0.001 0.048 0.040 -0.022 0.025 -0.010 -0.032 0.630
-0.163 -0.160 -0.109 -0.060 0.167 0.319 0.304 0.448 0.239 -0.043 -0.034 -0.067 -0.227 -0.056 -0.074
-0.203 -0.072 -0.146 -0.142 -0.099 -0.199 -0.067 -0.002 -0.115 0.086 0.017 0.355 0.454 0.322 0.035 0.022
-0.112 0.204 0.123 2.257
0.089 -0.118 0.110 0.192 0.351 0.392 0.339 0.490 0.099 0.267 0.238 0.117 0.145 0.064 0.131 0.186 0.127
0.228 0.207 0.132 0.053 0.230 0.356 1.720 0.107 0.167 0.236 0.393 0.486 0.102 0.033 -0.016 0.172 0.051
1.066 1.707
-0.060 -0.223 -0.062 0.045 0.134 0.362 0.315 0.382 0.048 -0.002 -0.023 -0.009 -0.142 -0.019 -0.075
-0.233 -0.048 -0.103 -0.031 -0.222 -0.073 -0.046 -0.005 3.165 -0.049 -0.114 0.083 0.374 0.233 -0.038
0.005 -0.024 0.300 0.069 1.643 1.072 2.337
0.117 0.120 0.165 0.308 0.502 0.591 0.455 0.491 0.214 0.351 0.372 0.292 0.312 0.206 0.149 0.194 0.177

```
0.166 0.091 0.186 0.308 0.284 0.395 2.121 -0.208 -0.125 0.280 0.097 0.209 0.299 -0.009 0.078 0.197 -0.020
0.768 0.656 0.754 1.337
 Sample Size: 251
 Latent Variables: ETHNO GREEN AUTHENTICITY QUALITY LOCALISM PRICESENS NUTZEN GLOBAL GID
 Relationships:
 cet1=1*ETHNO
 cet2 cet3 cet4 = ETHNO
 env1 = 1*GREEN
 env2 env4 = GREEN
 aut1 = 1*AUTHENTICITY
 aut2 aut3 aut4 = AUTHENTICITY
 qual1 = 1*QUALITY
 qual2 qual3 qual4 qual5 qual6 = QUALITY
 tn_at=1*NUTZEN
 set the error variance of tn_at equal to 0
 price1=1*PRICESENS
 price2 = PRICESENS
 localID1 = 1*LOCALISM
 localID2 localID3 localID4 = LOCALISM
 globatt1 = 1*GLOBAL
 globatt2 globatt3 =GLOBAL
 globID1=1*GID
 globID2 globID3 globID4 =GID
 Options: ND=3 SC RS MI
 Path Diagram
 End of Problem

 Sample Size =   251

 !HV Daten aus Studie 1

         Covariance Matrix

                cet1       cet2       cet3       cet4       env1       env2
            --------   --------   --------   --------   --------   --------
```

cet1	2.151					
cet2	1.519	2.341				
cet3	1.461	1.334	2.116			
cet4	1.206	1.113	1.349	1.892		
env1	0.548	0.568	0.734	0.643	1.434	
env2	0.587	0.671	0.739	0.700	1.118	1.818
env4	0.487	0.607	0.569	0.513	0.960	1.097
aut1	0.791	0.915	0.804	0.761	0.666	0.537
aut2	0.836	0.876	0.895	0.797	0.665	0.538
aut3	0.864	0.865	0.830	0.758	0.603	0.475
aut4	0.976	0.905	0.882	0.846	0.619	0.567
qual1	0.891	0.926	0.898	0.845	0.584	0.578
qual2	0.846	0.865	1.038	0.863	0.682	0.669
qual3	0.653	0.573	0.700	0.888	0.527	0.430
qual4	0.907	0.843	1.057	0.861	0.670	0.565
qual5	0.626	0.585	0.700	0.768	0.561	0.497
qual6	0.735	0.577	0.664	0.681	0.499	0.454
localID1	0.737	0.781	0.845	0.887	0.553	0.487
localID2	0.985	0.904	0.916	0.842	0.543	0.404
localID3	0.727	0.770	0.740	0.815	0.491	0.381
localID4	0.621	0.670	0.639	0.701	0.480	0.353
tn_at	9.565	10.504	8.779	11.300	3.062	7.005
price1	-0.267	-0.160	-0.177	-0.580	-0.206	-0.230
price2	-0.195	-0.235	-0.091	-0.417	-0.192	-0.428
globatt1	-0.169	-0.208	-0.133	0.054	0.143	0.047
globatt2	-0.138	-0.031	-0.090	-0.062	-0.017	-0.065
globatt3	-0.104	-0.122	-0.071	0.120	0.120	0.102
globID1	-0.163	-0.160	-0.109	-0.060	0.167	0.319
globID2	0.089	-0.118	0.110	0.192	0.351	0.392
globID3	-0.060	-0.223	-0.062	0.045	0.134	0.362
globID4	0.117	0.120	0.165	0.308	0.502	0.591

Covariance Matrix

	env4	aut1	aut2	aut3	aut4	qual1

env4	1.788					
aut1	0.616	1.666				
aut2	0.545	1.082	1.423			
aut3	0.427	1.132	1.136	1.488		
aut4	0.471	1.124	1.160	1.174	1.672	
qual1	0.525	0.759	0.776	0.745	0.866	1.782
qual2	0.547	0.844	0.846	0.850	0.906	1.391
qual3	0.450	0.692	0.631	0.628	0.760	1.079
qual4	0.430	0.734	0.824	0.796	0.836	1.278
qual5	0.501	0.695	0.659	0.637	0.733	1.030
qual6	0.442	0.523	0.600	0.578	0.647	1.019
localID1	0.363	0.753	0.747	0.855	0.940	0.656
localID2	0.493	0.899	0.888	0.946	1.102	0.822
localID3	0.365	0.726	0.782	0.785	0.861	0.652
localID4	0.350	0.726	0.777	0.743	0.824	0.557
tn_at	4.420	6.528	5.934	6.003	6.670	5.991
price1	-0.053	0.015	-0.083	0.032	-0.028	-0.159
price2	-0.198	0.023	-0.099	-0.058	-0.099	-0.026
globatt1	-0.003	0.065	0.213	0.081	0.161	-0.004
globatt2	-0.050	0.263	0.110	0.125	0.085	0.111
globatt3	0.227	0.386	0.274	0.173	0.273	0.061
globID1	0.448	-0.043	-0.034	-0.067	-0.227	-0.056
globID2	0.490	0.267	0.238	0.117	0.145	0.064
globID3	0.382	-0.002	-0.023	-0.009	-0.142	-0.019
globID4	0.491	0.351	0.372	0.292	0.312	0.206

Covariance Matrix

	qual2	qual3	qual4	qual5	qual6	localID1
qual2	1.699					
qual3	1.065	1.829				
qual4	1.283	0.968	1.680			
qual5	1.058	1.095	1.020	1.439		

qual6	1.009	0.902	0.883	1.040	1.693	
localID1	0.616	0.477	0.557	0.470	0.479	2.235
localID2	0.667	0.532	0.689	0.492	0.366	1.386
localID3	0.570	0.531	0.601	0.537	0.513	1.228
localID4	0.440	0.437	0.545	0.526	0.352	0.971
tn_at	8.016	5.675	7.322	6.564	4.899	2.800
price1	-0.005	-0.157	-0.168	-0.237	-0.243	0.171
price2	-0.036	-0.045	-0.030	-0.130	-0.077	0.276
globatt1	0.004	0.302	-0.041	0.067	0.036	0.161
globatt2	0.085	-0.122	0.083	-0.049	0.016	0.196
globatt3	0.151	0.173	-0.014	0.140	0.097	0.077
globID1	-0.074	-0.203	-0.072	-0.146	-0.142	-0.099
globID2	0.131	0.186	0.127	0.228	0.207	0.132
globID3	-0.075	-0.233	-0.048	-0.103	-0.031	-0.222
globID4	0.149	0.194	0.177	0.166	0.091	0.186

Covariance Matrix

	localID2	localID3	localID4	tn_at	price1	price2
localID2	2.262					
localID3	1.091	1.506				
localID4	1.044	0.929	1.423			
tn_at	3.311	3.732	5.863	466.543		
price1	0.189	0.024	-0.126	-6.731	2.312	
price2	0.188	-0.056	-0.162	-10.638	1.599	2.638
globatt1	0.109	0.102	-0.029	-3.152	0.152	0.209
globatt2	0.308	-0.150	-0.126	-4.516	0.491	0.762
globatt3	0.164	0.073	0.108	0.090	0.189	0.215
globID1	-0.199	-0.067	-0.002	-0.115	0.086	0.017
globID2	0.053	0.230	0.356	1.720	0.107	0.167
globID3	-0.073	-0.046	-0.005	3.165	-0.049	-0.114
globID4	0.308	0.284	0.395	2.121	-0.208	-0.125

Covariance Matrix

	globatt1	globatt2	globatt3	globID1	globID2	globID3
globatt1	1.782					
globatt2	0.858	2.507				
globatt3	1.068	0.869	1.690			
globID1	0.355	0.454	0.322	2.257		
globID2	0.236	0.393	0.486	1.066	1.707	
globID3	0.083	0.374	0.233	1.643	1.072	2.337
globID4	0.280	0.097	0.209	0.768	0.656	0.754

Covariance Matrix

	globID4
globID4	1.337

Total Variance = 522.447 Generalized Variance = 75.669

Largest Eigenvalue = 469.116 Smallest Eigenvalue = 0.234

Condition Number = 44.773

WARNING: The Condition Number indicates severe multicollinearity.

One or more variables may be redundant.

!HV Daten aus Studie 1

Number of Iterations = 17

LISREL Estimates (Maximum Likelihood)

Measurement Equations

```
    cet1 = 1.000*ETHNO, Errorvar.= 0.725  , R≤ = 0.663
Standerr                          (0.0845)
Z-values                           8.580
P-values                           0.000

    cet2 = 0.964*ETHNO, Errorvar.= 1.016 , R≤ = 0.566
Standerr  (0.0748)                (0.107)
Z-values   12.880                  9.473
P-values   0.000                   0.000

    cet3 = 1.014*ETHNO, Errorvar.= 0.649  , R≤ = 0.693
Standerr  (0.0692)                (0.0793)
Z-values   14.664                  8.189
P-values   0.000                   0.000

    cet4 = 0.893*ETHNO, Errorvar.= 0.754  , R≤ = 0.601
Standerr  (0.0667)                (0.0820)
Z-values   13.392                  9.198
P-values   0.000                   0.000

    env1 = 1.000*GREEN, Errorvar.= 0.420  , R≤ = 0.707
Standerr                          (0.0642)
Z-values                           6.549
P-values                           0.000

    env2 = 1.103*GREEN, Errorvar.= 0.586  , R≤ = 0.678
Standerr  (0.0819)                (0.0826)
Z-values   13.470                  7.091
P-values   0.000                   0.000

    env4 = 0.964*GREEN, Errorvar.= 0.847  , R≤ = 0.526
Standerr  (0.0809)                (0.0929)
```

```
Z-values   11.906                  9.118
P-values   0.000                   0.000

    aut1 = 1.000*AUTHENTI, Errorvar.= 0.597  , R≤ = 0.642
Standerr                            (0.0619)
Z-values                             9.651
P-values                             0.000

    aut2 = 1.019*AUTHENTI, Errorvar.= 0.312  , R≤ = 0.781
Standerr  (0.0625)                  (0.0385)
Z-values   16.306                    8.094
P-values   0.000                     0.000

    aut3 = 1.031*AUTHENTI, Errorvar.= 0.351  , R≤ = 0.764
Standerr  (0.0642)                  (0.0419)
Z-values   16.064                    8.377
P-values   0.000                     0.000

    aut4 = 1.073*AUTHENTI, Errorvar.= 0.442  , R≤ = 0.735
Standerr  (0.0685)                  (0.0504)
Z-values   15.646                    8.780
P-values   0.000                     0.000

   qual1 = 1.000*QUALITY, Errorvar.= 0.451  , R≤ = 0.747
Standerr                           (0.0514)
Z-values                            8.769
P-values                            0.000

   qual2 = 1.013*QUALITY, Errorvar.= 0.332  , R≤ = 0.805
Standerr  (0.0522)                 (0.0425)
Z-values   19.404                   7.820
P-values   0.000                    0.000

   qual3 = 0.825*QUALITY, Errorvar.= 0.923  , R≤ = 0.496
Standerr  (0.0635)                 (0.0888)
```

```
Z-values   12.989                   10.393
P-values   0.000                    0.000

   qual4 = 0.942*QUALITY, Errorvar.= 0.499  , R≤ = 0.703
Standerr  (0.0549)                 (0.0539)
Z-values   17.166                   9.256
P-values   0.000                    0.000

   qual5 = 0.822*QUALITY, Errorvar.= 0.540  , R≤ = 0.625
Standerr  (0.0529)                 (0.0549)
Z-values   15.526                   9.836
P-values   0.000                    0.000

   qual6 = 0.774*QUALITY, Errorvar.= 0.896  , R≤ = 0.471
Standerr  (0.0618)                 (0.0856)
Z-values   12.521                   10.467
P-values   0.000                    0.000

localID1 = 1.000*LOCALISM, Errorvar.= 0.902 , R≤ = 0.596
Standerr                            (0.101)
Z-values                             8.943
P-values                             0.000

localID2 = 1.002*LOCALISM, Errorvar.= 0.924 , R≤ = 0.591
Standerr  (0.0816)                  (0.103)
Z-values   12.272                    8.989
P-values   0.000                     0.000

localID3 = 0.875*LOCALISM, Errorvar.= 0.486  , R≤ = 0.677
Standerr  (0.0663)                  (0.0608)
Z-values   13.192                    7.987
P-values   0.000                     0.000

localID4 = 0.778*LOCALISM, Errorvar.= 0.617  , R≤ = 0.566
Standerr  (0.0649)                  (0.0670)
```

```
Z-values   11.980                    9.209
P-values   0.000                     0.000

   tn_at = 1.000*NUTZEN,, R≤ = 1.000

  price1 = 1.000*PRICESEN, Errorvar.= 1.281 , R≤ = 0.446
Standerr                           (0.214)
Z-values                            5.998
P-values                            0.000

  price2 = 1.551*PRICESEN, Errorvar.= 0.158 , R≤ = 0.940
Standerr  (0.292)                  (0.434)
Z-values   5.309                    0.364
P-values   0.000                    0.716

globatt1 = 1.000*GLOBAL, Errorvar.= 0.788 , R≤ = 0.558
Standerr                         (0.123)
Z-values                          6.429
P-values                          0.000

globatt2 = 0.869*GLOBAL, Errorvar.= 1.757 , R≤ = 0.299
Standerr  (0.117)                (0.179)
Z-values   7.401                  9.837
P-values   0.000                  0.000

globatt3 = 1.056*GLOBAL, Errorvar.= 0.581 , R≤ = 0.656
Standerr  (0.124)                (0.122)
Z-values   8.530                  4.746
P-values   0.000                  0.000

 globID1 = 1.000*GID, Errorvar.= 0.646 , R≤ = 0.714
Standerr                      (0.107)
Z-values                       6.015
P-values                       0.000
```

```
 globID2 = 0.688*GID, Errorvar.= 0.944  , R≤ = 0.447
Standerr  (0.0646)             (0.0984)
Z-values   10.651               9.597
P-values   0.000                0.000

 globID3 = 0.987*GID, Errorvar.= 0.767 , R≤ = 0.672
Standerr  (0.0767)             (0.113)
Z-values   12.873               6.807
P-values   0.000                0.000

 globID4 = 0.505*GID, Errorvar.= 0.926  , R≤ = 0.308
Standerr  (0.0586)             (0.0896)
Z-values   8.620                10.329
P-values   0.000                0.000


        Covariance Matrix of Independent Variables

               ETHNO      GREEN   AUTHENTI    QUALITY   LOCALISM   PRICESEN
            --------   --------   --------   --------   --------   --------
   ETHNO       1.426
             (0.189)
               7.557

   GREEN       0.628      1.014
             (0.103)    (0.133)
               6.104      7.625

AUTHENTI       0.848      0.538      1.069
             (0.115)    (0.088)    (0.142)
               7.388      6.129      7.512

 QUALITY       0.923      0.597      0.798      1.331
             (0.123)    (0.096)    (0.106)    (0.157)
               7.489      6.233      7.524      8.469
```

LOCALISM	0.884	0.481	0.887	0.674	1.333	
	(0.128)	(0.096)	(0.118)	(0.111)	(0.192)	
	6.889	5.022	7.521	6.074	6.933	
PRICESEN	-0.155	-0.173	-0.041	-0.040	0.029	1.031
	(0.089)	(0.079)	(0.072)	(0.079)	(0.082)	(0.249)
	-1.731	-2.176	-0.574	-0.507	0.357	4.145
NUTZEN	10.181	4.594	6.030	7.272	4.379	-6.853
	(1.903)	(1.528)	(1.543)	(1.714)	(1.730)	(1.929)
	5.349	3.007	3.907	4.243	2.531	-3.553
GLOBAL	-0.076	0.088	0.193	0.071	0.092	0.186
	(0.091)	(0.078)	(0.079)	(0.085)	(0.089)	(0.083)
	-0.834	1.125	2.432	0.835	1.039	2.227
GID	-0.034	0.366	0.033	-0.014	0.023	-0.011
	(0.111)	(0.100)	(0.094)	(0.104)	(0.108)	(0.091)
	-0.306	3.673	0.349	-0.139	0.215	-0.121

Covariance Matrix of Independent Variables

	NUTZEN	GLOBAL	GID
NUTZEN	466.543		
	(41.646)		
	11.203		
GLOBAL	-1.677	0.994	
	(1.545)	(0.174)	
	-1.086	5.702	
GID	1.755	0.330	1.611

(1.878)	(0.103)	(0.213)
0.934	3.195	7.556

Log-likelihood Values

	Estimated Model	Saturated Model
Number of free parameters(t)	97	496
-2ln(L)	9641.461	8866.917
AIC (Akaike, 1974)*	9835.461	9858.917
BIC (Schwarz, 1978)*	10177.430	11607.542

*LISREL uses AIC= 2t - 2ln(L) and BIC = tln(N)- 2ln(L)

Goodness-of-Fit Statistics

Degrees of Freedom for (C1)-(C2)	399
Maximum Likelihood Ratio Chi-Square (C1)	774.544 (P = 0.0000)
Browne's (1984) ADF Chi-Square (C2_NT)	785.412 (P = 0.0000)
Estimated Non-centrality Parameter (NCP)	375.544
90 Percent Confidence Interval for NCP	(300.519 ; 458.360)
Minimum Fit Function Value	3.086
Population Discrepancy Function Value (F0)	1.496
90 Percent Confidence Interval for F0	(1.197 ; 1.826)
Root Mean Square Error of Approximation (RMSEA)	0.0612
90 Percent Confidence Interval for RMSEA	(0.0548 ; 0.0677)
P-Value for Test of Close Fit (RMSEA < 0.05)	0.00246
Expected Cross-Validation Index (ECVI)	3.859
90 Percent Confidence Interval for ECVI	(3.560 ; 4.189)
ECVI for Saturated Model	3.952

ECVI for Independence Model 20.205

Chi-Square for Independence Model (465 df) 5009.425

Normed Fit Index (NFI) 0.845
Non-Normed Fit Index (NNFI) 0.904
Parsimony Normed Fit Index (PNFI) 0.725
Comparative Fit Index (CFI) 0.917
Incremental Fit Index (IFI) 0.919
Relative Fit Index (RFI) 0.820

Critical N (CN) 151.942

Root Mean Square Residual (RMR) 0.335
Standardized RMR 0.0620
Goodness of Fit Index (GFI) 0.832
Adjusted Goodness of Fit Index (AGFI) 0.791
Parsimony Goodness of Fit Index (PGFI) 0.669

!HV Daten aus Studie 1

Fitted Covariance Matrix

	cet1	cet2	cet3	cet4	env1	env2
cet1	2.151					
cet2	1.374	2.341				
cet3	1.446	1.394	2.116			
cet4	1.274	1.227	1.292	1.892		
env1	0.628	0.605	0.637	0.561	1.434	
env2	0.692	0.667	0.702	0.618	1.118	1.818
env4	0.605	0.583	0.613	0.540	0.977	1.077
aut1	0.848	0.817	0.860	0.757	0.538	0.593
aut2	0.864	0.833	0.877	0.772	0.548	0.605

aut3	0.874	0.843	0.887	0.781	0.555	0.611
aut4	0.909	0.876	0.922	0.812	0.577	0.636
qual1	0.923	0.890	0.936	0.825	0.597	0.658
qual2	0.936	0.902	0.949	0.836	0.605	0.667
qual3	0.762	0.734	0.773	0.680	0.492	0.543
qual4	0.870	0.838	0.882	0.777	0.562	0.620
qual5	0.759	0.731	0.769	0.678	0.490	0.541
qual6	0.715	0.689	0.725	0.638	0.462	0.509
localID1	0.884	0.852	0.897	0.790	0.481	0.531
localID2	0.886	0.854	0.899	0.791	0.482	0.531
localID3	0.774	0.746	0.785	0.691	0.421	0.464
localID4	0.688	0.663	0.697	0.614	0.374	0.413
tn_at	10.181	9.813	10.326	9.094	4.594	5.065
price1	-0.155	-0.149	-0.157	-0.138	-0.173	-0.191
price2	-0.240	-0.231	-0.244	-0.214	-0.268	-0.296
globatt1	-0.076	-0.073	-0.077	-0.068	0.088	0.097
globatt2	-0.066	-0.064	-0.067	-0.059	0.076	0.084
globatt3	-0.080	-0.077	-0.081	-0.072	0.093	0.103
globID1	-0.034	-0.033	-0.034	-0.030	0.366	0.403
globID2	-0.023	-0.022	-0.024	-0.021	0.252	0.277
globID3	-0.033	-0.032	-0.034	-0.030	0.361	0.398
globID4	-0.017	-0.017	-0.017	-0.015	0.185	0.204

Fitted Covariance Matrix

	env4	aut1	aut2	aut3	aut4	qual1
env4	1.788					
aut1	0.518	1.666				
aut2	0.528	1.090	1.423			
aut3	0.534	1.102	1.124	1.488		
aut4	0.556	1.146	1.169	1.182	1.672	
qual1	0.575	0.798	0.814	0.823	0.856	1.782
qual2	0.583	0.809	0.825	0.834	0.867	1.349
qual3	0.474	0.659	0.671	0.679	0.706	1.098

qual4	0.542	0.752	0.766	0.775	0.806	1.254
qual5	0.472	0.656	0.669	0.676	0.703	1.094
qual6	0.445	0.618	0.630	0.637	0.662	1.030
localID1	0.464	0.887	0.904	0.915	0.951	0.674
localID2	0.464	0.888	0.906	0.916	0.953	0.675
localID3	0.406	0.776	0.791	0.800	0.832	0.589
localID4	0.360	0.690	0.703	0.711	0.740	0.524
tn_at	4.426	6.030	6.147	6.218	6.467	7.272
price1	-0.167	-0.041	-0.042	-0.042	-0.044	-0.040
price2	-0.259	-0.064	-0.065	-0.066	-0.068	-0.062
globatt1	0.085	0.193	0.197	0.199	0.207	0.071
globatt2	0.074	0.168	0.171	0.173	0.180	0.062
globatt3	0.090	0.204	0.208	0.210	0.219	0.075
globID1	0.352	0.033	0.033	0.034	0.035	-0.014
globID2	0.242	0.022	0.023	0.023	0.024	-0.010
globID3	0.348	0.032	0.033	0.033	0.035	-0.014
globID4	0.178	0.016	0.017	0.017	0.018	-0.007

Fitted Covariance Matrix

	qual2	qual3	qual4	qual5	qual6	localID1
qual2	1.699					
qual3	1.113	1.829				
qual4	1.270	1.035	1.680			
qual5	1.108	0.903	1.030	1.439		
qual6	1.044	0.850	0.970	0.847	1.693	
localID1	0.683	0.556	0.634	0.554	0.521	2.235
localID2	0.684	0.557	0.636	0.555	0.522	1.335
localID3	0.597	0.486	0.555	0.484	0.456	1.166
localID4	0.531	0.432	0.493	0.430	0.405	1.036
tn_at	7.370	6.001	6.849	5.976	5.628	4.379
price1	-0.041	-0.033	-0.038	-0.033	-0.031	0.029
price2	-0.063	-0.052	-0.059	-0.051	-0.048	0.045
globatt1	0.072	0.059	0.067	0.059	0.055	0.092

globatt2	0.063	0.051	0.058	0.051	0.048	0.080
globatt3	0.076	0.062	0.071	0.062	0.058	0.098
globID1	-0.015	-0.012	-0.014	-0.012	-0.011	0.023
globID2	-0.010	-0.008	-0.009	-0.008	-0.008	0.016
globID3	-0.014	-0.012	-0.013	-0.012	-0.011	0.023
globID4	-0.007	-0.006	-0.007	-0.006	-0.006	0.012

Fitted Covariance Matrix

	localID2	localID3	localID4	tn_at	price1	price2
localID2	2.262					
localID3	1.168	1.506				
localID4	1.038	0.907	1.423			
tn_at	4.387	3.831	3.405	466.543		
price1	0.029	0.026	0.023	-6.853	2.312	
price2	0.046	0.040	0.035	-10.628	1.599	2.638
globatt1	0.093	0.081	0.072	-1.677	0.186	0.288
globatt2	0.080	0.070	0.062	-1.457	0.161	0.250
globatt3	0.098	0.085	0.076	-1.772	0.196	0.304
globID1	0.023	0.020	0.018	1.755	-0.011	-0.017
globID2	0.016	0.014	0.012	1.208	-0.008	-0.012
globID3	0.023	0.020	0.018	1.733	-0.011	-0.017
globID4	0.012	0.010	0.009	0.887	-0.006	-0.009

Fitted Covariance Matrix

	globatt1	globatt2	globatt3	globID1	globID2	globID3
globatt1	1.782					
globatt2	0.863	2.507				
globatt3	1.050	0.912	1.690			
globID1	0.330	0.287	0.349	2.257		
globID2	0.227	0.198	0.240	1.109	1.707	
globID3	0.326	0.283	0.345	1.590	1.095	2.337

globID4	0.167	0.145	0.176	0.814	0.560	0.804

Fitted Covariance Matrix

	globID4
globID4	1.337

Fitted Residuals

	cet1	cet2	cet3	cet4	env1	env2
cet1	0.000					
cet2	0.145	0.000				
cet3	0.015	-0.060	0.000			
cet4	-0.068	-0.114	0.057	0.000		
env1	-0.080	-0.037	0.097	0.082	0.000	
env2	-0.105	0.004	0.037	0.082	0.000	0.000
env4	-0.118	0.024	-0.044	-0.027	-0.017	0.020
aut1	-0.057	0.098	-0.056	0.004	0.128	-0.056
aut2	-0.028	0.043	0.018	0.025	0.117	-0.067
aut3	-0.010	0.022	-0.057	-0.023	0.048	-0.136
aut4	0.067	0.029	-0.040	0.034	0.042	-0.069
qual1	-0.032	0.036	-0.038	0.020	-0.013	-0.080
qual2	-0.090	-0.037	0.089	0.027	0.077	0.002
qual3	-0.109	-0.161	-0.073	0.208	0.035	-0.113
qual4	0.037	0.005	0.175	0.084	0.108	-0.055
qual5	-0.133	-0.146	-0.069	0.090	0.071	-0.044
qual6	0.020	-0.112	-0.061	0.043	0.037	-0.055
localID1	-0.147	-0.071	-0.052	0.097	0.072	-0.044
localID2	0.099	0.050	0.017	0.051	0.061	-0.127
localID3	-0.047	0.024	-0.045	0.124	0.070	-0.083
localID4	-0.067	0.007	-0.058	0.087	0.106	-0.060
tn_at	-0.616	0.691	-1.547	2.206	-1.532	1.940
price1	-0.112	-0.011	-0.020	-0.442	-0.033	-0.039

price2	0.045	-0.004	0.153	-0.203	0.076	-0.132
globatt1	-0.093	-0.135	-0.056	0.122	0.055	-0.050
globatt2	-0.072	0.033	-0.023	-0.003	-0.093	-0.149
globatt3	-0.024	-0.045	0.010	0.192	0.027	-0.001
globID1	-0.129	-0.127	-0.075	-0.030	-0.199	-0.084
globID2	0.112	-0.096	0.134	0.213	0.099	0.115
globID3	-0.027	-0.191	-0.028	0.075	-0.227	-0.036
globID4	0.134	0.137	0.182	0.323	0.317	0.387

Fitted Residuals

	env4	aut1	aut2	aut3	aut4	qual1
env4	0.000					
aut1	0.098	0.000				
aut2	0.017	-0.008	0.000			
aut3	-0.107	0.030	0.012	0.000		
aut4	-0.085	-0.022	-0.009	-0.008	0.000	
qual1	-0.050	-0.039	-0.038	-0.078	0.010	0.000
qual2	-0.036	0.035	0.021	0.016	0.039	0.042
qual3	-0.024	0.033	-0.040	-0.051	0.054	-0.019
qual4	-0.112	-0.018	0.058	0.021	0.030	0.024
qual5	0.029	0.039	-0.010	-0.039	0.030	-0.064
qual6	-0.003	-0.095	-0.030	-0.059	-0.015	-0.011
localID1	-0.101	-0.134	-0.157	-0.060	-0.011	-0.018
localID2	0.029	0.011	-0.018	0.030	0.149	0.147
localID3	-0.041	-0.050	-0.009	-0.015	0.029	0.063
localID4	-0.010	0.036	0.074	0.032	0.084	0.033
tn_at	-0.006	0.498	-0.213	-0.215	0.203	-1.281
price1	0.114	0.056	-0.041	0.074	0.016	-0.119
price2	0.061	0.087	-0.034	0.008	-0.031	0.036
globatt1	-0.088	-0.128	0.016	-0.118	-0.046	-0.075
globatt2	-0.124	0.095	-0.061	-0.048	-0.095	0.049
globatt3	0.137	0.182	0.066	-0.037	0.054	-0.014
globID1	0.096	-0.076	-0.067	-0.101	-0.262	-0.042

globID2	0.248	0.245	0.215	0.094	0.121	0.074
globID3	0.034	-0.034	-0.056	-0.042	-0.177	-0.005
globID4	0.313	0.335	0.355	0.275	0.294	0.213

Fitted Residuals

	qual2	qual3	qual4	qual5	qual6	localID1
qual2	0.000					
qual3	-0.048	0.000				
qual4	0.013	-0.067	0.000			
qual5	-0.050	0.192	-0.010	0.000		
qual6	-0.035	0.052	-0.087	0.193	0.000	
localID1	-0.067	-0.079	-0.077	-0.084	-0.042	0.000
localID2	-0.017	-0.025	0.053	-0.063	-0.156	0.051
localID3	-0.027	0.045	0.046	0.053	0.057	0.062
localID4	-0.091	0.005	0.052	0.096	-0.053	-0.065
tn_at	0.646	-0.326	0.473	0.588	-0.729	-1.579
price1	0.036	-0.124	-0.130	-0.204	-0.212	0.142
price2	0.027	0.007	0.029	-0.079	-0.029	0.231
globatt1	-0.068	0.243	-0.108	0.008	-0.019	0.069
globatt2	0.022	-0.173	0.025	-0.100	-0.032	0.116
globatt3	0.075	0.111	-0.085	0.078	0.039	-0.021
globID1	-0.059	-0.191	-0.058	-0.134	-0.131	-0.122
globID2	0.141	0.194	0.136	0.236	0.215	0.116
globID3	-0.061	-0.221	-0.035	-0.091	-0.020	-0.245
globID4	0.156	0.200	0.184	0.172	0.097	0.174

Fitted Residuals

	localID2	localID3	localID4	tn_at	price1	price2
localID2	0.000					
localID3	-0.077	0.000				
localID4	0.006	0.022	0.000			

tn_at	-1.076	-0.099	2.458	0.000		
price1	0.160	-0.002	-0.149	0.122	0.000	
price2	0.142	-0.096	-0.197	-0.010	0.000	0.000
globatt1	0.016	0.021	-0.101	-1.475	-0.034	-0.079
globatt2	0.228	-0.220	-0.188	-3.059	0.330	0.512
globatt3	0.066	-0.012	0.032	1.862	-0.007	-0.089
globID1	-0.222	-0.087	-0.020	-1.870	0.097	0.034
globID2	0.037	0.216	0.344	0.512	0.115	0.179
globID3	-0.096	-0.066	-0.023	1.432	-0.038	-0.097
globID4	0.296	0.274	0.386	1.234	-0.202	-0.116

Fitted Residuals

	globatt1	globatt2	globatt3	globID1	globID2	globID3
globatt1	0.000					
globatt2	-0.005	0.000				
globatt3	0.018	-0.043	0.000			
globID1	0.025	0.167	-0.027	0.000		
globID2	0.009	0.195	0.246	-0.043	0.000	
globID3	-0.243	0.091	-0.112	0.053	-0.023	0.000
globID4	0.113	-0.048	0.033	-0.046	0.096	-0.050

Fitted Residuals

	globID4
globID4	0.000

Summary Statistics for Fitted Residuals

```
Smallest Fitted Residual =   -3.059
  Median Fitted Residual =    0.000
 Largest Fitted Residual =    2.458
```

Stemleaf Plot

```
- 3|1
- 2|
- 2|
- 1|96555
- 1|31
- 0|76
- 0|4332222222222222222222222111111111111111111111111111111111111111111111111+95
  0|111111111111111111111111111111111111111111111111111111111111111111111111+54
  0|5555667
  1|24
  1|99
  2|2
  2|5
```

Standardized Residuals

	cet1	cet2	cet3	cet4	env1	env2
cet1	0.000					
cet2	0.889	0.000				
cet3	0.079	-0.361	- -			
cet4	-0.505	-0.745	0.435	0.000		
env1	-0.677	-0.304	- -	- -	0.000	
env2	-0.794	- -	0.270	0.663	- -	0.000
env4	-1.048	0.208	- -	-0.224	-0.359	0.289
aut1	-0.433	0.769	-0.428	0.031	1.241	-0.461
aut2	- -	0.506	0.164	0.219	1.260	-0.611
aut3	-0.081	- -	-0.603	-0.250	0.475	-1.271
aut4	0.536	0.208	-0.296	- -	0.485	-0.775
qual1	-0.226	0.283	-0.281	0.180	-0.113	-0.816
qual2	-0.811	- -	0.929	0.270	0.732	0.019
qual3	-0.811	-1.466	-0.607	1.660	0.324	-1.002
qual4	0.284	0.044	1.332	0.684	1.037	- -

qual5	- -	-1.173	-0.559	0.803	- -	-0.410
qual6	0.159	-0.837	-0.474	0.357	0.363	-0.519
localID1	-1.033	-0.443	-0.352	1.090	0.630	-0.524
localID2	0.662	0.322	0.116	0.362	0.511	-1.577
localID3	-0.375	0.190	-0.364	1.076	0.726	-0.767
localID4	- -	0.065	-0.495	0.784	- -	-0.567
tn_at	-0.339	0.319	-0.741	1.124	-0.924	1.040
price1	-0.870	-0.074	-0.143	-3.339	-0.286	-0.400
price2	0.298	-0.023	1.017	-1.429	0.616	-0.893
globatt1	-0.752	-1.044	-0.454	1.055	0.556	-0.334
globatt2	-0.491	0.213	-0.154	-0.022	-0.842	-1.107
globatt3	-0.197	-0.415	0.087	1.697	0.274	-0.005
globID1	-0.927	-0.877	- -	-0.228	-2.060	-0.641
globID2	0.929	-0.757	1.128	1.876	1.069	1.021
globID3	-0.231	-1.297	-0.212	0.564	-1.979	-0.272
globID4	1.253	1.222	1.701	- -	5.521	- -

Standardized Residuals

	env4	aut1	aut2	aut3	aut4	qual1
env4	0.000					
aut1	0.856	0.000				
aut2	0.157	-0.065	0.000			
aut3	-1.160	0.245	0.105	0.000		
aut4	-0.779	-0.176	-0.071	-0.067	0.000	
qual1	- -	-0.326	-0.321	-0.677	0.083	- -
qual2	-0.307	0.364	0.193	0.141	0.322	0.290
qual3	-0.207	0.285	-0.415	-0.472	0.488	-0.153
qual4	-1.011	-0.199	- -	0.187	0.254	0.181
qual5	0.271	0.369	-0.096	-0.383	- -	-0.518
qual6	-0.026	-0.933	- -	-0.974	-0.135	-0.088
localID1	-0.777	-0.992	-1.245	-0.458	-0.082	-0.129
localID2	- -	0.104	-0.140	0.234	1.090	1.101
localID3	-0.381	-0.694	-0.085	-0.182	1.534	0.570

localID4	-0.127	- -	0.738	0.344	0.811	- -
tn_at	-0.003	0.277	-0.128	-0.127	0.156	-0.682
price1	0.883	0.490	-0.358	0.636	0.130	-1.030
price2	0.120	0.652	-0.273	0.084	-0.269	0.268
globatt1	-0.746	-1.135	0.166	-1.200	-0.421	-0.670
globatt2	-0.925	0.737	-0.512	-0.776	-0.731	0.367
globatt3	1.254	- -	0.671	-0.369	0.687	-0.139
globID1	0.751	-0.618	-0.598	-0.870	-2.136	-0.328
globID2	2.508	2.294	- -	1.066	1.140	0.671
globID3	0.293	-0.274	-0.507	-0.359	-1.545	-0.037
globID4	3.186	3.553	4.079	3.089	3.223	2.160

Standardized Residuals

	qual2	qual3	qual4	qual5	qual6	localID1
qual2	- -					
qual3	-0.523	0.000				
qual4	0.094	-0.518	0.000			
qual5	-0.436	- -	-0.113	0.000		
qual6	-0.278	0.417	-0.710	1.726	0.000	
localID1	-0.512	-0.651	-0.581	-1.056	-0.333	0.000
localID2	-0.150	- -	0.412	-0.526	-1.503	0.308
localID3	-0.253	0.409	0.734	0.539	0.644	0.499
localID4	-0.875	0.048	0.525	1.013	-0.527	- -
tn_at	0.353	-0.173	0.260	0.354	-0.403	-0.768
price1	0.283	-0.949	-0.353	-1.771	-1.696	0.990
price2	0.203	0.047	0.217	-0.640	-0.215	1.504
globatt1	-0.532	2.121	-1.034	0.065	-0.210	0.676
globatt2	0.170	-1.366	0.190	-0.844	-0.263	0.799
globatt3	0.707	0.925	-0.543	0.792	0.371	-0.168
globID1	-0.481	-1.539	-0.463	-1.221	-2.097	-0.862
globID2	1.312	1.741	1.276	3.058	2.019	0.941
globID3	-0.474	-1.695	-0.414	-0.789	-0.159	-1.697
globID4	2.589	2.023	2.433	1.947	1.023	1.598

Standardized Residuals

	localID2	localID3	localID4	tn_at	price1	price2
localID2	- -					
localID3	- -	0.000				
localID4	0.043	0.205	0.000			
tn_at	-0.520	-0.059	1.513	0.000		
price1	1.106	-0.014	-1.370	0.022	0.000	
price2	0.913	- -	-1.624	-0.005	0.000	0.000
globatt1	0.122	0.205	-1.002	-0.809	-0.260	-0.571
globatt2	1.513	-1.810	-1.580	-1.416	2.162	3.216
globatt3	0.536	-0.119	0.136	1.156	-0.056	-0.658
globID1	-1.557	-0.750	-0.177	-0.912	0.672	0.220
globID2	0.300	2.732	3.840	0.288	0.913	1.335
globID3	-0.661	-0.575	-0.198	0.664	-0.261	-0.622
globID4	2.699	3.056	4.432	0.782	-1.824	-0.965

Standardized Residuals

	globatt1	globatt2	globatt3	globID1	globID2	globID3
globatt1	0.000					
globatt2	-0.037	0.000				
globatt3	0.142	-0.303	0.000			
globID1	0.194	1.057	-0.204	0.000		
globID2	0.077	1.491	2.578	-0.299	0.000	
globID3	-1.843	0.616	-0.883	0.297	-0.164	0.000
globID4	1.200	-0.413	0.569	-2.021	0.954	-0.406

Standardized Residuals

	globID4

```
 globID4      0.000

Summary Statistics for Standardized Residuals

Smallest Standardized Residual =   -3.339
  Median Standardized Residual =    0.000
 Largest Standardized Residual =    5.521

Stemleaf Plot

- 3|3
- 2|
- 2|11100
- 1|888877766665555
- 1|4444332222211100000000000
- 0|999999999999988888888888888888777777777777776666666666666555555555555555+09
- 0|444444444444444444444444333333333333333333333332222222222222222222222111+98
  0|111111111111111112222222222222222222222223333333333333333333333333444444+07
  0|55555555555666666666777777777777777788888888999999999
  1|0000000011111111111122223333333
  1|5555567777799
  2|0012234
  2|56677
  3|111222
  3|68
  4|14
  4|
  5|
  5|5
Largest Negative Standardized Residuals
Residual for   price1 and     cet4  -3.339
Largest Positive Standardized Residuals
Residual for globatt2 and   price2   3.216
Residual for  globID2 and    qual5   3.058
Residual for  globID2 and localID3   2.732
```

```
Residual for  globID2 and localID4   3.840
Residual for  globID2 and globatt3   2.578
Residual for  globID4 and     env1   5.521
Residual for  globID4 and     env4   3.186
Residual for  globID4 and     aut1   3.553
Residual for  globID4 and     aut2   4.079
Residual for  globID4 and     aut3   3.089
Residual for  globID4 and     aut4   3.223
Residual for  globID4 and    qual2   2.589
Residual for  globID4 and localID2   2.699
Residual for  globID4 and localID3   3.056
Residual for  globID4 and localID4   4.432

!HV Daten aus Studie 1

                        Qplot of Standardized Residuals

 3.5..........................................................................
    .                                                                       ..
    .                                                                      . .
    .                                                                    .   .
    .                                                                  .     .
    .                                                                 .      .
    .                                                               .        *
    .                                                             .          x
    .                                                            .        x  *
    .                                                          .          *  .
    .                                                        .       x  *x   .
    .                                                       .      x x       .
    .                                                     .   *xx x          .
    .                                                   .x*xxx               .
    .                                                 *.x                    .
N   .                                               **x                      .
o   .                                             **x                        .
r   .                                          xx*x                          .
```

```
m   .                                         **.                            .
a   .                                       xx*                              .
l   .                                      x*.                               .
    .                                     *x                                 .
Q   .                                   xx                                   .
u   .                                  x*                                    .
a   .                                 *x                                     .
n   .                               .x*                                      .
t   .                              xx                                        .
i   .                            .xx                                         .
l   .                          . *x                                          .
e   .                         .x*                                            .
s   .                       . *x                                             .
    .                     . xxx                                              .
    .                    .***                                                .
    .                  .x**                                                  .
    .                . xx                                                    .
    .               . **                                                     .
    .             .  *                                                       .
    .           .  *x                                                        .
    .          .   x                                                         .
    . x      .    x                                                          .
    .      .                                                                 .
    .     .                                                                  .
    .   .                                                                    .
    . .                                                                      .
-3.5..........................................................................
  -3.5                                                                      3.5
                            Standardized Residuals
```

!HV Daten aus Studie 1

Modification Indices and Expected Change

The Modification Indices Suggest to Add the

Path to	from	Decrease in Chi-Square	New Estimate
cet4	PRICESEN	8.2	-0.18
env1	AUTHENTI	9.5	0.22
localID4	PRICESEN	7.9	-0.16
globatt2	PRICESEN	17.0	0.38
globID1	AUTHENTI	8.7	-0.20
globID2	LOCALISM	9.1	0.18
globID4	ETHNO	10.8	0.18
globID4	GREEN	30.7	0.40
globID4	AUTHENTI	24.5	0.31
globID4	QUALITY	8.8	0.17
globID4	LOCALISM	21.2	0.27

Modification Indices for LAMBDA-X

	ETHNO	GREEN	AUTHENTI	QUALITY	LOCALISM	PRICESEN
cet1	- -	4.989	0.214	2.308	0.995	0.225
cet2	- -	0.106	0.631	0.864	0.055	0.005
cet3	- -	1.143	0.799	0.718	0.977	4.529
cet4	- -	2.351	0.548	2.494	3.872	8.243
env1	1.047	- -	9.477	4.553	7.586	2.213
env2	0.039	- -	6.396	2.045	5.058	4.723
env4	0.960	- -	0.486	0.742	0.436	0.625
aut1	0.121	1.559	- -	0.001	0.970	1.358
aut2	0.100	1.498	- -	0.008	0.919	0.547
aut3	0.625	2.551	- -	0.932	0.028	0.063
aut4	0.617	0.498	- -	0.774	4.173	0.206
qual1	0.115	1.303	1.140	- -	1.473	0.223
qual2	0.008	0.789	0.727	- -	1.765	0.295
qual3	0.459	0.369	0.020	- -	0.029	0.000
qual4	5.084	0.072	0.755	- -	0.654	0.055
qual5	1.759	0.198	0.000	- -	0.003	1.635
qual6	0.317	0.010	0.896	- -	0.502	0.199
localID1	1.521	0.215	6.250	1.606	- -	7.295

localID2	0.803	0.056	1.232	0.107	- -	2.942
localID3	0.079	0.000	0.080	0.655	- -	2.376
localID4	0.001	0.564	3.111	0.003	- -	7.898
tn_at	- -	- -	- -	- -	- -	- -
price1	2.240	0.038	0.017	1.461	0.041	- -
price2	2.240	0.038	0.017	1.461	0.041	- -
globatt1	0.610	0.125	1.295	0.583	0.051	1.116
globatt2	0.195	1.683	0.293	0.030	0.215	16.991
globatt3	1.067	1.401	2.094	0.712	0.269	2.928
globID1	5.199	6.487	8.749	4.427	6.988	0.467
globID2	3.233	5.396	7.755	6.038	9.129	3.739
globID3	0.594	4.799	2.926	1.571	4.053	1.840
globID4	10.789	30.735	24.476	8.801	21.156	1.829

Modification Indices for LAMBDA-X

	NUTZEN	GLOBAL	GID
cet1	0.480	1.064	0.249
cet2	0.378	1.018	2.342
cet3	3.592	0.077	0.054
cet4	5.392	5.757	2.989
env1	5.616	0.453	6.218
env2	5.890	1.246	0.497
env4	0.000	0.270	4.956
aut1	0.288	1.645	1.033
aut2	0.124	0.898	1.581
aut3	0.107	3.079	0.010
aut4	0.072	0.071	4.474
qual1	2.788	0.392	0.179
qual2	1.095	0.324	0.040
qual3	0.074	2.715	1.129
qual4	0.324	2.726	0.053
qual5	0.434	0.298	0.035
qual6	0.376	0.004	0.004

localID1	2.008	0.177	1.336
localID2	0.905	1.328	1.061
localID3	0.017	0.553	0.169
localID4	6.865	0.595	3.234
tn_at	- -	- -	- -
price1	0.019	0.299	0.074
price2	0.019	0.299	0.074
globatt1	2.311	- -	0.958
globatt2	3.267	- -	1.407
globatt3	6.917	- -	0.021
globID1	5.119	0.107	- -
globID2	0.174	6.840	- -
globID3	2.233	7.764	- -
globID4	0.960	0.904	- -

Expected Change for LAMBDA-X

	ETHNO	GREEN	AUTHENTI	QUALITY	LOCALISM	PRICESEN
cet1	- -	-0.190	-0.046	-0.131	-0.085	0.031
cet2	- -	-0.031	0.087	-0.088	0.022	-0.005
cet3	- -	0.089	-0.088	0.072	-0.083	0.135
cet4	- -	0.127	0.071	0.131	0.163	-0.182
env1	0.065	- -	0.220	0.135	0.163	0.084
env2	-0.014	- -	-0.202	-0.102	-0.150	-0.138
env4	-0.071	- -	-0.057	-0.062	-0.046	0.053
aut1	-0.025	0.087	- -	0.002	-0.083	0.063
aut2	0.019	0.069	- -	0.005	-0.066	-0.032
aut3	-0.048	-0.093	- -	-0.056	-0.012	0.011
aut4	0.052	-0.045	- -	0.056	0.158	-0.022
qual1	-0.022	-0.072	-0.075	- -	0.066	0.023
qual2	0.005	0.052	0.055	- -	-0.066	0.024
qual3	-0.055	-0.050	-0.013	- -	-0.012	0.000
qual4	0.146	0.017	0.062	- -	0.045	0.012
qual5	-0.086	0.029	0.000	- -	0.003	-0.065

qual6	-0.045	-0.008	-0.084	- -	-0.049	-0.028
localID1	-0.110	-0.039	-0.308	-0.096	- -	0.188
localID2	0.080	-0.020	0.138	0.025	- -	0.120
localID3	0.020	-0.001	-0.028	0.048	- -	-0.084
localID4	0.003	0.051	0.175	0.003	- -	-0.159
tn_at	- -	- -	- -	- -	- -	- -
price1	-0.103	-0.017	0.010	-0.080	0.014	- -
price2	0.160	0.026	-0.015	0.124	-0.021	- -
globatt1	-0.048	-0.027	-0.083	-0.048	-0.015	-0.078
globatt2	-0.035	-0.125	-0.050	-0.014	-0.038	0.383
globatt3	0.064	0.090	0.106	0.054	0.034	-0.128
globID1	-0.136	-0.197	-0.200	-0.127	-0.164	0.047
globID2	0.105	0.174	0.185	0.146	0.184	0.130
globID3	-0.047	-0.173	-0.118	-0.077	-0.128	-0.095
globID4	0.184	0.397	0.314	0.169	0.268	-0.087

Expected Change for LAMBDA-X

	NUTZEN	GLOBAL	GID
cet1	-0.002	-0.074	-0.027
cet2	0.002	-0.081	-0.092
cet3	-0.006	-0.019	0.012
cet4	0.007	0.168	0.091
env1	-0.006	0.041	-0.123
env2	0.007	-0.078	0.039
env4	0.000	0.038	0.131
aut1	0.001	0.079	0.046
aut2	-0.001	0.047	0.046
aut3	-0.001	-0.090	-0.004
aut4	0.001	-0.015	-0.087
qual1	-0.004	-0.034	0.017
qual2	0.002	0.028	0.008
qual3	-0.001	0.118	-0.057
qual4	0.001	-0.092	0.010

qual5	0.002	0.031	-0.008
qual6	-0.002	0.005	-0.003
localID1	-0.005	0.033	-0.068
localID2	-0.003	0.090	-0.061
localID3	0.000	-0.045	0.019
localID4	0.007	-0.049	0.085
tn_at	- -	- -	- -
price1	0.001	0.050	0.017
price2	-0.001	-0.078	-0.026
globatt1	-0.005	- -	-0.063
globatt2	-0.008	- -	0.095
globatt3	0.009	- -	0.009
globID1	-0.007	0.026	- -
globID2	0.001	0.206	- -
globID3	0.005	-0.230	- -
globID4	0.003	0.071	- -

Standardized Expected Change for LAMBDA-X

	ETHNO	GREEN	AUTHENTI	QUALITY	LOCALISM	PRICESEN
cet1	- -	-0.191	-0.048	-0.151	-0.099	0.031
cet2	- -	-0.031	0.090	-0.101	0.026	-0.005
cet3	- -	0.090	-0.091	0.083	-0.096	0.137
cet4	- -	0.128	0.074	0.152	0.188	-0.185
env1	0.078	- -	0.227	0.156	0.189	0.085
env2	-0.017	- -	-0.209	-0.117	-0.173	-0.140
env4	-0.085	- -	-0.059	-0.072	-0.053	0.054
aut1	-0.030	0.088	- -	0.003	-0.096	0.064
aut2	0.022	0.069	- -	0.006	-0.076	-0.032
aut3	-0.057	-0.094	- -	-0.065	-0.014	0.011
aut4	0.062	-0.045	- -	0.064	0.183	-0.023
qual1	-0.026	-0.073	-0.078	- -	0.076	0.024
qual2	0.006	0.052	0.057	- -	-0.076	0.025
qual3	-0.066	-0.050	-0.013	- -	-0.014	0.000

qual4	0.174	0.017	0.065	- -	0.052	0.012
qual5	-0.102	0.029	0.000	- -	0.004	-0.066
qual6	-0.054	-0.008	-0.087	- -	-0.056	-0.028
localID1	-0.131	-0.039	-0.319	-0.110	- -	0.191
localID2	0.096	-0.020	0.142	0.029	- -	0.122
localID3	0.024	-0.001	-0.029	0.056	- -	-0.086
localID4	0.003	0.052	0.181	0.004	- -	-0.161
tn_at	- -	- -	- -	- -	- -	- -
price1	-0.123	-0.017	0.010	-0.092	0.016	- -
price2	0.191	0.026	-0.016	0.143	-0.025	- -
globatt1	-0.058	-0.027	-0.085	-0.055	-0.017	-0.079
globatt2	-0.042	-0.125	-0.051	-0.016	-0.044	0.389
globatt3	0.077	0.091	0.110	0.062	0.039	-0.130
globID1	-0.162	-0.198	-0.207	-0.147	-0.189	0.047
globID2	0.126	0.175	0.191	0.168	0.213	0.132
globID3	-0.056	-0.174	-0.122	-0.089	-0.148	-0.096
globID4	0.220	0.399	0.325	0.194	0.310	-0.088

Standardized Expected Change for LAMBDA-X

	NUTZEN	GLOBAL	GID
cet1	-0.049	-0.074	-0.034
cet2	0.048	-0.080	-0.117
cet3	-0.130	-0.019	0.016
cet4	0.159	0.168	0.116
env1	-0.133	0.041	-0.156
env2	0.154	-0.077	0.050
env4	0.000	0.038	0.166
aut1	0.030	0.079	0.059
aut2	-0.016	0.046	0.058
aut3	-0.015	-0.089	-0.005
aut4	0.014	-0.015	-0.111
qual1	-0.085	-0.034	0.022
qual2	0.049	0.028	0.010

qual3	-0.018	0.117	-0.073
qual4	0.030	-0.092	0.012
qual5	0.034	0.031	-0.010
qual6	-0.040	0.005	-0.004
localID1	-0.099	0.033	-0.086
localID2	-0.067	0.090	-0.077
localID3	-0.007	-0.045	0.024
localID4	0.149	-0.049	0.108
tn_at	- -	- -	- -
price1	0.018	0.050	0.022
price2	-0.027	-0.078	-0.033
globatt1	-0.107	- -	-0.080
globatt2	-0.162	- -	0.121
globatt3	0.187	- -	0.012
globID1	-0.153	0.026	- -
globID2	0.028	0.205	- -
globID3	0.103	-0.229	- -
globID4	0.062	0.071	- -

Completely Standardized Expected Change for LAMBDA-X

	ETHNO	GREEN	AUTHENTI	QUALITY	LOCALISM	PRICESEN
cet1	- -	-0.131	-0.033	-0.103	-0.067	0.021
cet2	- -	-0.020	0.059	-0.066	0.017	-0.003
cet3	- -	0.062	-0.062	0.057	-0.066	0.094
cet4	- -	0.093	0.054	0.110	0.137	-0.135
env1	0.065	- -	0.190	0.130	0.158	0.071
env2	-0.013	- -	-0.155	-0.087	-0.128	-0.104
env4	-0.064	- -	-0.044	-0.054	-0.039	0.040
aut1	-0.023	0.068	- -	0.002	-0.074	0.050
aut2	0.019	0.058	- -	0.005	-0.064	-0.027
aut3	-0.047	-0.077	- -	-0.053	-0.011	0.009
aut4	0.048	-0.035	- -	0.050	0.141	-0.018
qual1	-0.019	-0.054	-0.058	- -	0.057	0.018

qual2	0.005	0.040	0.044	- -	-0.059	0.019
qual3	-0.049	-0.037	-0.010	- -	-0.010	0.000
qual4	0.134	0.013	0.050	- -	0.040	0.009
qual5	-0.085	0.024	0.000	- -	0.003	-0.055
qual6	-0.041	-0.006	-0.067	- -	-0.043	-0.022
localID1	-0.088	-0.026	-0.213	-0.074	- -	0.128
localID2	0.064	-0.013	0.095	0.019	- -	0.081
localID3	0.019	-0.001	-0.024	0.046	- -	-0.070
localID4	0.003	0.043	0.152	0.003	- -	-0.135
tn_at	- -	- -	- -	- -	- -	- -
price1	-0.081	-0.011	0.007	-0.061	0.010	- -
price2	0.118	0.016	-0.010	0.088	-0.015	- -
globatt1	-0.043	-0.020	-0.064	-0.041	-0.013	-0.059
globatt2	-0.026	-0.079	-0.032	-0.010	-0.028	0.245
globatt3	0.059	0.070	0.085	0.048	0.030	-0.100
globID1	-0.108	-0.132	-0.138	-0.098	-0.126	0.032
globID2	0.096	0.134	0.146	0.129	0.163	0.101
globID3	-0.037	-0.114	-0.080	-0.058	-0.097	-0.063
globID4	0.190	0.345	0.281	0.168	0.268	-0.076

Completely Standardized Expected Change for LAMBDA-X

	NUTZEN	GLOBAL	GID
cet1	-0.033	-0.050	-0.023
cet2	0.031	-0.052	-0.076
cet3	-0.090	-0.013	0.011
cet4	0.116	0.122	0.084
env1	-0.111	0.035	-0.130
env2	0.114	-0.057	0.037
env4	0.000	0.029	0.124
aut1	0.023	0.061	0.045
aut2	-0.013	0.039	0.049
aut3	-0.012	-0.073	-0.004
aut4	0.010	-0.012	-0.086

qual1	-0.064	-0.026	0.017
qual2	0.037	0.022	0.007
qual3	-0.013	0.087	-0.054
qual4	0.023	-0.071	0.010
qual5	0.029	0.026	-0.008
qual6	-0.031	0.004	-0.003
localID1	-0.066	0.022	-0.057
localID2	-0.045	0.060	-0.051
localID3	-0.006	-0.037	0.020
localID4	0.125	-0.041	0.091
tn_at	- -	- -	- -
price1	0.012	0.033	0.014
price2	-0.017	-0.048	-0.021
globatt1	-0.080	- -	-0.060
globatt2	-0.102	- -	0.076
globatt3	0.143	- -	0.009
globID1	-0.102	0.018	- -
globID2	0.021	0.157	- -
globID3	0.068	-0.150	- -
globID4	0.054	0.062	- -

No Non-Zero Modification Indices for PHI

The Modification Indices Suggest to Add an Error Covariance

Between	and	Decrease in Chi-Square	New Estimate
cet2	cet1	13.3	0.27
qual2	qual1	7.9	0.11
qual3	cet4	16.0	0.24
qual5	qual3	23.5	0.24
qual6	qual5	24.2	0.24
localID3	localID2	8.1	-0.19
localID4	qual2	8.5	-0.11
localID4	qual5	8.4	0.12
price1	cet4	13.6	-0.26
price1	qual2	11.1	0.17

globatt1	aut1	13.7	-0.20
globatt1	qual3	18.0	0.28
globatt2	qual3	10.0	-0.28
globatt2	localID2	9.8	0.29
globatt2	localID3	11.7	-0.24
globatt2	price2	8.3	0.30
globatt3	aut1	9.7	0.16
globID2	qual5	8.2	0.15
globID2	localID4	10.3	0.18
globID2	globatt3	10.4	0.21
globID3	globID1	18.4	0.69

Modification Indices for THETA-DELTA

	cet1	cet2	cet3	cet4	env1	env2
cet1	- -					
cet2	13.257	- -				
cet3	0.307	2.744	- -			
cet4	4.169	7.036	3.666	- -		
env1	1.458	2.436	2.435	0.008	- -	
env2	0.226	0.455	0.058	0.641	0.002	- -
env4	0.001	2.647	0.642	0.889	0.856	0.776
aut1	0.836	3.450	0.403	0.061	0.289	0.971
aut2	0.844	0.050	2.762	0.035	0.948	1.134
aut3	0.770	0.096	0.352	1.339	0.478	0.709
aut4	4.415	0.433	1.855	0.104	0.638	1.734
qual1	0.983	5.750	4.138	0.862	3.299	0.017
qual2	2.871	0.004	7.711	1.734	0.000	1.910
qual3	0.651	3.215	2.321	16.029	0.239	1.341
qual4	0.260	0.411	7.633	0.951	4.570	0.538
qual5	1.936	2.954	2.207	5.621	0.163	0.563
qual6	3.731	1.075	1.823	0.205	0.005	0.137
localID1	2.364	0.426	0.586	2.601	0.224	2.672
localID2	5.215	0.006	0.073	1.512	0.056	1.069

localID3	0.743	0.049	0.929	3.189	0.013	0.295
localID4	1.449	0.002	1.254	0.675	0.554	0.901
tn_at	0.532	0.596	2.020	2.808	5.521	4.418
price1	0.174	2.346	0.013	13.607	3.205	1.457
price2	0.297	0.561	2.080	0.035	2.310	2.736
globatt1	0.098	0.998	0.023	2.002	4.106	0.016
globatt2	0.003	2.356	0.208	0.821	0.117	0.054
globatt3	0.417	0.622	0.464	2.652	2.137	0.001
globID1	0.012	3.518	0.249	1.536	1.745	0.573
globID2	0.115	6.306	0.041	0.803	1.792	0.878
globID3	1.206	1.383	0.000	0.104	5.583	0.929
globID4	2.410	0.138	1.105	2.856	5.531	2.175

Modification Indices for THETA-DELTA

	env4	aut1	aut2	aut3	aut4	qual1
env4	- -					
aut1	4.894	- -				
aut2	0.605	0.160	- -			
aut3	2.108	1.956	0.969	- -		
aut4	1.186	0.823	0.358	0.262	- -	
qual1	0.455	0.223	0.412	1.797	0.425	- -
qual2	0.301	0.544	0.012	1.781	0.073	7.898
qual3	0.497	0.806	1.628	0.725	1.271	0.328
qual4	5.511	2.107	2.461	0.696	0.618	1.249
qual5	1.626	1.465	0.273	0.767	0.136	6.870
qual6	0.434	1.884	0.071	0.003	0.033	0.109
localID1	1.124	0.693	6.161	0.458	0.407	0.006
localID2	2.287	0.018	1.640	0.001	4.135	7.424
localID3	0.131	0.526	0.410	0.112	0.071	0.002
localID4	0.088	0.188	3.777	0.207	0.002	0.000
tn_at	0.134	0.993	0.624	0.051	0.060	2.464
price1	2.082	0.008	0.734	2.458	0.296	1.028
price2	0.097	1.593	0.002	0.740	0.827	0.166

globatt1	5.726	13.690	3.393	0.570	0.294	0.320
globatt2	0.612	2.615	1.047	0.776	0.814	2.913
globatt3	6.021	9.702	0.109	3.027	0.413	1.095
globID1	2.066	0.297	0.061	0.865	4.224	1.494
globID2	0.896	1.404	0.741	4.924	0.158	7.448
globID3	0.073	0.111	0.886	2.686	0.039	1.960
globID4	0.324	0.202	1.910	0.446	0.775	0.089

Modification Indices for THETA-DELTA

	qual2	qual3	qual4	qual5	qual6	localID1
qual2	- -					
qual3	3.215	- -				
qual4	0.546	3.288	- -			
qual5	6.988	23.516	0.146	- -		
qual6	1.716	0.954	5.752	24.225	- -	
localID1	1.267	0.155	1.962	0.908	0.678	- -
localID2	0.009	0.318	0.065	4.113	6.702	1.487
localID3	1.488	0.750	0.031	1.203	3.820	5.381
localID4	8.492	0.055	0.828	8.437	0.395	3.538
tn_at	1.332	0.000	0.002	0.654	0.448	0.011
price1	11.121	0.508	1.287	2.233	3.161	0.052
price2	1.685	0.015	1.808	0.000	0.412	4.204
globatt1	3.588	17.970	0.178	0.320	0.025	2.100
globatt2	0.387	9.966	3.023	3.629	0.099	4.000
globatt3	2.229	0.018	4.595	1.392	0.106	3.462
globID1	1.549	1.346	0.343	3.212	2.320	2.860
globID2	0.602	4.638	0.237	8.168	3.326	0.189
globID3	0.038	5.137	0.192	0.909	0.572	3.699
globID4	2.224	1.999	0.010	0.193	0.886	2.565

Modification Indices for THETA-DELTA

	localID2	localID3	localID4	tn_at	price1	price2

localID2	- -					
localID3	8.110	- -				
localID4	0.026	0.941	- -			
tn_at	0.606	0.667	3.489	- -		
price1	0.777	0.882	0.569	1.331	- -	
price2	0.255	3.959	1.072	1.331	- -	- -
globatt1	0.406	3.523	3.204	2.306	0.126	1.045
globatt2	9.850	11.670	5.184	0.958	0.009	8.274
globatt3	0.086	0.025	3.412	4.360	0.121	0.678
globID1	1.767	0.116	0.901	2.504	1.175	0.694
globID2	4.367	1.347	10.286	0.292	0.022	2.208
globID3	2.577	0.005	1.382	1.853	0.000	0.262
globID4	0.502	0.073	6.887	0.043	4.346	0.486

Modification Indices for THETA-DELTA

	globatt1	globatt2	globatt3	globID1	globID2	globID3
globatt1	- -					
globatt2	0.024	- -				
globatt3	6.605	3.227	- -			
globID1	4.063	1.102	3.347	- -		
globID2	3.671	0.041	10.403	3.073	- -	
globID3	6.956	1.759	0.074	18.359	0.600	- -
globID4	4.062	3.601	0.315	2.268	3.486	1.899

Modification Indices for THETA-DELTA

	globID4
globID4	- -

Expected Change for THETA-DELTA

	cet1	cet2	cet3	cet4	env1	env2
cet1	- -					
cet2	0.270	- -				
cet3	0.039	-0.121	- -			
cet4	-0.135	-0.189	0.125	- -		
env1	-0.058	-0.085	0.073	0.004	- -	
env2	-0.026	0.042	0.013	0.044	0.004	- -
env4	0.002	0.112	-0.047	-0.056	-0.073	0.076
aut1	-0.046	0.105	-0.031	-0.012	0.022	-0.047
aut2	-0.036	0.010	0.063	-0.007	0.032	-0.040
aut3	0.036	0.014	-0.023	-0.047	0.023	-0.033
aut4	0.094	-0.033	-0.059	-0.014	-0.030	0.056
qual1	0.045	0.123	-0.089	-0.042	-0.068	0.006
qual2	-0.069	0.003	0.110	-0.053	-0.001	0.054
qual3	-0.048	-0.122	-0.088	0.238	0.024	-0.066
qual4	0.024	-0.034	0.124	-0.045	0.083	-0.032
qual5	-0.065	-0.092	-0.067	0.111	0.016	-0.034
qual6	0.114	-0.069	-0.077	0.026	-0.003	-0.021
localID1	-0.097	-0.047	0.047	0.101	0.025	0.098
localID2	0.146	0.006	0.017	-0.078	-0.013	-0.063
localID3	-0.042	0.012	-0.045	0.086	0.005	-0.025
localID4	-0.062	-0.003	-0.056	0.042	0.032	-0.047
tn_at	-0.889	1.045	-1.691	1.991	-2.429	2.450
price1	-0.029	0.122	0.008	-0.255	-0.105	0.081
price2	0.040	-0.062	0.103	0.013	0.095	-0.118
globatt1	-0.020	-0.071	-0.009	0.088	0.106	0.008
globatt2	0.005	0.146	0.037	-0.075	-0.024	-0.019
globatt3	-0.039	-0.054	-0.040	0.097	-0.073	-0.002
globID1	-0.007	0.129	0.029	-0.075	-0.067	-0.044
globID2	0.021	-0.178	0.012	0.055	0.069	-0.056
globID3	0.069	-0.084	0.000	0.020	-0.125	0.058
globID4	-0.093	-0.025	-0.061	0.100	0.117	0.084

Expected Change for THETA-DELTA

	env4	aut1	aut2	aut3	aut4	qual1
env4	- -					
aut1	0.116	- -				
aut2	0.032	-0.015	- -			
aut3	-0.062	0.055	0.035	- -		
aut4	-0.051	-0.039	-0.022	-0.020	- -	
qual1	0.032	-0.019	-0.020	-0.043	0.023	- -
qual2	-0.023	0.026	0.003	0.039	-0.009	0.112
qual3	0.044	0.047	-0.052	-0.036	0.052	-0.028
qual4	-0.114	-0.058	0.049	0.027	-0.028	0.046
qual5	0.063	0.049	-0.017	-0.029	0.013	-0.105
qual6	0.041	-0.070	0.011	-0.002	0.008	-0.016
localID1	-0.070	-0.046	-0.106	0.030	0.031	0.004
localID2	0.101	0.008	-0.055	-0.002	0.101	0.136
localID3	-0.018	-0.031	0.021	-0.012	-0.010	0.002
localID4	-0.016	0.019	0.068	-0.017	0.002	0.000
tn_at	0.453	1.010	-0.641	-0.191	0.226	-1.445
price1	0.106	-0.005	-0.040	0.077	0.030	-0.056
price2	-0.024	0.079	-0.002	-0.045	-0.052	0.023
globatt1	-0.157	-0.201	0.078	-0.034	0.026	-0.028
globatt2	-0.069	0.117	-0.058	0.052	-0.059	0.112
globatt3	0.153	0.161	-0.013	-0.074	0.030	-0.049
globID1	0.092	-0.029	0.010	0.040	-0.097	0.058
globID2	0.062	0.064	0.036	-0.098	0.019	-0.134
globID3	-0.018	-0.018	-0.040	0.073	-0.010	0.069
globID4	-0.036	0.023	0.056	-0.028	0.041	0.014

Expected Change for THETA-DELTA

	qual2	qual3	qual4	qual5	qual6	localID1
qual2	- -					
qual3	-0.082	- -				

qual4	0.029	-0.091	- -			
qual5	-0.099	0.244	-0.015	- -		
qual6	-0.058	0.061	-0.118	0.242	- -	
localID1	0.050	-0.026	-0.071	-0.049	0.053	- -
localID2	0.004	-0.037	0.013	-0.105	-0.168	0.098
localID3	-0.042	0.044	-0.007	0.043	0.097	0.153
localID4	-0.106	0.013	0.038	0.122	-0.033	-0.120
tn_at	0.970	0.019	0.038	0.767	-0.791	-0.137
price1	0.165	-0.052	-0.064	-0.085	-0.127	-0.017
price2	-0.067	0.009	0.078	0.000	0.047	0.165
globatt1	-0.084	0.276	-0.021	0.029	0.010	0.099
globatt2	0.037	-0.276	0.118	-0.130	-0.027	0.184
globatt3	0.063	-0.008	-0.103	0.057	0.020	-0.122
globID1	0.054	-0.073	0.029	-0.089	-0.094	0.112
globID2	-0.034	0.140	-0.024	0.146	0.116	-0.030
globID3	-0.009	-0.148	0.022	-0.049	0.049	-0.133
globID4	-0.063	0.088	-0.005	0.022	-0.058	-0.105

Expected Change for THETA-DELTA

	localID2	localID3	localID4	tn_at	price1	price2
localID2	- -					
localID3	-0.188	- -				
localID4	0.010	0.051	- -			
tn_at	-1.027	-0.839	1.984	- -		
price1	0.068	0.055	-0.047	2.407	- -	
price2	0.041	-0.124	-0.068	-3.733	- -	- -
globatt1	-0.044	0.099	-0.100	-2.018	-0.027	-0.083
globatt2	0.291	-0.241	-0.171	-1.665	-0.010	0.304
globatt3	-0.019	-0.008	0.099	2.803	0.025	-0.066
globID1	-0.089	-0.017	-0.052	-2.035	0.080	-0.065
globID2	-0.144	0.061	0.179	0.682	-0.011	0.116
globID3	0.112	0.004	-0.066	1.789	-0.001	-0.041
globID4	0.047	0.014	0.141	-0.250	-0.152	0.052

Expected Change for THETA-DELTA

	globatt1	globatt2	globatt3	globID1	globID2	globID3
globatt1	- -					
globatt2	-0.024	- -				
globatt3	0.664	-0.307	- -			
globID1	0.134	0.093	-0.116	- -		
globID2	-0.130	0.018	0.208	-0.176	- -	
globID3	-0.182	0.122	-0.018	0.691	-0.077	- -
globID4	0.131	-0.166	-0.035	-0.118	0.127	-0.108

Expected Change for THETA-DELTA

	globID4
globID4	- -

Completely Standardized Expected Change for THETA-DELTA

	cet1	cet2	cet3	cet4	env1	env2
cet1	- -					
cet2	0.120	- -				
cet3	0.018	-0.054	- -			
cet4	-0.067	-0.090	0.063	- -		
env1	-0.033	-0.046	0.042	0.003	- -	
env2	-0.013	0.020	0.007	0.024	0.003	- -
env4	0.001	0.055	-0.024	-0.031	-0.046	0.042
aut1	-0.024	0.053	-0.016	-0.007	0.014	-0.027
aut2	-0.021	0.005	0.036	-0.004	0.022	-0.025
aut3	0.020	0.008	-0.013	-0.028	0.016	-0.020
aut4	0.050	-0.017	-0.031	-0.008	-0.019	0.032
qual1	0.023	0.060	-0.046	-0.023	-0.043	0.003

qual2	-0.036	0.001	0.058	-0.030	0.000	0.031
qual3	-0.024	-0.059	-0.045	0.128	0.015	-0.036
qual4	0.012	-0.017	0.066	-0.025	0.053	-0.019
qual5	-0.037	-0.050	-0.039	0.067	0.011	-0.021
qual6	0.060	-0.035	-0.041	0.015	-0.002	-0.012
localID1	-0.044	-0.020	0.022	0.049	0.014	0.049
localID2	0.066	0.003	0.008	-0.038	-0.007	-0.031
localID3	-0.023	0.006	-0.025	0.051	0.003	-0.015
localID4	-0.036	-0.001	-0.032	0.026	0.022	-0.029
tn_at	-0.028	0.032	-0.054	0.067	-0.094	0.084
price1	-0.013	0.052	0.003	-0.122	-0.058	0.039
price2	0.017	-0.025	0.044	0.006	0.049	-0.054
globatt1	-0.010	-0.035	-0.005	0.048	0.066	0.004
globatt2	0.002	0.060	0.016	-0.035	-0.013	-0.009
globatt3	-0.020	-0.027	-0.021	0.054	-0.047	-0.001
globID1	-0.003	0.056	0.013	-0.036	-0.037	-0.022
globID2	0.011	-0.089	0.006	0.031	0.044	-0.032
globID3	0.031	-0.036	0.000	0.010	-0.068	0.028
globID4	-0.055	-0.014	-0.036	0.063	0.085	0.054

Completely Standardized Expected Change for THETA-DELTA

	env4	aut1	aut2	aut3	aut4	qual1
env4	- -					
aut1	0.067	- -				
aut2	0.020	-0.010	- -			
aut3	-0.038	0.035	0.024	- -		
aut4	-0.030	-0.023	-0.015	-0.012	- -	
qual1	0.018	-0.011	-0.012	-0.026	0.013	- -
qual2	-0.013	0.015	0.002	0.024	-0.005	0.064
qual3	0.024	0.027	-0.032	-0.022	0.030	-0.016
qual4	-0.066	-0.035	0.032	0.017	-0.017	0.027
qual5	0.039	0.032	-0.012	-0.020	0.009	-0.066
qual6	0.023	-0.042	0.007	-0.002	0.005	-0.009

localID1	-0.035	-0.024	-0.060	0.017	0.016	0.002
localID2	0.050	0.004	-0.031	-0.001	0.052	0.068
localID3	-0.011	-0.019	0.014	-0.008	-0.006	0.001
localID4	-0.010	0.013	0.048	-0.011	0.001	0.000
tn_at	0.016	0.036	-0.025	-0.007	0.008	-0.050
price1	0.052	-0.003	-0.022	0.042	0.015	-0.027
price2	-0.011	0.038	-0.001	-0.022	-0.025	0.011
globatt1	-0.088	-0.116	0.049	-0.021	0.015	-0.016
globatt2	-0.032	0.057	-0.031	0.027	-0.029	0.053
globatt3	0.088	0.096	-0.009	-0.046	0.018	-0.028
globID1	0.046	-0.015	0.006	0.022	-0.050	0.029
globID2	0.035	0.038	0.023	-0.061	0.011	-0.077
globID3	-0.009	-0.009	-0.022	0.039	-0.005	0.034
globID4	-0.023	0.016	0.041	-0.020	0.027	0.009

Completely Standardized Expected Change for THETA-DELTA

	qual2	qual3	qual4	qual5	qual6	localID1
qual2	- -					
qual3	-0.046	- -				
qual4	0.017	-0.052	- -			
qual5	-0.064	0.150	-0.010	- -		
qual6	-0.034	0.035	-0.070	0.155	- -	
localID1	0.026	-0.013	-0.037	-0.027	0.027	- -
localID2	0.002	-0.018	0.007	-0.058	-0.086	0.044
localID3	-0.026	0.026	-0.004	0.029	0.061	0.083
localID4	-0.068	0.008	0.024	0.085	-0.021	-0.068
tn_at	0.034	0.001	0.001	0.030	-0.028	-0.004
price1	0.083	-0.025	-0.032	-0.047	-0.064	-0.008
price2	-0.032	0.004	0.037	0.000	0.022	0.068
globatt1	-0.048	0.153	-0.012	0.018	0.006	0.050
globatt2	0.018	-0.129	0.057	-0.069	-0.013	0.078
globatt3	0.037	-0.005	-0.061	0.037	0.012	-0.063
globID1	0.027	-0.036	0.015	-0.049	-0.048	0.050

globID2	-0.020	0.079	-0.014	0.093	0.068	-0.015
globID3	-0.004	-0.072	0.011	-0.027	0.024	-0.058
globID4	-0.042	0.056	-0.003	0.016	-0.038	-0.061

Completely Standardized Expected Change for THETA-DELTA

	localID2	localID3	localID4	tn_at	price1	price2
localID2	- -					
localID3	-0.102	- -				
localID4	0.006	0.035	- -			
tn_at	-0.032	-0.032	0.077	- -		
price1	0.030	0.030	-0.026	0.073	- -	
price2	0.017	-0.062	-0.035	-0.106	- -	- -
globatt1	-0.022	0.061	-0.063	-0.070	-0.013	-0.038
globatt2	0.122	-0.124	-0.090	-0.049	-0.004	0.118
globatt3	-0.010	-0.005	0.064	0.100	0.013	-0.031
globID1	-0.040	-0.009	-0.029	-0.063	0.035	-0.027
globID2	-0.073	0.038	0.115	0.024	-0.006	0.055
globID3	0.049	0.002	-0.036	0.054	0.000	-0.017
globID4	0.027	0.010	0.102	-0.010	-0.086	0.028

Completely Standardized Expected Change for THETA-DELTA

	globatt1	globatt2	globatt3	globID1	globID2	globID3
globatt1	- -					
globatt2	-0.011	- -				
globatt3	0.383	-0.149	- -			
globID1	0.067	0.039	-0.060	- -		
globID2	-0.074	0.009	0.123	-0.090	- -	
globID3	-0.089	0.050	-0.009	0.301	-0.038	- -
globID4	0.085	-0.091	-0.023	-0.068	0.084	-0.061

Completely Standardized Expected Change for THETA-DELTA

	globID4
globID4	- -

Maximum Modification Index is 30.73 for Element (31, 2) of LAMBDA-X

!HV Daten aus Studie 1

Standardized Solution

LAMBDA-X

	ETHNO	GREEN	AUTHENTI	QUALITY	LOCALISM	PRICESEN
cet1	1.194	- -	- -	- -	- -	- -
cet2	1.151	- -	- -	- -	- -	- -
cet3	1.211	- -	- -	- -	- -	- -
cet4	1.067	- -	- -	- -	- -	- -
env1	- -	1.007	- -	- -	- -	- -
env2	- -	1.110	- -	- -	- -	- -
env4	- -	0.970	- -	- -	- -	- -
aut1	- -	- -	1.034	- -	- -	- -
aut2	- -	- -	1.054	- -	- -	- -
aut3	- -	- -	1.066	- -	- -	- -
aut4	- -	- -	1.109	- -	- -	- -
qual1	- -	- -	- -	1.154	- -	- -
qual2	- -	- -	- -	1.169	- -	- -
qual3	- -	- -	- -	0.952	- -	- -
qual4	- -	- -	- -	1.087	- -	- -
qual5	- -	- -	- -	0.948	- -	- -
qual6	- -	- -	- -	0.893	- -	- -
localID1	- -	- -	- -	- -	1.155	- -
localID2	- -	- -	- -	- -	1.157	- -
localID3	- -	- -	- -	- -	1.010	- -

localID4	- -	- -	- -	- -	0.898	- -
tn_at	- -	- -	- -	- -	- -	- -
price1	- -	- -	- -	- -	- -	1.015
price2	- -	- -	- -	- -	- -	1.575
globatt1	- -	- -	- -	- -	- -	- -
globatt2	- -	- -	- -	- -	- -	- -
globatt3	- -	- -	- -	- -	- -	- -
globID1	- -	- -	- -	- -	- -	- -
globID2	- -	- -	- -	- -	- -	- -
globID3	- -	- -	- -	- -	- -	- -
globID4	- -	- -	- -	- -	- -	- -

LAMBDA-X

	NUTZEN	GLOBAL	GID
cet1	- -	- -	- -
cet2	- -	- -	- -
cet3	- -	- -	- -
cet4	- -	- -	- -
env1	- -	- -	- -
env2	- -	- -	- -
env4	- -	- -	- -
aut1	- -	- -	- -
aut2	- -	- -	- -
aut3	- -	- -	- -
aut4	- -	- -	- -
qual1	- -	- -	- -
qual2	- -	- -	- -
qual3	- -	- -	- -
qual4	- -	- -	- -
qual5	- -	- -	- -
qual6	- -	- -	- -
localID1	- -	- -	- -
localID2	- -	- -	- -

localID3	- -	- -	- -
localID4	- -	- -	- -
tn_at	21.600	- -	- -
price1	- -	- -	- -
price2	- -	- -	- -
globatt1	- -	0.997	- -
globatt2	- -	0.866	- -
globatt3	- -	1.053	- -
globID1	- -	- -	1.269
globID2	- -	- -	0.874
globID3	- -	- -	1.253
globID4	- -	- -	0.641

PHI

	ETHNO	GREEN	AUTHENTI	QUALITY	LOCALISM	PRICESEN
ETHNO	1.000					
GREEN	0.522	1.000				
AUTHENTI	0.687	0.517	1.000			
QUALITY	0.670	0.514	0.669	1.000		
LOCALISM	0.642	0.414	0.743	0.506	1.000	
PRICESEN	-0.128	-0.169	-0.039	-0.034	0.025	1.000
NUTZEN	0.395	0.211	0.270	0.292	0.176	-0.312
GLOBAL	-0.064	0.088	0.187	0.062	0.080	0.183
GID	-0.022	0.286	0.025	-0.010	0.016	-0.009

PHI

	NUTZEN	GLOBAL	GID
NUTZEN	1.000		
GLOBAL	-0.078	1.000	
GID	0.064	0.261	1.000

!HV Daten aus Studie 1

Completely Standardized Solution

LAMBDA-X

	ETHNO	GREEN	AUTHENTI	QUALITY	LOCALISM	PRICESEN
cet1	0.814	- -	- -	- -	- -	- -
cet2	0.752	- -	- -	- -	- -	- -
cet3	0.833	- -	- -	- -	- -	- -
cet4	0.775	- -	- -	- -	- -	- -
env1	- -	0.841	- -	- -	- -	- -
env2	- -	0.823	- -	- -	- -	- -
env4	- -	0.725	- -	- -	- -	- -
aut1	- -	- -	0.801	- -	- -	- -
aut2	- -	- -	0.884	- -	- -	- -
aut3	- -	- -	0.874	- -	- -	- -
aut4	- -	- -	0.858	- -	- -	- -
qual1	- -	- -	- -	0.864	- -	- -
qual2	- -	- -	- -	0.897	- -	- -
qual3	- -	- -	- -	0.704	- -	- -
qual4	- -	- -	- -	0.838	- -	- -
qual5	- -	- -	- -	0.790	- -	- -
qual6	- -	- -	- -	0.686	- -	- -
localID1	- -	- -	- -	- -	0.772	- -
localID2	- -	- -	- -	- -	0.769	- -
localID3	- -	- -	- -	- -	0.823	- -
localID4	- -	- -	- -	- -	0.753	- -
tn_at	- -	- -	- -	- -	- -	- -
price1	- -	- -	- -	- -	- -	0.668
price2	- -	- -	- -	- -	- -	0.970
globatt1	- -	- -	- -	- -	- -	- -
globatt2	- -	- -	- -	- -	- -	- -
globatt3	- -	- -	- -	- -	- -	- -

globID1	- -	- -	- -	- -	- -	- -
globID2	- -	- -	- -	- -	- -	- -
globID3	- -	- -	- -	- -	- -	- -
globID4	- -	- -	- -	- -	- -	- -

LAMBDA-X

	NUTZEN	GLOBAL	GID
cet1	- -	- -	- -
cet2	- -	- -	- -
cet3	- -	- -	- -
cet4	- -	- -	- -
env1	- -	- -	- -
env2	- -	- -	- -
env4	- -	- -	- -
aut1	- -	- -	- -
aut2	- -	- -	- -
aut3	- -	- -	- -
aut4	- -	- -	- -
qual1	- -	- -	- -
qual2	- -	- -	- -
qual3	- -	- -	- -
qual4	- -	- -	- -
qual5	- -	- -	- -
qual6	- -	- -	- -
localID1	- -	- -	- -
localID2	- -	- -	- -
localID3	- -	- -	- -
localID4	- -	- -	- -
tn_at	1.000	- -	- -
price1	- -	- -	- -
price2	- -	- -	- -
globatt1	- -	0.747	- -
globatt2	- -	0.547	- -

globatt3	- -	0.810	- -
globID1	- -	- -	0.845
globID2	- -	- -	0.669
globID3	- -	- -	0.820
globID4	- -	- -	0.555

PHI

	ETHNO	GREEN	AUTHENTI	QUALITY	LOCALISM	PRICESEN
ETHNO	1.000					
GREEN	0.522	1.000				
AUTHENTI	0.687	0.517	1.000			
QUALITY	0.670	0.514	0.669	1.000		
LOCALISM	0.642	0.414	0.743	0.506	1.000	
PRICESEN	-0.128	-0.169	-0.039	-0.034	0.025	1.000
NUTZEN	0.395	0.211	0.270	0.292	0.176	-0.312
GLOBAL	-0.064	0.088	0.187	0.062	0.080	0.183
GID	-0.022	0.286	0.025	-0.010	0.016	-0.009

PHI

	NUTZEN	GLOBAL	GID
NUTZEN	1.000		
GLOBAL	-0.078	1.000	
GID	0.064	0.261	1.000

THETA-DELTA

cet1	cet2	cet3	cet4	env1	env2
0.337	0.434	0.307	0.399	0.293	0.322

THETA-DELTA

env4	aut1	aut2	aut3	aut4	qual1
0.474	0.358	0.219	0.236	0.265	0.253

THETA-DELTA

qual2	qual3	qual4	qual5	qual6	localID1
0.195	0.504	0.297	0.375	0.529	0.404

THETA-DELTA

localID2	localID3	localID4	tn_at	price1	price2
0.409	0.323	0.434	- -	0.554	0.060

THETA-DELTA

globatt1	globatt2	globatt3	globID1	globID2	globID3
0.442	0.701	0.344	0.286	0.553	0.328

THETA-DELTA

globID4
0.692

W_A_R_N_I_N_G: THETA-DELTA is not positive definite

Time used 0.938 seconds

2 Apples Multiitem for Evaluation of Reliability and Validity incl Fit Statistics

DATE: 9/15/2021
TIME: 8:58

L I S R E L 9.30 (64 Bit)

BY

Karl G. Jˆreskog & Dag Sˆrbom

This program is published exclusively by
Scientific Software International, Inc.
http://www.ssicentral.com

Copyright by Scientific Software International, Inc., 1981-2017
Use of this program is subject to the terms specified in the
Universal Copyright Convention.

!LEGEND for the expression used in this file:
cet-Consumerethnocentrism item, ETHNO-Consumerethnocentrism construct
env-Green Consumer Value item, GREEN-Green Consumer Value construct
aut-Authenticity item, AUTHENTI-Authenticity construct
qual-Healthiness Bias item, QUALITY-Healthiness Bias construct
localID-Local Identity item, LOCALISM-Local Identity construct
tn_at-Part worth utility of the local choice
price-Price Consciousness item, PRICESENS-Price Consciousness/Sensibility construct
globatt-Global Attitude item, GLOBAL-Global Attitude construct
globID-Global Identity item, GID-Global Identity construct

The following lines were read from file C:\Users\Petra Riefler\Desktop\Tomatoes_Measurement_Model_Multiitem.spl:

```
 !Data
 !Tomaten
 !Model 1, 15.09.21
 Observed Variables: cet1 cet2 cet3 cet4 env1 env2 env3 env4 env5 aut1 aut2 aut3 aut4 qual1 qual2 qual3
qual4 qual5 qual6 localID1 localID2 localID3 localID4 tn_at price1 price2 globatt1 globatt2 globatt3
globID1 globID2 globID3 globID4
 Covariance Matrix:
 2.029
 1.519 2.291
 1.604 1.531 2.411
 1.235 1.148 1.493 2.085
 0.721 0.634 0.784 0.772 1.373
 0.821 0.846 0.947 0.943 1.071 1.808
 0.253 0.076 0.394 0.438 0.351 0.255 2.985
 0.899 0.906 0.982 0.778 1.073 1.193 0.558 2.110
 0.529 0.571 0.837 0.762 0.711 0.769 1.011 0.857 3.254
 0.819 0.670 0.910 0.665 0.573 0.638 0.041 0.611 0.230 1.387
 0.750 0.655 0.867 0.660 0.546 0.699 0.080 0.651 0.293 0.936 1.228
 0.809 0.797 0.973 0.787 0.633 0.694 0.090 0.769 0.334 1.107 1.051 1.554
 0.866 0.756 0.971 0.753 0.525 0.594 0.253 0.634 0.227 1.054 0.969 1.070 1.756
 0.662 0.720 0.725 0.538 0.384 0.601 0.130 0.553 0.082 0.702 0.608 0.696 0.712 1.766
 0.806 0.932 0.900 0.662 0.361 0.661 -0.048 0.481 0.085 0.755 0.695 0.801 0.764 1.273 1.693
 0.798 0.834 0.948 0.791 0.534 0.783 0.107 0.606 0.380 0.629 0.596 0.726 0.628 0.810 0.789 1.536
 0.813 0.751 0.924 0.712 0.490 0.617 -0.050 0.591 0.077 0.751 0.774 0.965 0.889 1.007 1.030 0.827 1.627
 0.791 0.686 0.751 0.758 0.385 0.595 0.083 0.582 0.058 0.709 0.697 0.858 0.700 0.984 1.044 0.874 1.032
1.606
 0.846 0.757 0.847 0.784 0.555 0.635 0.130 0.611 0.147 0.697 0.671 0.883 0.720 0.834 0.881 0.935 0.985
1.167 1.619
 0.614 0.752 0.628 0.545 0.338 0.387 0.074 0.444 -0.037 0.655 0.543 0.766 0.883 0.575 0.672 0.621 0.653
0.674 0.662 2.002
 0.764 0.860 0.926 0.689 0.401 0.464 0.094 0.483 -0.135 0.776 0.670 0.781 0.981 0.715 0.802 0.620 0.681
0.628 0.684 1.263 2.211
 0.684 0.525 0.695 0.630 0.505 0.556 0.292 0.573 -0.006 0.608 0.568 0.743 0.832 0.372 0.541 0.659 0.695
0.626 0.561 1.268 1.066 1.918
 0.615 0.661 0.703 0.745 0.483 0.558 0.188 0.498 0.349 0.613 0.654 0.851 0.758 0.338 0.556 0.570 0.686
```

0.676 0.620 1.085 0.924 1.049 1.637
12.971 12.573 14.836 17.888 7.265 8.787 7.682 8.861 11.608 6.075 6.511 7.273 7.733 4.406 6.589 8.599
6.471 6.849 6.089 2.490 6.764 6.007 6.564 453.020
−0.177 0.065 −0.238 −0.464 −0.308 −0.287 −0.535 −0.231 −0.868 0.089 0.013 0.076 0.077 −0.032 0.150 0.029
0.060 0.090 0.069 0.500 0.275 0.353 0.111 −9.882 2.504
−0.415 −0.292 −0.559 −0.697 −0.454 −0.398 −0.782 −0.439 −1.125 −0.124 −0.200 −0.201 −0.268 0.021 0.001
−0.161 −0.129 −0.025 −0.172 0.193 0.206 0.139 −0.137 −14.345 1.810 3.022
−0.131 −0.074 −0.055 0.006 0.001 0.187 −0.214 0.036 −0.185 −0.019 0.009 0.006 −0.011 −0.152 −0.143 0.113
−0.030 0.027 −0.025 0.093 −0.055 0.048 0.268 −0.868 0.335 0.319 2.155
−0.138 −0.128 −0.203 −0.223 −0.129 0.051 −0.451 −0.253 −0.962 0.223 0.132 0.051 0.196 0.273 0.131 0.040
0.081 0.018 0.093 0.349 0.400 0.132 0.059 −4.642 0.770 0.813 1.301 2.731
0.030 −0.074 0.101 0.096 0.167 0.300 −0.118 0.155 −0.122 0.093 0.079 0.097 0.113 −0.012 0.011 0.196
0.131 0.015 0.117 0.153 0.075 0.263 0.269 −0.061 0.381 0.326 1.369 1.040 1.775
0.391 0.425 0.537 0.430 0.533 0.727 0.324 0.642 0.661 0.306 0.330 0.462 0.299 0.298 0.339 0.355 0.183
0.321 0.331 0.128 0.349 0.279 0.361 4.961 −0.198 −0.327 −0.066 −0.209 −0.067 2.295
0.163 0.085 0.124 0.095 0.095 0.120 0.062 0.071 −0.008 0.042 0.044 0.034 0.067 0.067 0.082 0.094 0.068
0.030 0.075 0.012 0.060 0.043 0.020 0.839 0.055 0.070 0.076 0.112 0.118 0.120 0.250
0.127 0.215 0.161 0.065 0.091 −0.004 −0.082 −0.038 0.215 0.245 0.136 0.171 0.077 −0.036 0.089 0.059
0.041 0.125 0.146 −0.038 0.058 −0.024 0.063 2.070 −0.011 −0.197 −0.274 −0.352 −0.235 0.289 −0.053 1.072
−0.174 −0.180 −0.250 0.003 0.065 0.078 0.384 0.121 0.370 −0.166 −0.097 −0.172 −0.245 −0.206 −0.242
−0.117 −0.245 −0.058 −0.145 −0.126 −0.308 −0.055 −0.002 −0.188 −0.101 −0.327 0.107 −0.043 0.095 0.251
0.003 −0.066 1.359
−0.012 −0.036 −0.046 −0.064 −0.039 −0.051 −0.094 −0.009 −0.003 −0.041 −0.021 0.018 0.041 0.048 0.096
0.003 0.079 −0.030 −0.094 0.075 0.175 0.114 0.046 0.280 −0.003 0.082 −0.128 −0.019 0.035 −0.013 0.020
−0.047 −0.117 0.672
0.033 0.034 0.027 −0.018 0.442 0.590 0.018 0.358 0.199 −0.027 0.033 −0.040 −0.211 0.110 0.133 0.153
0.058 0.103 0.104 −0.005 −0.210 0.135 0.169 −1.828 0.141 0.230 0.318 0.430 0.278 0.015 0.007 −0.367 0.249
−0.144 2.720
0.161 0.169 0.253 0.261 0.357 0.480 0.185 0.499 0.108 0.290 0.293 0.316 0.244 0.231 0.198 0.243 0.272
0.290 0.337 0.171 −0.020 0.285 0.295 0.207 0.216 0.096 0.285 0.313 0.397 0.149 0.035 −0.214 0.222 −0.120
1.242 1.871
0.081 0.007 0.122 0.126 0.369 0.509 0.065 0.222 0.116 0.009 0.094 0.057 −0.018 0.263 0.134 0.142 0.017
0.201 0.138 0.056 −0.181 0.123 0.024 −0.785 −0.025 0.002 0.267 0.430 0.250 0.060 −0.002 −0.290 0.282
−0.143 2.133 1.157 2.700
0.191 0.128 0.267 0.285 0.337 0.466 0.101 0.332 0.168 0.294 0.349 0.421 0.219 0.262 0.349 0.341 0.346

```
0.401 0.348 0.236 0.122 0.401 0.546 3.070 -0.115 -0.158 0.254 0.154 0.293 0.180 -0.036 -0.080 0.111
-0.095 1.011 0.719 0.923 1.646
 Sample Size: 253
 Latent Variables: ETHNO GREEN AUTHENTICITY QUALITY LOCALISM PRICESENS NUTZEN GLOBAL GID
 Relationships:
 cet1=1*ETHNO
 cet2 cet3 cet4 = ETHNO
 env1 = 1*GREEN
 env2 env4 = GREEN
 aut1 = 1*AUTHENTICITY
 aut2 aut3 aut4 = AUTHENTICITY
 qual1 = 1*QUALITY
 qual2 qual3 qual4 qual5 qual6 = QUALITY
 tn_at=1*NUTZEN
 set the error variance of tn_at equal to 0
 price1=1*PRICESENS
 price2 = PRICESENS
 localID1 = 1*LOCALISM
 localID2 localID3 localID4 = LOCALISM
 globatt1 = 1*GLOBAL
 globatt2 globatt3 =GLOBAL
 globID1=1*GID
 globID2 globID3 globID4 =GID
 Options: ND=3 SC RS MI
 Path Diagram
 End of Problem

 Sample Size =   253

 !HV Daten aus Studie 1

         Covariance Matrix

               cet1       cet2       cet3       cet4       env1       env2
            --------   --------   --------   --------   --------   --------
```

cet1	2.029					
cet2	1.519	2.291				
cet3	1.604	1.531	2.411			
cet4	1.235	1.148	1.493	2.085		
env1	0.721	0.634	0.784	0.772	1.373	
env2	0.821	0.846	0.947	0.943	1.071	1.808
env4	0.899	0.906	0.982	0.778	1.073	1.193
aut1	0.819	0.670	0.910	0.665	0.573	0.638
aut2	0.750	0.655	0.867	0.660	0.546	0.699
aut3	0.809	0.797	0.973	0.787	0.633	0.694
aut4	0.866	0.756	0.971	0.753	0.525	0.594
qual1	0.662	0.720	0.725	0.538	0.384	0.601
qual2	0.806	0.932	0.900	0.662	0.361	0.661
qual3	0.798	0.834	0.948	0.791	0.534	0.783
qual4	0.813	0.751	0.924	0.712	0.490	0.617
qual5	0.791	0.686	0.751	0.758	0.385	0.595
qual6	0.846	0.757	0.847	0.784	0.555	0.635
localID1	0.614	0.752	0.628	0.545	0.338	0.387
localID2	0.764	0.860	0.926	0.689	0.401	0.464
localID3	0.684	0.525	0.695	0.630	0.505	0.556
localID4	0.615	0.661	0.703	0.745	0.483	0.558
tn_at	12.971	12.573	14.836	17.888	7.265	8.787
price1	-0.177	0.065	-0.238	-0.464	-0.308	-0.287
price2	-0.415	-0.292	-0.559	-0.697	-0.454	-0.398
globatt1	-0.131	-0.074	-0.055	0.006	0.001	0.187
globatt2	-0.138	-0.128	-0.203	-0.223	-0.129	0.051
globatt3	0.030	-0.074	0.101	0.096	0.167	0.300
globID1	0.033	0.034	0.027	-0.018	0.442	0.590
globID2	0.161	0.169	0.253	0.261	0.357	0.480
globID3	0.081	0.007	0.122	0.126	0.369	0.509
globID4	0.191	0.128	0.267	0.285	0.337	0.466

Covariance Matrix

	env4	aut1	aut2	aut3	aut4	qual1

env4	2.110					
aut1	0.611	1.387				
aut2	0.651	0.936	1.228			
aut3	0.769	1.107	1.051	1.554		
aut4	0.634	1.054	0.969	1.070	1.756	
qual1	0.553	0.702	0.608	0.696	0.712	1.766
qual2	0.481	0.755	0.695	0.801	0.764	1.273
qual3	0.606	0.629	0.596	0.726	0.628	0.810
qual4	0.591	0.751	0.774	0.965	0.889	1.007
qual5	0.582	0.709	0.697	0.858	0.700	0.984
qual6	0.611	0.697	0.671	0.883	0.720	0.834
localID1	0.444	0.655	0.543	0.766	0.883	0.575
localID2	0.483	0.776	0.670	0.781	0.981	0.715
localID3	0.573	0.608	0.568	0.743	0.832	0.372
localID4	0.498	0.613	0.654	0.851	0.758	0.338
tn_at	8.861	6.075	6.511	7.273	7.733	4.406
price1	-0.231	0.089	0.013	0.076	0.077	-0.032
price2	-0.439	-0.124	-0.200	-0.201	-0.268	0.021
globatt1	0.036	-0.019	0.009	0.006	-0.011	-0.152
globatt2	-0.253	0.223	0.132	0.051	0.196	0.273
globatt3	0.155	0.093	0.079	0.097	0.113	-0.012
globID1	0.358	-0.027	0.033	-0.040	-0.211	0.110
globID2	0.499	0.290	0.293	0.316	0.244	0.231
globID3	0.222	0.009	0.094	0.057	-0.018	0.263
globID4	0.332	0.294	0.349	0.421	0.219	0.262

Covariance Matrix

	qual2	qual3	qual4	qual5	qual6	localID1
qual2	1.693					
qual3	0.789	1.536				
qual4	1.030	0.827	1.627			
qual5	1.044	0.874	1.032	1.606		

qual6	0.881	0.935	0.985	1.167	1.619	
localID1	0.672	0.621	0.653	0.674	0.662	2.002
localID2	0.802	0.620	0.681	0.628	0.684	1.263
localID3	0.541	0.659	0.695	0.626	0.561	1.268
localID4	0.556	0.570	0.686	0.676	0.620	1.085
tn_at	6.589	8.599	6.471	6.849	6.089	2.490
price1	0.150	0.029	0.060	0.090	0.069	0.500
price2	0.001	−0.161	−0.129	−0.025	−0.172	0.193
globatt1	−0.143	0.113	−0.030	0.027	−0.025	0.093
globatt2	0.131	0.040	0.081	0.018	0.093	0.349
globatt3	0.011	0.196	0.131	0.015	0.117	0.153
globID1	0.133	0.153	0.058	0.103	0.104	−0.005
globID2	0.198	0.243	0.272	0.290	0.337	0.171
globID3	0.134	0.142	0.017	0.201	0.138	0.056
globID4	0.349	0.341	0.346	0.401	0.348	0.236

Covariance Matrix

	localID2	localID3	localID4	tn_at	price1	price2
localID2	2.211					
localID3	1.066	1.918				
localID4	0.924	1.049	1.637			
tn_at	6.764	6.007	6.564	453.020		
price1	0.275	0.353	0.111	−9.882	2.504	
price2	0.206	0.139	−0.137	−14.345	1.810	3.022
globatt1	−0.055	0.048	0.268	−0.868	0.335	0.319
globatt2	0.400	0.132	0.059	−4.642	0.770	0.813
globatt3	0.075	0.263	0.269	−0.061	0.381	0.326
globID1	−0.210	0.135	0.169	−1.828	0.141	0.230
globID2	−0.020	0.285	0.295	0.207	0.216	0.096
globID3	−0.181	0.123	0.024	−0.785	−0.025	0.002
globID4	0.122	0.401	0.546	3.070	−0.115	−0.158

Covariance Matrix

	globatt1	globatt2	globatt3	globID1	globID2	globID3
	--------	--------	--------	--------	--------	--------
globatt1	2.155					
globatt2	1.301	2.731				
globatt3	1.369	1.040	1.775			
globID1	0.318	0.430	0.278	2.720		
globID2	0.285	0.313	0.397	1.242	1.871	
globID3	0.267	0.430	0.250	2.133	1.157	2.700
globID4	0.254	0.154	0.293	1.011	0.719	0.923

Covariance Matrix

	globID4
	--------
globID4	1.646

Total Variance = 511.791 Generalized Variance = 347.202

Largest Eigenvalue = 457.536 Smallest Eigenvalue = 0.232

Condition Number = 44.436

WARNING: The Condition Number indicates severe multicollinearity.

One or more variables may be redundant.

!HV Daten aus Studie 1

Number of Iterations = 15

LISREL Estimates (Maximum Likelihood)

```
         Measurement Equations


    cet1 = 1.000*ETHNO, Errorvar.= 0.585  , R≤ = 0.712
Standerr                         (0.0705)
Z-values                          8.295
P-values                          0.000

    cet2 = 0.968*ETHNO, Errorvar.= 0.939  , R≤ = 0.590
Standerr  (0.0690)               (0.0984)
Z-values   14.027                 9.539
P-values   0.000                  0.000

    cet3 = 1.103*ETHNO, Errorvar.= 0.653  , R≤ = 0.729
Standerr  (0.0675)               (0.0813)
Z-values   16.348                 8.033
P-values   0.000                  0.000

    cet4 = 0.914*ETHNO, Errorvar.= 0.878  , R≤ = 0.579
Standerr  (0.0661)               (0.0913)
Z-values   13.830                 9.619
P-values   0.000                  0.000

    env1 = 1.000*GREEN, Errorvar.= 0.444  , R≤ = 0.677
Standerr                         (0.0590)
Z-values                          7.527
P-values                          0.000

    env2 = 1.160*GREEN, Errorvar.= 0.559  , R≤ = 0.691
Standerr  (0.0833)               (0.0767)
Z-values   13.913                 7.282
P-values   0.000                  0.000

    env4 = 1.124*GREEN, Errorvar.= 0.935 , R≤ = 0.557
Standerr  (0.0906)               (0.103)
```

```
Z-values   12.406                  9.055
P-values   0.000                   0.000

    aut1 = 1.000*AUTHENTI, Errorvar.= 0.389  , R≤ = 0.719
Standerr                           (0.0446)
Z-values                            8.737
P-values                            0.000

    aut2 = 0.944*AUTHENTI, Errorvar.= 0.339  , R≤ = 0.724
Standerr  (0.0560)                 (0.0391)
Z-values   16.871                   8.676
P-values   0.000                    0.000

    aut3 = 1.103*AUTHENTI, Errorvar.= 0.340  , R≤ = 0.781
Standerr  (0.0615)                 (0.0439)
Z-values   17.923                   7.756
P-values   0.000                    0.000

    aut4 = 1.024*AUTHENTI, Errorvar.= 0.710  , R≤ = 0.596
Standerr  (0.0707)                 (0.0723)
Z-values   14.486                   9.815
P-values   0.000                    0.000

   qual1 = 1.000*QUALITY, Errorvar.= 0.809  , R≤ = 0.542
Standerr                          (0.0814)
Z-values                           9.939
P-values                           0.000

   qual2 = 1.043*QUALITY, Errorvar.= 0.652  , R≤ = 0.615
Standerr  (0.0848)                (0.0687)
Z-values   12.310                  9.480
P-values   0.000                   0.000

   qual3 = 0.875*QUALITY, Errorvar.= 0.804  , R≤ = 0.476
Standerr  (0.0813)                (0.0785)
```

```
Z-values   10.759                   10.237
P-values   0.000                    0.000

   qual4 = 1.034*QUALITY, Errorvar.= 0.605  , R≤ = 0.628
Standerr  (0.0830)                 (0.0645)
Z-values   12.448                   9.379
P-values   0.000                    0.000

   qual5 = 1.067*QUALITY, Errorvar.= 0.517  , R≤ = 0.678
Standerr  (0.0823)                 (0.0580)
Z-values   12.954                   8.921
P-values   0.000                    0.000

   qual6 = 1.012*QUALITY, Errorvar.= 0.640  , R≤ = 0.605
Standerr  (0.0829)                 (0.0670)
Z-values   12.202                   9.554
P-values   0.000                    0.000

localID1 = 1.000*LOCALISM, Errorvar.= 0.674  , R≤ = 0.664
Standerr                             (0.0869)
Z-values                              7.752
P-values                              0.000

localID2 = 0.916*LOCALISM, Errorvar.= 1.096 , R≤ = 0.504
Standerr  (0.0793)                   (0.116)
Z-values   11.556                     9.452
P-values   0.000                      0.000

localID3 = 0.928*LOCALISM, Errorvar.= 0.774  , R≤ = 0.596
Standerr  (0.0729)                   (0.0898)
Z-values   12.724                     8.624
P-values   0.000                      0.000

localID4 = 0.826*LOCALISM, Errorvar.= 0.730  , R≤ = 0.554
Standerr  (0.0677)                   (0.0807)
```

```
Z-values   12.206                     9.047
P-values   0.000                      0.000

   tn_at = 1.000*NUTZEN,, R≤ = 1.000

  price1 = 1.000*PRICESEN, Errorvar.= 0.930 , R≤ = 0.629
Standerr                             (0.179)
Z-values                              5.197
P-values                              0.000

  price2 = 1.150*PRICESEN, Errorvar.= 0.941 , R≤ = 0.689
Standerr  (0.132)                    (0.226)
Z-values   8.697                      4.168
P-values   0.000                      0.000

globatt1 = 1.000*GLOBAL, Errorvar.= 0.559 , R≤ = 0.740
Standerr                           (0.129)
Z-values                            4.350
P-values                            0.000

globatt2 = 0.809*GLOBAL, Errorvar.= 1.687 , R≤ = 0.382
Standerr  (0.0870)                 (0.171)
Z-values   9.298                    9.847
P-values   0.000                    0.000

globatt3 = 0.851*GLOBAL, Errorvar.= 0.621 , R≤ = 0.650
Standerr  (0.0776)                 (0.102)
Z-values   10.957                   6.065
P-values   0.000                    0.000

 globID1 = 1.000*GID, Errorvar.= 0.398 , R≤ = 0.854
Standerr                        (0.113)
Z-values                         3.531
P-values                         0.000
```

```
 globID2 = 0.548*GID, Errorvar.= 1.173 , R≤ = 0.373
Standerr  (0.0525)             (0.112)
Z-values   10.444               10.453
P-values   0.000                0.000

 globID3 = 0.912*GID, Errorvar.= 0.767 , R≤ = 0.716
Standerr  (0.0587)             (0.113)
Z-values   15.534               6.767
P-values   0.000                0.000

 globID4 = 0.445*GID, Errorvar.= 1.186 , R≤ = 0.279
Standerr  (0.0509)             (0.110)
Z-values   8.741                10.739
P-values   0.000                0.000
```

Covariance Matrix of Independent Variables

```
              ETHNO      GREEN   AUTHENTI    QUALITY   LOCALISM   PRICESEN
           --------   --------   --------   --------   --------   --------
   ETHNO      1.444
            (0.179)
              8.063

   GREEN      0.755      0.929
            (0.105)    (0.123)
              7.208      7.524

AUTHENTI      0.785      0.570      0.998
            (0.105)    (0.084)    (0.122)
              7.448      6.819      8.203

 QUALITY      0.770      0.495      0.721      0.957
            (0.109)    (0.082)    (0.095)    (0.145)
              7.058      6.012      7.581      6.617
```

LOCALISM	0.729	0.463	0.752	0.665	1.328	
	(0.117)	(0.091)	(0.104)	(0.103)	(0.179)	
	6.213	5.071	7.262	6.485	7.422	
PRICESEN	-0.327	-0.308	-0.066	-0.007	0.207	1.574
	(0.117)	(0.097)	(0.093)	(0.091)	(0.112)	(0.260)
	-2.792	-3.160	-0.708	-0.079	1.845	6.047
NUTZEN	14.273	7.529	6.680	6.384	5.580	-11.352
	(1.996)	(1.509)	(1.482)	(1.482)	(1.724)	(2.143)
	7.150	4.988	4.507	4.306	3.236	-5.296
GLOBAL	-0.044	0.108	0.056	0.024	0.179	0.401
	(0.110)	(0.091)	(0.091)	(0.089)	(0.109)	(0.128)
	-0.396	1.184	0.616	0.266	1.641	3.133
GID	0.084	0.461	0.052	0.171	0.090	0.111
	(0.129)	(0.111)	(0.106)	(0.105)	(0.126)	(0.142)
	0.651	4.136	0.493	1.624	0.716	0.778

Covariance Matrix of Independent Variables

	NUTZEN	GLOBAL	GID
NUTZEN	453.020		
	(40.278)		
	11.247		
GLOBAL	-1.156	1.596	
	(1.844)	(0.220)	
	-0.627	7.265	
GID	-1.064	0.375	2.322

```
            (2.147)    (0.142)    (0.262)
             -0.496      2.644      8.865


                          Log-likelihood Values

                         Estimated Model          Saturated Model
                         ---------------          ---------------
 Number of free parameters(t)        97                      496
 -2ln(L)                      10072.933                 9323.026
 AIC (Akaike, 1974)*          10266.933                10315.026
 BIC (Schwarz, 1978)*         10609.671                12067.588

*LISREL uses AIC= 2t - 2ln(L) and BIC = tln(N)- 2ln(L)


                        Goodness-of-Fit Statistics

 Degrees of Freedom for (C1)-(C2)                       399
 Maximum Likelihood Ratio Chi-Square (C1)               749.906 (P = 0.0000)
 Browne's (1984) ADF Chi-Square (C2_NT)                 773.629 (P = 0.0000)

 Estimated Non-centrality Parameter (NCP)               350.906
 90 Percent Confidence Interval for NCP                 (277.599 ; 432.023)

 Minimum Fit Function Value                             2.964
 Population Discrepancy Function Value (F0)             1.387
 90 Percent Confidence Interval for F0                  (1.097 ; 1.708)
 Root Mean Square Error of Approximation (RMSEA)        0.0590
 90 Percent Confidence Interval for RMSEA               (0.0524 ; 0.0654)
 P-Value for Test of Close Fit (RMSEA < 0.05)           0.0126

 Expected Cross-Validation Index (ECVI)                 3.731
 90 Percent Confidence Interval for ECVI                (3.441 ; 4.051)
 ECVI for Saturated Model                               3.921
```

ECVI for Independence Model 19.942

Chi-Square for Independence Model (465 df) 4983.312

Normed Fit Index (NFI)	0.850
Non-Normed Fit Index (NNFI)	0.909
Parsimony Normed Fit Index (PNFI)	0.729
Comparative Fit Index (CFI)	0.922
Incremental Fit Index (IFI)	0.923
Relative Fit Index (RFI)	0.825

Critical N (CN) 158.148

Root Mean Square Residual (RMR)	0.445
Standardized RMR	0.0637
Goodness of Fit Index (GFI)	0.835
Adjusted Goodness of Fit Index (AGFI)	0.795
Parsimony Goodness of Fit Index (PGFI)	0.672

!HV Daten aus Studie 1

Fitted Covariance Matrix

	cet1	cet2	cet3	cet4	env1	env2
cet1	2.029					
cet2	1.398	2.291				
cet3	1.593	1.542	2.411			
cet4	1.320	1.277	1.456	2.085		
env1	0.755	0.731	0.833	0.690	1.373	
env2	0.875	0.847	0.966	0.800	1.077	1.808
env4	0.849	0.821	0.937	0.776	1.045	1.212
aut1	0.785	0.759	0.866	0.717	0.570	0.661
aut2	0.741	0.717	0.817	0.677	0.538	0.624

aut3	0.866	0.838	0.955	0.791	0.629	0.729
aut4	0.804	0.778	0.887	0.735	0.584	0.677
qual1	0.770	0.745	0.849	0.704	0.495	0.573
qual2	0.803	0.777	0.886	0.734	0.516	0.598
qual3	0.673	0.651	0.743	0.615	0.433	0.502
qual4	0.796	0.770	0.878	0.727	0.511	0.593
qual5	0.821	0.795	0.906	0.751	0.528	0.612
qual6	0.779	0.754	0.859	0.712	0.500	0.580
localID1	0.729	0.705	0.804	0.666	0.463	0.537
localID2	0.668	0.646	0.736	0.610	0.424	0.492
localID3	0.676	0.654	0.746	0.618	0.429	0.498
localID4	0.602	0.583	0.664	0.550	0.382	0.443
tn_at	14.273	13.810	15.746	13.045	7.529	8.730
price1	-0.327	-0.316	-0.361	-0.299	-0.308	-0.357
price2	-0.376	-0.364	-0.415	-0.344	-0.354	-0.410
globatt1	-0.044	-0.042	-0.048	-0.040	0.108	0.125
globatt2	-0.035	-0.034	-0.039	-0.032	0.087	0.101
globatt3	-0.037	-0.036	-0.041	-0.034	0.092	0.106
globID1	0.084	0.081	0.092	0.077	0.461	0.535
globID2	0.046	0.044	0.051	0.042	0.253	0.293
globID3	0.076	0.074	0.084	0.070	0.421	0.488
globID4	0.037	0.036	0.041	0.034	0.205	0.238

Fitted Covariance Matrix

	env4	aut1	aut2	aut3	aut4	qual1
env4	2.110					
aut1	0.641	1.387				
aut2	0.605	0.942	1.228			
aut3	0.707	1.100	1.039	1.554		
aut4	0.656	1.022	0.964	1.127	1.756	
qual1	0.556	0.721	0.681	0.795	0.738	1.766
qual2	0.580	0.752	0.710	0.830	0.770	0.998
qual3	0.486	0.631	0.595	0.696	0.646	0.837

qual4	0.575	0.745	0.704	0.822	0.763	0.989
qual5	0.593	0.769	0.726	0.849	0.788	1.021
qual6	0.563	0.730	0.689	0.805	0.747	0.968
localID1	0.520	0.752	0.710	0.830	0.770	0.665
localID2	0.477	0.689	0.651	0.760	0.706	0.609
localID3	0.483	0.698	0.659	0.770	0.715	0.617
localID4	0.430	0.622	0.587	0.686	0.637	0.550
tn_at	8.465	6.680	6.306	7.367	6.840	6.384
price1	-0.346	-0.066	-0.062	-0.072	-0.067	-0.007
price2	-0.398	-0.075	-0.071	-0.083	-0.077	-0.008
globatt1	0.121	0.056	0.053	0.062	0.057	0.024
globatt2	0.098	0.045	0.043	0.050	0.046	0.019
globatt3	0.103	0.048	0.045	0.052	0.049	0.020
globID1	0.518	0.052	0.049	0.057	0.053	0.171
globID2	0.284	0.029	0.027	0.031	0.029	0.094
globID3	0.473	0.047	0.045	0.052	0.049	0.156
globID4	0.231	0.023	0.022	0.026	0.024	0.076

Fitted Covariance Matrix

	qual2	qual3	qual4	qual5	qual6	localID1
qual2	1.693					
qual3	0.873	1.536				
qual4	1.032	0.865	1.627			
qual5	1.065	0.893	1.055	1.606		
qual6	1.010	0.847	1.000	1.033	1.619	
localID1	0.694	0.582	0.688	0.710	0.673	2.002
localID2	0.636	0.533	0.630	0.650	0.617	1.217
localID3	0.644	0.540	0.638	0.658	0.625	1.232
localID4	0.574	0.481	0.568	0.586	0.556	1.098
tn_at	6.660	5.584	6.598	6.810	6.459	5.580
price1	-0.007	-0.006	-0.007	-0.008	-0.007	0.207
price2	-0.009	-0.007	-0.009	-0.009	-0.008	0.238
globatt1	0.025	0.021	0.025	0.025	0.024	0.179

globatt2	0.020	0.017	0.020	0.021	0.019	0.145
globatt3	0.021	0.018	0.021	0.022	0.020	0.152
globID1	0.178	0.149	0.176	0.182	0.173	0.090
globID2	0.098	0.082	0.097	0.100	0.095	0.050
globID3	0.162	0.136	0.161	0.166	0.157	0.082
globID4	0.079	0.066	0.078	0.081	0.077	0.040

Fitted Covariance Matrix

	localID2	localID3	localID4	tn_at	price1	price2
localID2	2.211					
localID3	1.129	1.918				
localID4	1.006	1.018	1.637			
tn_at	5.112	5.177	4.611	453.020		
price1	0.190	0.192	0.171	-11.352	2.504	
price2	0.218	0.221	0.197	-13.051	1.810	3.022
globatt1	0.164	0.166	0.148	-1.156	0.401	0.461
globatt2	0.133	0.134	0.120	-0.935	0.324	0.373
globatt3	0.140	0.141	0.126	-0.983	0.341	0.392
globID1	0.083	0.084	0.075	-1.064	0.111	0.127
globID2	0.045	0.046	0.041	-0.584	0.061	0.070
globID3	0.076	0.077	0.068	-0.971	0.101	0.116
globID4	0.037	0.037	0.033	-0.474	0.049	0.057

Fitted Covariance Matrix

	globatt1	globatt2	globatt3	globID1	globID2	globID3
globatt1	2.155					
globatt2	1.291	2.731				
globatt3	1.357	1.098	1.775			
globID1	0.375	0.303	0.319	2.720		
globID2	0.206	0.166	0.175	1.273	1.871	
globID3	0.342	0.277	0.291	2.119	1.162	2.700

globID4	0.167	0.135	0.142	1.033	0.567	0.943

Fitted Covariance Matrix

	globID4
globID4	1.646

Fitted Residuals

	cet1	cet2	cet3	cet4	env1	env2
cet1	0.000					
cet2	0.121	0.000				
cet3	0.011	-0.011	0.000			
cet4	-0.085	-0.129	0.037	0.000		
env1	-0.034	-0.097	-0.049	0.082	0.000	
env2	-0.054	-0.001	-0.019	0.143	-0.006	0.000
env4	0.050	0.085	0.045	0.002	0.028	-0.019
aut1	0.034	-0.089	0.044	-0.052	0.003	-0.023
aut2	0.009	-0.062	0.050	-0.017	0.008	0.075
aut3	-0.057	-0.041	0.018	-0.004	0.004	-0.035
aut4	0.062	-0.022	0.084	0.018	-0.059	-0.083
qual1	-0.108	-0.025	-0.124	-0.166	-0.111	0.028
qual2	0.003	0.155	0.014	-0.072	-0.155	0.063
qual3	0.125	0.183	0.205	0.176	0.101	0.281
qual4	0.017	-0.019	0.046	-0.015	-0.021	0.024
qual5	-0.030	-0.109	-0.155	0.007	-0.143	-0.017
qual6	0.067	0.003	-0.012	0.072	0.055	0.055
localID1	-0.115	0.047	-0.176	-0.121	-0.125	-0.150
localID2	0.096	0.214	0.190	0.079	-0.023	-0.028
localID3	0.008	-0.129	-0.051	0.012	0.076	0.058
localID4	0.013	0.078	0.039	0.195	0.101	0.115
tn_at	-1.302	-1.237	-0.910	4.843	-0.264	0.057
price1	0.150	0.381	0.123	-0.165	0.000	0.070

price2	−0.039	0.072	−0.144	−0.353	−0.100	0.012
globatt1	−0.087	−0.032	−0.007	0.046	−0.107	0.062
globatt2	−0.103	−0.094	−0.164	−0.191	−0.216	−0.050
globatt3	0.067	−0.038	0.142	0.130	0.075	0.194
globID1	−0.051	−0.047	−0.065	−0.095	−0.019	0.055
globID2	0.115	0.125	0.202	0.219	0.104	0.187
globID3	0.005	−0.067	0.038	0.056	−0.052	0.021
globID4	0.154	0.092	0.226	0.251	0.132	0.228

Fitted Residuals

	env4	aut1	aut2	aut3	aut4	qual1
env4	0.000					
aut1	−0.030	0.000				
aut2	0.046	−0.006	0.000			
aut3	0.062	0.007	0.012	0.000		
aut4	−0.022	0.032	0.005	−0.057	0.000	
qual1	−0.003	−0.019	−0.073	−0.099	−0.026	0.000
qual2	−0.099	0.003	−0.015	−0.029	−0.006	0.275
qual3	0.120	−0.002	0.001	0.030	−0.018	−0.027
qual4	0.016	0.006	0.070	0.143	0.126	0.018
qual5	−0.011	−0.060	−0.029	0.009	−0.088	−0.037
qual6	0.048	−0.033	−0.018	0.078	−0.027	−0.134
localID1	−0.076	−0.097	−0.167	−0.064	0.113	−0.090
localID2	0.006	0.087	0.019	0.021	0.275	0.106
localID3	0.090	−0.090	−0.091	−0.027	0.117	−0.245
localID4	0.068	−0.009	0.067	0.165	0.121	−0.212
tn_at	0.396	−0.605	0.205	−0.094	0.893	−1.978
price1	0.115	0.155	0.075	0.148	0.144	−0.025
price2	−0.041	−0.049	−0.129	−0.118	−0.191	0.029
globatt1	−0.085	−0.075	−0.044	−0.056	−0.068	−0.176
globatt2	−0.351	0.178	0.089	0.001	0.150	0.254
globatt3	0.052	0.045	0.034	0.045	0.064	−0.032
globID1	−0.160	−0.079	−0.016	−0.097	−0.264	−0.061

globID2	0.215	0.261	0.266	0.285	0.215	0.137
globID3	-0.251	-0.038	0.049	0.005	-0.067	0.107
globID4	0.101	0.271	0.327	0.395	0.195	0.186

Fitted Residuals

	qual2	qual3	qual4	qual5	qual6	localID1
qual2	0.000					
qual3	-0.084	0.000				
qual4	-0.002	-0.038	0.000			
qual5	-0.021	-0.019	-0.023	0.000		
qual6	-0.129	0.088	-0.015	0.134	0.000	
localID1	-0.022	0.039	-0.035	-0.036	-0.011	0.000
localID2	0.166	0.087	0.051	-0.022	0.067	0.046
localID3	-0.103	0.119	0.057	-0.032	-0.064	0.036
localID4	-0.018	0.089	0.118	0.090	0.064	-0.013
tn_at	-0.071	3.015	-0.127	0.039	-0.370	-3.090
price1	0.157	0.035	0.067	0.098	0.076	0.293
price2	0.010	-0.154	-0.120	-0.016	-0.164	-0.045
globatt1	-0.168	0.092	-0.055	0.002	-0.049	-0.086
globatt2	0.111	0.023	0.061	-0.003	0.074	0.204
globatt3	-0.010	0.178	0.110	-0.007	0.097	0.001
globID1	-0.045	0.004	-0.118	-0.079	-0.069	-0.095
globID2	0.100	0.161	0.175	0.190	0.242	0.121
globID3	-0.028	0.006	-0.144	0.035	-0.019	-0.026
globID4	0.270	0.275	0.268	0.320	0.271	0.196

Fitted Residuals

	localID2	localID3	localID4	tn_at	price1	price2
localID2	0.000					
localID3	-0.063	0.000				
localID4	-0.082	0.031	0.000			

tn_at	1.652	0.830	1.953	0.000		
price1	0.085	0.161	-0.060	1.470	0.000	
price2	-0.012	-0.082	-0.334	-1.294	0.000	0.000
globatt1	-0.219	-0.118	0.120	0.288	-0.066	-0.142
globatt2	0.267	-0.002	-0.061	-3.707	0.446	0.440
globatt3	-0.065	0.122	0.143	0.922	0.040	-0.066
globID1	-0.293	0.051	0.094	-0.764	0.030	0.103
globID2	-0.065	0.239	0.254	0.791	0.155	0.026
globID3	-0.257	0.046	-0.044	0.186	-0.126	-0.114
globID4	0.085	0.364	0.513	3.544	-0.164	-0.215

Fitted Residuals

	globatt1	globatt2	globatt3	globID1	globID2	globID3
globatt1	0.000					
globatt2	0.010	0.000				
globatt3	0.012	-0.058	0.000			
globID1	-0.057	0.127	-0.041	0.000		
globID2	0.079	0.147	0.222	-0.031	0.000	
globID3	-0.075	0.153	-0.041	0.014	-0.005	0.000
globID4	0.087	0.019	0.151	-0.022	0.152	-0.020

Fitted Residuals

	globID4
globID4	0.000

Summary Statistics for Fitted Residuals

Smallest Fitted Residual = -3.707
Median Fitted Residual = 0.001
Largest Fitted Residual = 4.843

Stemleaf Plot

```
- 3|7
- 3|1
- 2|
- 2|0
- 1|
- 1|332
- 0|986
- 0|44433333322222222222222222221111111111111111111111111111111111111111111+92
  0|111111111111111111111111111111111111111111111111111111111111111111111111+90
  0|58899
  1|
  1|57
  2|0
  2|
  3|0
  3|5
  4|
  4|8
```

Standardized Residuals

	cet1	cet2	cet3	cet4	env1	env2
cet1	0.000					
cet2	0.753	0.000				
cet3	0.040	-0.061	- -			
cet4	-0.605	-0.813	0.231	0.000		
env1	-0.295	-0.800	- -	- -	0.000	
env2	-0.410	- -	-0.122	1.082	- -	0.000
env4	0.425	0.691	- -	0.015	- -	- -
aut1	0.293	-0.812	0.346	-0.451	0.032	-0.204
aut2	- -	-0.723	0.457	-0.158	0.092	0.732
aut3	-0.458	- -	0.166	-0.041	0.042	-0.313

aut4	0.541	-0.158	0.513	- -	-0.643	-0.871
qual1	-0.790	-0.198	-0.885	-1.359	-1.026	0.308
qual2	0.027	- -	0.125	-0.635	-1.531	0.539
qual3	1.050	1.723	1.781	1.477	1.065	2.796
qual4	0.139	-0.161	0.341	-0.122	-0.213	- -
qual5	- -	-0.833	-1.690	0.060	- -	-0.149
qual6	0.542	0.027	-0.089	0.584	0.553	0.541
localID1	-0.913	0.327	-1.199	-1.236	-1.214	-2.106
localID2	0.694	1.454	1.244	0.562	-0.202	-0.371
localID3	0.060	-0.936	-0.356	0.092	0.717	0.480
localID4	- -	0.664	0.294	1.607	- -	1.023
tn_at	-0.760	-0.566	-0.395	2.306	-0.161	0.030
price1	1.168	2.573	0.793	-1.139	-0.002	0.626
price2	-0.247	0.438	-0.839	-2.216	-0.770	0.077
globatt1	-0.663	-0.227	-0.047	0.347	-1.043	0.508
globatt2	-0.693	-0.596	-0.960	-1.314	-1.892	-0.358
globatt3	0.563	-0.372	1.092	1.075	0.766	1.752
globID1	-0.344	-0.300	-0.585	-0.632	-0.221	0.396
globID2	0.939	0.957	1.520	1.763	1.012	1.606
globID3	0.032	-0.431	0.249	0.376	-0.427	0.150
globID4	1.337	0.752	1.798	0.969	2.534	5.588

Standardized Residuals

	env4	aut1	aut2	aut3	aut4	qual1
env4	- -					
aut1	-0.259	0.000				
aut2	0.425	-0.057	0.000			
aut3	0.579	0.058	0.113	0.000		
aut4	-0.184	0.277	0.042	-0.451	0.000	
qual1	- -	-0.177	-0.643	-0.860	-0.221	- -
qual2	-0.798	0.030	-0.151	-0.253	-0.054	2.006
qual3	1.020	-0.018	0.007	0.299	-0.179	-0.251
qual4	0.142	0.069	- -	1.270	1.078	0.147

qual5	-0.092	-0.572	-0.294	0.083	- -	-0.296
qual6	0.398	-0.421	- -	1.076	-0.233	-1.099
localID1	-0.572	-0.841	-1.545	-0.515	- -	-0.667
localID2	- -	0.982	0.174	0.169	2.091	0.812
localID3	0.695	-2.401	-0.857	-0.288	1.545	-2.010
localID4	0.666	- -	0.696	1.576	1.182	- -
tn_at	0.196	-0.371	0.135	-0.055	0.683	-1.085
price1	0.789	1.524	0.677	1.197	1.093	-0.225
price2	-0.056	-0.378	-1.054	-1.310	-1.507	0.202
globatt1	-0.625	-0.710	-0.436	-0.492	-0.558	-1.433
globatt2	-2.324	1.453	0.777	- -	1.087	1.838
globatt3	0.435	- -	0.369	0.427	0.852	-0.306
globID1	-1.049	-0.647	-0.141	-0.753	-1.923	-0.434
globID2	1.818	2.578	- -	2.665	1.905	1.201
globID3	-1.847	-0.323	0.456	0.036	-0.548	0.780
globID4	0.859	2.853	3.660	3.933	1.868	1.766

Standardized Residuals

	qual2	qual3	qual4	qual5	qual6	localID1
qual2	- -					
qual3	-0.874	0.000				
qual4	-0.014	-0.335	0.000			
qual5	-0.191	- -	-0.239	0.000		
qual6	-1.089	0.776	-0.129	1.116	0.000	
localID1	-0.179	0.376	-0.279	-0.484	-0.091	0.000
localID2	1.446	- -	0.406	-0.181	0.650	0.308
localID3	-0.856	1.053	0.605	-0.278	-0.633	0.279
localID4	-0.158	0.912	1.124	0.827	0.591	- -
tn_at	-0.040	1.778	-0.072	0.022	-0.211	-1.605
price1	1.220	0.286	0.160	0.775	0.595	2.118
price2	0.067	-1.135	-0.864	-0.117	-1.177	-0.292
globatt1	-1.284	0.805	-0.470	0.012	-0.545	-0.873
globatt2	0.827	0.205	0.461	-0.020	0.645	1.440

globatt3	-0.095	1.695	1.978	-0.062	0.933	0.005
globID1	-0.330	0.032	-0.963	-0.633	-1.283	-0.650
globID2	0.896	1.511	1.595	1.733	2.242	0.998
globID3	-0.220	0.046	-1.850	0.267	-0.147	-0.181
globID4	2.787	2.796	3.334	3.197	2.644	1.716

Standardized Residuals

	localID2	localID3	localID4	tn_at	price1	price2
localID2	- -					
localID3	- -	0.000				
localID4	-0.603	0.243	0.000			
tn_at	0.820	0.447	1.134	0.000		
price1	0.573	1.162	-0.489	0.218	0.000	
price2	-0.075	- -	-2.462	-0.567	0.000	0.000
globatt1	-1.578	-0.933	1.013	0.146	-0.443	-0.870
globatt2	1.727	-0.017	-0.456	-1.676	2.678	2.482
globatt3	-0.518	1.036	0.667	0.526	0.298	-0.446
globID1	-1.896	0.356	0.710	-0.346	0.185	0.575
globID2	-0.525	2.281	2.556	0.433	1.141	0.177
globID3	-1.675	0.339	-0.341	0.098	-0.771	-0.637
globID4	0.710	3.255	4.968	2.064	-1.287	-1.511

Standardized Residuals

	globatt1	globatt2	globatt3	globID1	globID2	globID3
globatt1	0.000					
globatt2	0.059	0.000				
globatt3	0.080	-0.374	0.000			
globID1	-0.361	0.707	-0.284	0.000		
globID2	0.626	1.029	1.831	-0.193	0.000	
globID3	-0.495	0.910	-0.297	0.066	-0.029	0.000
globID4	0.717	0.144	1.206	-0.428	1.342	-0.136

```
        Standardized Residuals

            globID4
           --------
 globID4      0.000

Summary Statistics for Standardized Residuals

Smallest Standardized Residual =   -2.462
  Median Standardized Residual =    0.000
 Largest Standardized Residual =    5.588

Stemleaf Plot

- 2|5
- 2|43210
- 1|99988777665555
- 1|4433333222211111100000
- 0|99999999999988888888888877777666666666666666666655555555555555
- 0|444444444444444444333333333333333333333222222222222222222222222222111111+04
  0|1111111111111111111111222222222222233333333333333344444444444444
  0|5555555555566666666666667777777777777777888888888888889999999
  1|00000000001111111111111122222222233344
  1|555555566667777788888888899
  2|00111233
  2|556666778889
  3|233
  3|79
  4|
  4|
  5|0
  5|6
Largest Positive Standardized Residuals
Residual for    qual3 and     env2   2.796
```

```
Residual for globatt2 and   price1   2.678
Residual for  globID2 and     aut1   2.578
Residual for  globID2 and     aut3   2.665
Residual for  globID4 and     env2   5.588
Residual for  globID4 and     aut1   2.853
Residual for  globID4 and     aut2   3.660
Residual for  globID4 and     aut3   3.933
Residual for  globID4 and    qual2   2.787
Residual for  globID4 and    qual3   2.796
Residual for  globID4 and    qual4   3.334
Residual for  globID4 and    qual5   3.197
Residual for  globID4 and    qual6   2.644
Residual for  globID4 and localID3   3.255
Residual for  globID4 and localID4   4.968

!HV Daten aus Studie 1

                        Qplot of Standardized Residuals

 3.5..........................................................................
    .                                                                       ..
    .                                                                      . .
    .                                                                    .   .
    .                                                                  .     .
    .                                                                 .      .
    .                                                               .        *
    .                                                             .          x
    .                                                            .        xx x
    .                                                          .      x   x  .
    .                                                        .      x x      .
    .                                                       .      **        .
    .                                                     .     *xx*         .
    .                                                   .   ***x             .
    .                                                  .  *x                 .
N   .                                                .  *xx                  .
```

```
o   .                                              .x*x                      .
r   .                                             *xx                        .
m   .                                           *x*                          .
a   .                                         x*x                            .
l   .                                       x*x                              .
    .                                     xx*                                .
Q   .                                    *x                                  .
u   .                                   *x                                   .
a   .                                 .xx                                    .
n   .                               . xx                                     .
t   .                              . **                                      .
i   .                            . xxx                                       .
l   .                          .  xx                                         .
e   .                         . *xx                                          .
s   .                       .  xx                                            .
    .                     .  *x                                              .
    .                    . x*                                                .
    .                  .xx*                                                  .
    .                . **                                                    .
    .               .x x                                                     .
    .             . xx                                                       .
    .           .x xx                                                        .
    .          .x                                                            .
    .        . *                                                             .
    .      .                                                                 .
    .     .                                                                  .
    .   .                                                                    .
    . .                                                                      .
-3.5..........................................................................
  -3.5                                                                      3.5
                            Standardized Residuals
```

!HV Daten aus Studie 1

Modification Indices and Expected Change

The Modification Indices Suggest to Add the

Path to	from	Decrease in Chi-Square	New Estimate
cet4	PRICESEN	13.0	-0.21
cet4	NUTZEN	28.0	0.02
aut4	LOCALISM	10.6	0.25
qual3	ETHNO	12.6	0.27
qual3	GREEN	12.0	0.28
localID1	ETHNO	7.9	-0.20
localID1	GREEN	9.7	-0.26
localID1	NUTZEN	12.9	-0.01
localID4	PRICESEN	8.2	-0.16
globatt2	PRICESEN	17.5	0.34
globID1	AUTHENTI	12.6	-0.24
globID2	AUTHENTI	13.7	0.27
globID4	AUTHENTI	20.3	0.33
globID4	QUALITY	16.3	0.31
globID4	LOCALISM	16.4	0.26

Modification Indices for LAMBDA-X

	ETHNO	GREEN	AUTHENTI	QUALITY	LOCALISM	PRICESEN
cet1	- -	0.495	0.001	0.082	0.085	0.885
cet2	- -	0.570	1.231	0.116	0.499	6.734
cet3	- -	0.071	1.257	0.183	0.260	0.022
cet4	- -	3.778	0.080	0.028	0.071	13.021
env1	1.091	- -	0.129	3.590	0.051	1.203
env2	0.026	- -	0.018	2.896	0.009	0.758
env4	0.952	- -	0.306	0.040	0.137	0.062
aut1	0.097	0.248	- -	0.775	1.715	0.733
aut2	0.021	2.185	- -	0.180	4.200	0.720
aut3	0.322	0.000	- -	1.680	0.425	0.042
aut4	0.906	1.505	- -	0.013	10.551	0.079
qual1	5.230	0.928	3.460	- -	7.055	0.015

qual2	0.074	1.695	0.250	- -	0.027	1.061
qual3	12.608	11.991	0.368	- -	2.922	1.156
qual4	0.174	0.062	7.467	- -	1.406	0.142
qual5	3.933	3.404	1.795	- -	0.045	0.460
qual6	0.498	1.471	0.130	- -	0.036	0.447
localID1	7.881	9.747	6.635	1.356	- -	5.023
localID2	5.100	0.036	3.017	2.432	- -	0.006
localID3	0.269	1.510	1.236	1.307	- -	0.038
localID4	2.657	5.972	5.866	1.122	- -	8.164
tn_at	- -	- -	- -	- -	- -	- -
price1	6.053	2.125	7.219	3.374	7.701	- -
price2	6.053	2.125	7.219	3.374	7.701	- -
globatt1	0.655	1.203	3.223	3.460	4.159	5.273
globatt2	1.852	3.634	0.895	0.922	1.479	17.509
globatt3	3.105	5.915	1.543	1.692	1.716	0.234
globID1	4.963	2.224	12.560	7.862	3.983	2.872
globID2	4.683	6.227	13.745	7.444	5.819	0.741
globID3	0.001	1.112	0.041	0.074	0.946	2.417
globID4	7.172	7.659	20.296	16.328	16.376	2.917

Modification Indices for LAMBDA-X

	NUTZEN	GLOBAL	GID
cet1	3.725	0.369	0.003
cet2	1.726	0.256	0.072
cet3	1.704	0.257	0.036
cet4	27.952	0.391	0.011
env1	0.171	1.571	0.008
env2	0.007	4.473	2.493
env4	0.145	1.058	3.031
aut1	0.787	0.009	0.010
aut2	0.105	0.007	1.756
aut3	0.025	0.063	0.056
aut4	0.821	0.013	3.396

qual1	3.461	0.984	0.116
qual2	0.006	1.420	0.004
qual3	7.819	2.975	0.435
qual4	0.021	0.137	1.065
qual5	0.002	0.000	0.029
qual6	0.160	0.124	0.012
localID1	12.928	0.074	0.310
localID2	1.800	1.885	7.421
localID3	0.715	0.008	2.097
localID4	3.975	2.825	2.659
tn_at	- -	- -	- -
price1	5.398	0.656	0.051
price2	5.398	0.656	0.051
globatt1	0.178	- -	0.728
globatt2	5.139	- -	1.248
globatt3	1.175	- -	0.016
globID1	2.179	0.643	- -
globID2	0.317	3.190	- -
globID3	0.034	0.441	- -
globID4	6.151	1.436	- -

Expected Change for LAMBDA-X

	ETHNO	GREEN	AUTHENTI	QUALITY	LOCALISM	PRICESEN
cet1	- -	-0.067	0.003	0.026	-0.020	0.050
cet2	- -	-0.083	-0.113	0.036	0.055	0.160
cet3	- -	-0.028	0.108	-0.042	-0.037	-0.009
cet4	- -	0.205	-0.028	-0.017	0.020	-0.214
env1	-0.076	- -	-0.028	-0.140	-0.013	-0.054
env2	0.014	- -	-0.012	0.145	-0.006	0.049
env4	0.089	- -	0.054	0.019	0.028	0.016
aut1	-0.018	-0.034	- -	-0.073	-0.082	0.034
aut2	0.008	0.094	- -	-0.033	-0.120	-0.032
aut3	-0.033	0.001	- -	0.111	0.041	0.008

aut4	0.069	-0.104	- -	-0.012	0.253	-0.014
qual1	-0.175	-0.081	-0.198	- -	-0.201	0.007
qual2	0.019	-0.101	-0.049	- -	-0.012	0.051
qual3	0.265	0.284	0.063	- -	0.127	-0.057
qual4	0.029	0.019	0.262	- -	0.081	-0.018
qual5	-0.131	-0.133	-0.123	- -	-0.014	0.031
qual6	0.049	0.093	0.035	- -	0.013	-0.033
localID1	-0.201	-0.258	-0.266	-0.112	- -	0.128
localID2	0.180	-0.018	0.196	0.165	- -	0.005
localID3	-0.037	0.102	-0.113	-0.109	- -	0.011
localID4	0.109	0.190	0.231	0.094	- -	-0.156
tn_at	- -	- -	- -	- -	- -	- -
price1	0.190	0.143	0.227	0.159	0.213	- -
price2	-0.218	-0.165	-0.261	-0.183	-0.245	- -
globatt1	-0.049	-0.085	-0.130	-0.138	-0.134	-0.151
globatt2	-0.104	-0.185	0.086	0.089	0.100	0.338
globatt3	0.095	0.168	0.080	0.086	0.076	-0.028
globID1	-0.127	-0.118	-0.240	-0.197	-0.121	0.097
globID2	0.133	0.209	0.273	0.208	0.159	0.054
globID3	0.002	-0.082	-0.014	-0.019	-0.059	-0.089
globID4	0.164	0.230	0.329	0.306	0.264	-0.106

Expected Change for LAMBDA-X

	NUTZEN	GLOBAL	GID
cet1	-0.007	-0.030	-0.002
cet2	-0.005	-0.029	-0.013
cet3	-0.005	0.027	0.008
cet4	0.020	0.035	0.005
env1	-0.001	-0.058	-0.004
env2	0.000	0.111	0.071
env4	0.001	-0.062	-0.089
aut1	-0.002	0.004	-0.003
aut2	0.001	0.003	0.039

aut3	0.000	-0.010	0.007
aut4	0.003	0.006	-0.073
qual1	-0.006	-0.052	0.014
qual2	0.000	-0.058	-0.002
qual3	0.008	0.089	0.027
qual4	0.000	0.017	-0.039
qual5	0.000	-0.001	0.006
qual6	-0.001	0.017	0.004
localID1	-0.011	-0.015	-0.025
localID2	0.005	-0.087	-0.137
localID3	0.003	-0.005	0.064
localID4	0.006	0.089	0.069
tn_at	- -	- -	- -
price1	0.014	0.062	-0.013
price2	-0.016	-0.072	0.014
globatt1	0.001	- -	-0.043
globatt2	-0.009	- -	0.069
globatt3	0.003	- -	0.006
globID1	-0.005	-0.047	- -
globID2	0.002	0.111	- -
globID3	0.001	-0.038	- -
globID4	0.008	0.074	- -

Standardized Expected Change for LAMBDA-X

	ETHNO	GREEN	AUTHENTI	QUALITY	LOCALISM	PRICESEN
cet1	- -	-0.065	0.003	0.026	-0.023	0.063
cet2	- -	-0.080	-0.113	0.035	0.064	0.201
cet3	- -	-0.027	0.108	-0.042	-0.043	-0.011
cet4	- -	0.198	-0.028	-0.016	0.023	-0.269
env1	-0.092	- -	-0.028	-0.137	-0.015	-0.068
env2	0.016	- -	-0.012	0.141	-0.007	0.062
env4	0.107	- -	0.054	0.018	0.032	0.020
aut1	-0.022	-0.033	- -	-0.072	-0.094	0.043

aut2	0.009	0.090	- -	-0.032	-0.138	-0.040
aut3	-0.040	0.001	- -	0.108	0.047	0.010
aut4	0.083	-0.100	- -	-0.011	0.292	-0.018
qual1	-0.210	-0.078	-0.198	- -	-0.232	0.008
qual2	0.023	-0.097	-0.049	- -	-0.013	0.064
qual3	0.319	0.274	0.063	- -	0.146	-0.071
qual4	0.034	0.018	0.262	- -	0.093	-0.023
qual5	-0.157	-0.128	-0.123	- -	-0.016	0.039
qual6	0.059	0.090	0.035	- -	0.015	-0.041
localID1	-0.241	-0.248	-0.265	-0.109	- -	0.161
localID2	0.217	-0.017	0.196	0.162	- -	0.006
localID3	-0.044	0.098	-0.113	-0.106	- -	0.014
localID4	0.131	0.184	0.230	0.092	- -	-0.195
tn_at	- -	- -	- -	- -	- -	- -
price1	0.228	0.138	0.227	0.155	0.246	- -
price2	-0.262	-0.159	-0.261	-0.179	-0.282	- -
globatt1	-0.059	-0.082	-0.129	-0.135	-0.154	-0.190
globatt2	-0.124	-0.179	0.086	0.088	0.115	0.424
globatt3	0.114	0.161	0.080	0.084	0.088	-0.035
globID1	-0.152	-0.114	-0.240	-0.193	-0.139	0.122
globID2	0.160	0.201	0.272	0.203	0.183	0.067
globID3	0.002	-0.079	-0.014	-0.019	-0.068	-0.112
globID4	0.197	0.222	0.329	0.299	0.305	-0.133

Standardized Expected Change for LAMBDA-X

	NUTZEN	GLOBAL	GID
cet1	-0.141	-0.038	-0.003
cet2	-0.111	-0.037	-0.019
cet3	-0.103	0.034	0.013
cet4	0.428	0.044	0.007
env1	-0.024	-0.073	-0.005
env2	0.005	0.141	0.109
env4	0.029	-0.078	-0.136

aut1	-0.043	0.005	-0.005
aut2	0.015	0.004	0.059
aut3	-0.008	-0.012	0.011
aut4	0.055	0.007	-0.111
qual1	-0.120	-0.066	0.022
qual2	-0.005	-0.073	-0.004
qual3	0.176	0.112	0.042
qual4	-0.008	0.022	-0.059
qual5	0.003	-0.001	0.009
qual6	-0.023	0.021	0.006
localID1	-0.238	-0.019	-0.037
localID2	0.101	-0.110	-0.209
localID3	0.056	-0.006	0.098
localID4	0.125	0.112	0.105
tn_at	- -	- -	- -
price1	0.288	0.079	-0.019
price2	-0.332	-0.090	0.022
globatt1	0.029	- -	-0.065
globatt2	-0.198	- -	0.105
globatt3	0.067	- -	0.009
globID1	-0.096	-0.059	- -
globID2	0.040	0.141	- -
globID3	0.012	-0.048	- -
globID4	0.174	0.094	- -

Completely Standardized Expected Change for LAMBDA-X

	ETHNO	GREEN	AUTHENTI	QUALITY	LOCALISM	PRICESEN
cet1	- -	-0.046	0.002	0.018	-0.016	0.044
cet2	- -	-0.053	-0.075	0.023	0.042	0.133
cet3	- -	-0.017	0.070	-0.027	-0.028	-0.007
cet4	- -	0.137	-0.019	-0.011	0.016	-0.186
env1	-0.078	- -	-0.024	-0.117	-0.013	-0.058
env2	0.012	- -	-0.009	0.105	-0.006	0.046

env4	0.073	- -	0.037	0.013	0.022	0.014
aut1	-0.018	-0.028	- -	-0.061	-0.080	0.037
aut2	0.009	0.082	- -	-0.029	-0.125	-0.036
aut3	-0.032	0.001	- -	0.087	0.038	0.008
aut4	0.062	-0.076	- -	-0.009	0.220	-0.013
qual1	-0.158	-0.059	-0.149	- -	-0.175	0.006
qual2	0.018	-0.075	-0.038	- -	-0.010	0.049
qual3	0.257	0.221	0.051	- -	0.118	-0.057
qual4	0.027	0.014	0.205	- -	0.073	-0.018
qual5	-0.124	-0.101	-0.097	- -	-0.012	0.031
qual6	0.047	0.070	0.028	- -	0.012	-0.032
localID1	-0.171	-0.176	-0.187	-0.077	- -	0.114
localID2	0.146	-0.011	0.132	0.109	- -	0.004
localID3	-0.032	0.071	-0.082	-0.077	- -	0.010
localID4	0.103	0.143	0.180	0.072	- -	-0.153
tn_at	- -	- -	- -	- -	- -	- -
price1	0.144	0.087	0.143	0.098	0.155	- -
price2	-0.151	-0.091	-0.150	-0.103	-0.162	- -
globatt1	-0.040	-0.056	-0.088	-0.092	-0.105	-0.129
globatt2	-0.075	-0.108	0.052	0.053	0.069	0.256
globatt3	0.085	0.121	0.060	0.063	0.066	-0.027
globID1	-0.092	-0.069	-0.145	-0.117	-0.085	0.074
globID2	0.117	0.147	0.199	0.148	0.134	0.049
globID3	0.001	-0.048	-0.008	-0.011	-0.041	-0.068
globID4	0.154	0.173	0.256	0.233	0.238	-0.104

Completely Standardized Expected Change for LAMBDA-X

	NUTZEN	GLOBAL	GID
cet1	-0.099	-0.027	-0.002
cet2	-0.073	-0.025	-0.013
cet3	-0.067	0.022	0.008
cet4	0.297	0.031	0.005
env1	-0.021	-0.062	-0.005

env2	0.004	0.105	0.081
env4	0.020	−0.054	−0.093
aut1	−0.036	0.004	−0.004
aut2	0.013	0.004	0.054
aut3	−0.006	−0.010	0.009
aut4	0.042	0.005	−0.084
qual1	−0.090	−0.049	0.017
qual2	−0.004	−0.056	−0.003
qual3	0.142	0.090	0.034
qual4	−0.006	0.017	−0.047
qual5	0.002	−0.001	0.007
qual6	−0.018	0.017	0.005
localID1	−0.168	−0.014	−0.026
localID2	0.068	−0.074	−0.140
localID3	0.041	−0.005	0.071
localID4	0.098	0.088	0.082
tn_at	− −	− −	− −
price1	0.182	0.050	−0.012
price2	−0.191	−0.052	0.013
globatt1	0.020	− −	−0.044
globatt2	−0.120	− −	0.064
globatt3	0.050	− −	0.006
globID1	−0.058	−0.036	− −
globID2	0.029	0.103	− −
globID3	0.007	−0.029	− −
globID4	0.136	0.073	− −

No Non−Zero Modification Indices for PHI

The Modification Indices Suggest to Add an Error Covariance

Between	and	Decrease in Chi−Square	New Estimate
cet2	cet1	13.5	0.24
qual2	cet2	9.8	0.18
qual2	qual1	52.2	0.40
qual6	qual1	12.5	−0.19

qual6	qual2	15.5	-0.20
qual6	qual5	23.6	0.23
localID1	cet2	8.3	0.18
localID3	cet2	9.6	-0.20
localID4	aut3	8.9	0.12
localID4	qual1	13.4	-0.20
tn_at	cet4	19.1	4.88
globatt1	localID4	9.7	0.18
globatt2	qual1	12.5	0.29
globatt2	localID2	11.1	0.32
globatt2	localID4	12.8	-0.29
globatt3	globatt1	9.2	1.06
globID3	localID4	8.0	-0.17
globID4	localID4	17.8	0.28

Modification Indices for THETA-DELTA

	cet1	cet2	cet3	cet4	env1	env2
cet1	- -					
cet2	13.477	- -				
cet3	0.223	0.101	- -			
cet4	6.919	7.795	1.212	- -		
env1	0.120	2.092	0.340	1.708	- -	
env2	2.199	0.472	0.335	3.478	0.249	- -
env4	0.624	2.230	0.054	4.784	1.475	0.541
aut1	3.102	1.665	0.892	1.273	1.272	0.086
aut2	0.044	0.848	0.569	0.147	0.725	4.156
aut3	4.575	0.351	0.001	0.472	0.546	2.622
aut4	0.521	0.128	0.129	0.014	0.115	1.050
qual1	0.279	1.340	0.036	2.354	0.878	0.585
qual2	0.214	9.761	0.199	3.313	5.807	5.047
qual3	0.832	0.269	1.592	0.792	0.044	6.441
qual4	0.023	1.244	1.750	0.323	0.467	1.180
qual5	0.767	2.800	6.878	3.853	3.240	0.015

qual6	1.190	0.633	0.795	1.776	5.097	1.555
localID1	0.000	8.255	1.853	0.926	0.442	1.090
localID2	0.181	1.350	2.688	0.700	0.048	0.135
localID3	1.409	9.600	0.076	0.234	1.672	0.070
localID4	2.236	0.155	0.491	7.799	0.309	0.151
tn_at	2.925	0.210	2.209	19.081	0.170	0.078
price1	0.070	6.021	0.000	1.925	0.311	0.802
price2	0.319	0.037	0.463	0.131	0.070	1.017
globatt1	1.562	0.878	0.005	1.244	1.905	0.187
globatt2	0.589	0.477	0.895	2.740	1.086	0.377
globatt3	0.140	5.521	1.225	0.397	1.520	0.426
globID1	0.663	2.074	0.219	3.998	1.009	0.582
globID2	1.090	0.132	0.117	1.266	0.739	0.158
globID3	0.033	1.281	0.568	1.252	0.064	0.004
globID4	1.247	2.578	0.017	1.012	0.364	0.078

Modification Indices for THETA-DELTA

	env4	aut1	aut2	aut3	aut4	qual1
env4	- -					
aut1	1.501	- -				
aut2	0.098	0.143	- -			
aut3	1.979	0.246	0.977	- -		
aut4	0.002	1.635	0.039	6.908	- -	
qual1	0.412	3.167	0.201	4.767	0.643	- -
qual2	1.979	1.545	0.037	2.363	0.074	52.197
qual3	0.645	0.042	0.144	0.094	0.639	0.359
qual4	0.000	3.312	0.327	4.287	2.708	0.248
qual5	1.198	0.332	0.100	1.027	2.776	1.279
qual6	0.013	0.955	0.628	4.126	0.821	12.486
localID1	0.233	0.006	6.149	0.034	3.262	2.099
localID2	0.000	2.093	0.000	4.698	4.148	7.661
localID3	0.247	0.911	0.365	0.013	1.167	6.024
localID4	1.285	4.100	2.056	8.909	1.716	13.363

tn_at	0.023	0.374	0.037	0.025	0.091	1.343
price1	0.794	0.025	0.044	0.240	0.995	6.720
price2	0.008	0.424	0.022	0.263	3.292	5.113
globatt1	0.201	1.442	0.083	0.648	0.429	3.919
globatt2	4.873	5.837	0.269	3.170	1.401	12.479
globatt3	0.050	0.054	0.386	0.001	0.091	0.669
globID1	0.000	0.341	0.007	1.595	5.387	1.082
globID2	4.768	0.640	0.108	0.377	0.780	0.597
globID3	4.446	0.844	0.010	0.053	2.678	5.455
globID4	0.098	0.205	0.563	4.500	0.662	1.802

Modification Indices for THETA-DELTA

	qual2	qual3	qual4	qual5	qual6	localID1
qual2	- -					
qual3	4.674	- -				
qual4	0.003	1.040	- -			
qual5	0.565	0.316	0.751	- -		
qual6	15.537	5.209	0.246	23.560	- -	
localID1	0.290	0.025	1.810	0.038	0.203	- -
localID2	5.217	0.480	1.478	4.500	0.007	1.664
localID3	2.505	3.778	2.499	0.165	0.777	1.814
localID4	1.732	0.002	1.827	3.348	0.405	0.214
tn_at	0.055	2.119	0.309	1.357	1.207	6.633
price1	0.343	0.547	0.021	0.256	0.723	2.457
price2	0.324	0.212	0.677	0.555	2.419	2.245
globatt1	3.047	1.251	0.399	7.405	0.189	0.026
globatt2	2.421	3.430	0.210	2.239	0.039	4.410
globatt3	0.044	1.535	2.120	4.553	1.018	2.207
globID1	1.580	0.128	0.426	1.562	0.374	0.274
globID2	2.077	0.049	0.665	0.026	1.991	0.434
globID3	0.117	0.529	4.330	1.548	0.154	2.478
globID4	0.033	0.305	0.576	1.173	0.072	2.878

Modification Indices for THETA-DELTA

	localID2	localID3	localID4	tn_at	price1	price2
localID2	- -					
localID3	2.252	- -				
localID4	3.644	0.880	- -			
tn_at	0.404	2.452	0.513	- -		
price1	0.751	0.458	0.228	0.529	- -	
price2	3.924	0.165	2.975	0.529	- -	- -
globatt1	3.248	3.188	9.710	0.012	1.004	0.547
globatt2	11.106	2.188	12.791	0.275	1.184	1.432
globatt3	0.747	5.084	0.343	0.057	0.699	0.194
globID1	0.137	0.001	1.466	0.008	0.004	3.549
globID2	2.057	0.242	0.985	0.056	3.538	1.234
globID3	0.005	0.000	7.976	0.155	0.602	0.752
globID4	1.378	0.841	17.796	0.908	0.273	2.039

Modification Indices for THETA-DELTA

	globatt1	globatt2	globatt3	globID1	globID2	globID3
globatt1	- -					
globatt2	0.421	- -				
globatt3	9.211	7.317	- -			
globID1	0.040	0.504	2.816	- -		
globID2	0.802	0.114	5.680	3.160	- -	
globID3	0.039	1.436	0.299	7.486	0.017	- -
globID4	0.120	1.704	2.488	1.108	4.822	0.208

Modification Indices for THETA-DELTA

	globID4
globID4	- -

Expected Change for THETA-DELTA

	cet1	cet2	cet3	cet4	env1	env2
cet1	- -					
cet2	0.242	- -				
cet3	0.032	-0.023	- -			
cet4	-0.166	-0.197	0.075	- -		
env1	0.015	-0.074	-0.027	0.065	- -	
env2	-0.073	0.040	-0.031	0.105	-0.038	- -
env4	0.046	0.104	0.014	-0.146	0.086	-0.060
aut1	0.068	-0.059	0.039	-0.050	0.039	-0.012
aut2	0.008	-0.039	0.029	-0.016	-0.027	0.075
aut3	-0.081	0.027	-0.002	0.030	0.025	-0.063
aut4	0.035	-0.021	0.019	-0.007	-0.015	-0.051
qual1	-0.028	0.072	-0.011	-0.092	-0.044	0.041
qual2	-0.022	0.179	0.023	-0.100	-0.103	0.109
qual3	-0.047	0.032	0.069	0.053	0.010	0.133
qual4	-0.007	-0.062	0.066	-0.030	0.028	-0.051
qual5	0.038	-0.088	-0.123	0.099	-0.071	-0.005
qual6	0.052	-0.045	-0.045	0.072	0.096	-0.060
localID1	0.000	0.180	-0.077	-0.058	-0.031	-0.056
localID2	-0.026	0.086	0.109	-0.060	-0.012	-0.023
localID3	0.064	-0.200	-0.016	0.030	0.063	0.015
localID4	-0.077	-0.024	-0.039	0.165	0.026	0.020
tn_at	-1.730	-0.532	-1.626	4.884	-0.372	0.288
price1	-0.017	0.188	-0.001	-0.103	-0.033	-0.060
price2	0.040	-0.016	-0.051	-0.029	-0.017	0.074
globatt1	-0.069	0.062	0.004	0.071	-0.069	0.025
globatt2	0.058	0.062	-0.076	-0.143	-0.070	0.047
globatt3	0.019	-0.144	0.061	0.037	0.057	0.034
globID1	0.043	0.090	-0.026	-0.120	0.048	0.041
globID2	-0.064	-0.027	0.022	0.079	-0.047	-0.025
globID3	0.010	-0.075	0.045	0.071	-0.013	-0.003

globID4	-0.068	-0.117	-0.008	0.071	-0.033	0.017

Expected Change for THETA-DELTA

	env4	aut1	aut2	aut3	aut4	qual1
env4	- -					
aut1	-0.057	- -				
aut2	-0.014	-0.013	- -			
aut3	0.064	0.019	0.036	- -		
aut4	0.003	0.055	0.008	-0.117	- -	
qual1	0.041	0.074	-0.018	-0.090	0.043	- -
qual2	-0.082	0.048	0.007	-0.058	0.013	0.397
qual3	-0.050	-0.008	-0.015	-0.012	-0.042	-0.035
qual4	-0.001	-0.067	0.020	0.076	0.078	0.027
qual5	0.058	-0.020	0.010	0.035	-0.075	-0.058
qual6	-0.007	-0.037	-0.028	0.076	-0.044	-0.191
localID1	0.031	0.003	-0.098	-0.008	0.097	0.083
localID2	-0.001	0.072	-0.001	-0.106	0.129	0.187
localID3	0.032	-0.041	-0.024	-0.005	0.060	-0.144
localID4	-0.071	-0.083	0.055	0.121	-0.069	-0.205
tn_at	0.177	-0.471	0.139	0.121	0.294	-1.196
price1	0.070	0.008	-0.010	0.025	0.066	-0.181
price2	-0.008	0.037	0.008	-0.028	-0.130	0.172
globatt1	0.030	-0.053	0.012	0.035	-0.037	-0.119
globatt2	-0.201	0.145	0.029	-0.105	0.091	0.289
globatt3	0.014	-0.010	-0.024	-0.001	0.016	-0.046
globID1	-0.001	0.024	0.003	-0.052	-0.124	-0.059
globID2	0.162	0.039	0.015	0.030	0.056	-0.052
globID3	-0.142	-0.041	0.004	0.010	0.093	0.140
globID4	-0.023	-0.022	0.034	0.102	-0.051	-0.089

Expected Change for THETA-DELTA

	qual2	qual3	qual4	qual5	qual6	localID1

qual2	- -					
qual3	-0.116	- -				
qual4	-0.003	-0.053	- -			
qual5	-0.036	-0.028	-0.041	- -		
qual6	-0.199	0.120	-0.024	0.230	- -	
localID1	0.028	-0.009	-0.068	0.009	0.023	- -
localID2	0.141	-0.046	-0.073	-0.120	-0.005	0.106
localID3	-0.085	0.112	0.082	0.020	-0.047	0.106
localID4	-0.068	-0.002	0.067	0.086	0.032	-0.033
tn_at	0.223	1.472	-0.513	1.023	-1.031	-2.795
price1	0.038	0.051	0.009	-0.030	0.054	0.112
price2	0.040	-0.034	-0.056	0.048	-0.107	-0.117
globatt1	-0.096	0.066	-0.034	0.137	-0.024	-0.010
globatt2	0.117	-0.149	-0.033	-0.103	-0.015	0.173
globatt3	-0.011	0.068	0.072	-0.100	0.051	-0.084
globID1	0.065	0.020	0.033	-0.060	-0.031	-0.030
globID2	-0.088	-0.015	0.048	0.009	0.085	-0.044
globID3	-0.019	-0.043	-0.111	0.063	-0.021	0.095
globID4	0.011	0.036	0.045	0.060	0.016	-0.113

Expected Change for THETA-DELTA

	localID2	localID3	localID4	tn_at	price1	price2
localID2	- -					
localID3	-0.120	- -				
localID4	-0.142	0.065	- -			
tn_at	0.785	1.712	0.740	- -		
price1	-0.072	0.049	-0.033	1.327	- -	
price2	0.180	0.032	-0.130	-1.525	- -	- -
globatt1	-0.129	-0.111	0.185	0.127	-0.076	-0.061
globatt2	0.323	-0.125	-0.288	-0.775	0.110	0.131
globatt3	-0.057	0.131	0.032	0.251	0.058	-0.034
globID1	-0.025	-0.002	0.068	0.097	-0.004	0.145

globID2	-0.113	0.034	0.065	-0.284	0.154	-0.099
globID3	0.005	0.001	-0.167	-0.432	-0.057	-0.070
globID4	-0.092	0.063	0.276	1.133	-0.043	-0.126

Expected Change for THETA-DELTA

	globatt1	globatt2	globatt3	globID1	globID2	globID3
globatt1	- -					
globatt2	0.154	- -				
globatt3	1.065	-0.526	- -			
globID1	0.012	0.058	-0.094	- -		
globID2	-0.063	-0.032	0.156	-0.186	- -	
globID3	-0.013	0.104	-0.032	0.636	-0.012	- -
globID4	0.024	-0.125	0.103	-0.093	0.175	-0.038

Expected Change for THETA-DELTA

	globID4
globID4	- -

Completely Standardized Expected Change for THETA-DELTA

	cet1	cet2	cet3	cet4	env1	env2
cet1	- -					
cet2	0.112	- -				
cet3	0.014	-0.010	- -			
cet4	-0.081	-0.090	0.034	- -		
env1	0.009	-0.042	-0.015	0.038	- -	
env2	-0.038	0.020	-0.015	0.054	-0.024	- -
env4	0.022	0.047	0.006	-0.070	0.051	-0.031
aut1	0.040	-0.033	0.021	-0.029	0.028	-0.007
aut2	0.005	-0.024	0.017	-0.010	-0.021	0.050

aut3	-0.046	0.014	-0.001	0.017	0.017	-0.037
aut4	0.019	-0.010	0.009	-0.004	-0.010	-0.029
qual1	-0.015	0.036	-0.005	-0.048	-0.028	0.023
qual2	-0.012	0.091	0.011	-0.053	-0.068	0.063
qual3	-0.027	0.017	0.036	0.029	0.007	0.080
qual4	-0.004	-0.032	0.033	-0.016	0.019	-0.030
qual5	0.021	-0.046	-0.063	0.054	-0.048	-0.003
qual6	0.028	-0.023	-0.023	0.039	0.064	-0.035
localID1	0.000	0.084	-0.035	-0.028	-0.019	-0.029
localID2	-0.012	0.038	0.047	-0.028	-0.007	-0.012
localID3	0.032	-0.095	-0.007	0.015	0.039	0.008
localID4	-0.042	-0.012	-0.019	0.089	0.017	0.012
tn_at	-0.057	-0.017	-0.049	0.159	-0.015	0.010
price1	-0.008	0.079	0.000	-0.045	-0.018	-0.028
price2	0.016	-0.006	-0.019	-0.012	-0.008	0.031
globatt1	-0.033	0.028	0.002	0.033	-0.040	0.012
globatt2	0.024	0.025	-0.030	-0.060	-0.036	0.021
globatt3	0.010	-0.072	0.030	0.019	0.036	0.019
globID1	0.018	0.036	-0.010	-0.050	0.025	0.019
globID2	-0.033	-0.013	0.011	0.040	-0.029	-0.013
globID3	0.004	-0.030	0.017	0.030	-0.007	-0.002
globID4	-0.037	-0.060	-0.004	0.038	-0.022	0.010

Completely Standardized Expected Change for THETA-DELTA

	env4	aut1	aut2	aut3	aut4	qual1
env4	- -					
aut1	-0.033	- -				
aut2	-0.008	-0.010	- -			
aut3	0.036	0.013	0.026	- -		
aut4	0.001	0.035	0.005	-0.071	- -	
qual1	0.021	0.048	-0.012	-0.054	0.024	- -
qual2	-0.043	0.031	0.005	-0.036	0.008	0.230
qual3	-0.028	-0.006	-0.011	-0.008	-0.026	-0.021

qual4	-0.001	-0.045	0.014	0.048	0.046	0.016
qual5	0.032	-0.014	0.007	0.022	-0.045	-0.034
qual6	-0.004	-0.025	-0.020	0.048	-0.026	-0.113
localID1	0.015	0.002	-0.062	-0.004	0.052	0.044
localID2	0.000	0.041	-0.001	-0.057	0.066	0.094
localID3	0.016	-0.025	-0.016	-0.003	0.033	-0.078
localID4	-0.038	-0.055	0.039	0.076	-0.041	-0.120
tn_at	0.006	-0.019	0.006	0.005	0.010	-0.042
price1	0.030	0.004	-0.006	0.013	0.031	-0.086
price2	-0.003	0.018	0.004	-0.013	-0.056	0.074
globatt1	0.014	-0.031	0.007	0.019	-0.019	-0.061
globatt2	-0.084	0.074	0.016	-0.051	0.042	0.132
globatt3	0.007	-0.006	-0.016	-0.001	0.009	-0.026
globID1	0.000	0.013	0.002	-0.025	-0.057	-0.027
globID2	0.082	0.024	0.010	0.017	0.031	-0.028
globID3	-0.059	-0.021	0.002	0.005	0.043	0.064
globID4	-0.012	-0.015	0.024	0.064	-0.030	-0.052

Completely Standardized Expected Change for THETA-DELTA

	qual2	qual3	qual4	qual5	qual6	localID1
qual2	- -					
qual3	-0.072	- -				
qual4	-0.002	-0.033	- -			
qual5	-0.022	-0.018	-0.025	- -		
qual6	-0.120	0.076	-0.015	0.143	- -	
localID1	0.015	-0.005	-0.038	0.005	0.013	- -
localID2	0.073	-0.025	-0.038	-0.064	-0.003	0.050
localID3	-0.047	0.065	0.047	0.011	-0.027	0.054
localID4	-0.041	-0.001	0.041	0.053	0.020	-0.018
tn_at	0.008	0.056	-0.019	0.038	-0.038	-0.093
price1	0.018	0.026	0.004	-0.015	0.027	0.050
price2	0.018	-0.016	-0.025	0.022	-0.048	-0.047
globatt1	-0.050	0.036	-0.018	0.074	-0.013	-0.005

globatt2	0.054	-0.073	-0.016	-0.049	-0.007	0.074
globatt3	-0.006	0.041	0.043	-0.059	0.030	-0.044
globID1	0.030	0.010	0.016	-0.028	-0.015	-0.013
globID2	-0.050	-0.009	0.028	0.005	0.049	-0.023
globID3	-0.009	-0.021	-0.053	0.030	-0.010	0.041
globID4	0.007	0.023	0.027	0.037	0.010	-0.062

Completely Standardized Expected Change for THETA-DELTA

	localID2	localID3	localID4	tn_at	price1	price2
localID2	- -					
localID3	-0.058	- -				
localID4	-0.075	0.036	- -			
tn_at	0.025	0.058	0.027	- -		
price1	-0.031	0.023	-0.016	0.039	- -	
price2	0.069	0.013	-0.059	-0.041	- -	- -
globatt1	-0.059	-0.055	0.098	0.004	-0.033	-0.024
globatt2	0.131	-0.055	-0.136	-0.022	0.042	0.046
globatt3	-0.029	0.071	0.019	0.009	0.028	-0.014
globID1	-0.010	-0.001	0.032	0.003	-0.002	0.051
globID2	-0.056	0.018	0.037	-0.010	0.071	-0.042
globID3	0.002	0.001	-0.079	-0.012	-0.022	-0.024
globID4	-0.048	0.035	0.168	0.042	-0.021	-0.057

Completely Standardized Expected Change for THETA-DELTA

	globatt1	globatt2	globatt3	globID1	globID2	globID3
globatt1	- -					
globatt2	0.063	- -				
globatt3	0.544	-0.239	- -			
globID1	0.005	0.021	-0.043	- -		
globID2	-0.031	-0.014	0.086	-0.083	- -	
globID3	-0.005	0.038	-0.015	0.235	-0.005	- -

globID4	0.013	-0.059	0.060	-0.044	0.100	-0.018

Completely Standardized Expected Change for THETA-DELTA

	globID4
globID4	- -

Maximum Modification Index is 52.20 for Element (13,12) of THETA-DELTA

!HV Daten aus Studie 1

Standardized Solution

LAMBDA-X

	ETHNO	GREEN	AUTHENTI	QUALITY	LOCALISM	PRICESEN
cet1	1.202	- -	- -	- -	- -	- -
cet2	1.163	- -	- -	- -	- -	- -
cet3	1.326	- -	- -	- -	- -	- -
cet4	1.098	- -	- -	- -	- -	- -
env1	- -	0.964	- -	- -	- -	- -
env2	- -	1.118	- -	- -	- -	- -
env4	- -	1.084	- -	- -	- -	- -
aut1	- -	- -	0.999	- -	- -	- -
aut2	- -	- -	0.943	- -	- -	- -
aut3	- -	- -	1.102	- -	- -	- -
aut4	- -	- -	1.023	- -	- -	- -
qual1	- -	- -	- -	0.978	- -	- -
qual2	- -	- -	- -	1.021	- -	- -
qual3	- -	- -	- -	0.856	- -	- -
qual4	- -	- -	- -	1.011	- -	- -
qual5	- -	- -	- -	1.043	- -	- -
qual6	- -	- -	- -	0.990	- -	- -

localID1	- -	- -	- -	- -	1.153	- -
localID2	- -	- -	- -	- -	1.056	- -
localID3	- -	- -	- -	- -	1.069	- -
localID4	- -	- -	- -	- -	0.952	- -
tn_at	- -	- -	- -	- -	- -	- -
price1	- -	- -	- -	- -	- -	1.255
price2	- -	- -	- -	- -	- -	1.443
globatt1	- -	- -	- -	- -	- -	- -
globatt2	- -	- -	- -	- -	- -	- -
globatt3	- -	- -	- -	- -	- -	- -
globID1	- -	- -	- -	- -	- -	- -
globID2	- -	- -	- -	- -	- -	- -
globID3	- -	- -	- -	- -	- -	- -
globID4	- -	- -	- -	- -	- -	- -

LAMBDA-X

	NUTZEN	GLOBAL	GID
cet1	- -	- -	- -
cet2	- -	- -	- -
cet3	- -	- -	- -
cet4	- -	- -	- -
env1	- -	- -	- -
env2	- -	- -	- -
env4	- -	- -	- -
aut1	- -	- -	- -
aut2	- -	- -	- -
aut3	- -	- -	- -
aut4	- -	- -	- -
qual1	- -	- -	- -
qual2	- -	- -	- -
qual3	- -	- -	- -
qual4	- -	- -	- -
qual5	- -	- -	- -

qual6	- -	- -	- -
localID1	- -	- -	- -
localID2	- -	- -	- -
localID3	- -	- -	- -
localID4	- -	- -	- -
tn_at	21.284	- -	- -
price1	- -	- -	- -
price2	- -	- -	- -
globatt1	- -	1.263	- -
globatt2	- -	1.022	- -
globatt3	- -	1.074	- -
globID1	- -	- -	1.524
globID2	- -	- -	0.836
globID3	- -	- -	1.390
globID4	- -	- -	0.678

PHI

	ETHNO	GREEN	AUTHENTI	QUALITY	LOCALISM	PRICESEN
ETHNO	1.000					
GREEN	0.652	1.000				
AUTHENTI	0.654	0.592	1.000			
QUALITY	0.655	0.525	0.738	1.000		
LOCALISM	0.526	0.416	0.654	0.590	1.000	
PRICESEN	-0.217	-0.255	-0.052	-0.006	0.143	1.000
NUTZEN	0.558	0.367	0.314	0.307	0.227	-0.425
GLOBAL	-0.029	0.089	0.044	0.019	0.123	0.253
GID	0.046	0.314	0.034	0.114	0.051	0.058

PHI

	NUTZEN	GLOBAL	GID
NUTZEN	1.000		

GLOBAL	-0.043	1.000	
GID	-0.033	0.195	1.000

!HV Daten aus Studie 1

Completely Standardized Solution

LAMBDA-X

	ETHNO	GREEN	AUTHENTI	QUALITY	LOCALISM	PRICESEN
cet1	0.844	- -	- -	- -	- -	- -
cet2	0.768	- -	- -	- -	- -	- -
cet3	0.854	- -	- -	- -	- -	- -
cet4	0.761	- -	- -	- -	- -	- -
env1	- -	0.823	- -	- -	- -	- -
env2	- -	0.831	- -	- -	- -	- -
env4	- -	0.746	- -	- -	- -	- -
aut1	- -	- -	0.848	- -	- -	- -
aut2	- -	- -	0.851	- -	- -	- -
aut3	- -	- -	0.884	- -	- -	- -
aut4	- -	- -	0.772	- -	- -	- -
qual1	- -	- -	- -	0.736	- -	- -
qual2	- -	- -	- -	0.784	- -	- -
qual3	- -	- -	- -	0.690	- -	- -
qual4	- -	- -	- -	0.793	- -	- -
qual5	- -	- -	- -	0.823	- -	- -
qual6	- -	- -	- -	0.778	- -	- -
localID1	- -	- -	- -	- -	0.815	- -
localID2	- -	- -	- -	- -	0.710	- -
localID3	- -	- -	- -	- -	0.772	- -
localID4	- -	- -	- -	- -	0.744	- -
tn_at	- -	- -	- -	- -	- -	- -
price1	- -	- -	- -	- -	- -	0.793
price2	- -	- -	- -	- -	- -	0.830

globatt1	- -	- -	- -	- -	- -	- -
globatt2	- -	- -	- -	- -	- -	- -
globatt3	- -	- -	- -	- -	- -	- -
globID1	- -	- -	- -	- -	- -	- -
globID2	- -	- -	- -	- -	- -	- -
globID3	- -	- -	- -	- -	- -	- -
globID4	- -	- -	- -	- -	- -	- -

LAMBDA-X

	NUTZEN	GLOBAL	GID
cet1	- -	- -	- -
cet2	- -	- -	- -
cet3	- -	- -	- -
cet4	- -	- -	- -
env1	- -	- -	- -
env2	- -	- -	- -
env4	- -	- -	- -
aut1	- -	- -	- -
aut2	- -	- -	- -
aut3	- -	- -	- -
aut4	- -	- -	- -
qual1	- -	- -	- -
qual2	- -	- -	- -
qual3	- -	- -	- -
qual4	- -	- -	- -
qual5	- -	- -	- -
qual6	- -	- -	- -
localID1	- -	- -	- -
localID2	- -	- -	- -
localID3	- -	- -	- -
localID4	- -	- -	- -
tn_at	1.000	- -	- -
price1	- -	- -	- -

price2	- -	- -	- -
globatt1	- -	0.860	- -
globatt2	- -	0.618	- -
globatt3	- -	0.806	- -
globID1	- -	- -	0.924
globID2	- -	- -	0.611
globID3	- -	- -	0.846
globID4	- -	- -	0.528

PHI

	ETHNO	GREEN	AUTHENTI	QUALITY	LOCALISM	PRICESEN
ETHNO	1.000					
GREEN	0.652	1.000				
AUTHENTI	0.654	0.592	1.000			
QUALITY	0.655	0.525	0.738	1.000		
LOCALISM	0.526	0.416	0.654	0.590	1.000	
PRICESEN	-0.217	-0.255	-0.052	-0.006	0.143	1.000
NUTZEN	0.558	0.367	0.314	0.307	0.227	-0.425
GLOBAL	-0.029	0.089	0.044	0.019	0.123	0.253
GID	0.046	0.314	0.034	0.114	0.051	0.058

PHI

	NUTZEN	GLOBAL	GID
NUTZEN	1.000		
GLOBAL	-0.043	1.000	
GID	-0.033	0.195	1.000

THETA-DELTA

cet1	cet2	cet3	cet4	env1	env2

```
        0.288      0.410      0.271      0.421      0.323      0.309

  THETA-DELTA

         env4       aut1       aut2       aut3       aut4      qual1
     --------   --------   --------   --------   --------   --------
        0.443      0.281      0.276      0.219      0.404      0.458

  THETA-DELTA

        qual2      qual3      qual4      qual5      qual6   localID1
     --------   --------   --------   --------   --------   --------
        0.385      0.524      0.372      0.322      0.395      0.336

  THETA-DELTA

     localID2   localID3   localID4      tn_at     price1     price2
     --------   --------   --------   --------   --------   --------
        0.496      0.404      0.446       - -       0.371      0.311

  THETA-DELTA

     globatt1   globatt2   globatt3    globID1    globID2    globID3
     --------   --------   --------   --------   --------   --------
        0.260      0.618      0.350      0.146      0.627      0.284

  THETA-DELTA

      globID4
     --------
        0.721

W_A_R_N_I_N_G: THETA-DELTA is not positive definite

                          Time used 0.859 seconds
```

3 Apples Structural Model (Summated Scores) for Hypothesis Evaluation

DATE: 8/19/2021
TIME: 12:44

L I S R E L 9.30 (64 Bit)

BY

Karl G. Jˆreskog & Dag Sˆrbom

This program is published exclusively by
Scientific Software International, Inc.
http://www.ssicentral.com

Copyright by Scientific Software International, Inc., 1981-2017
Use of this program is subject to the terms specified in the
Universal Copyright Convention.

!LEGEND for the expression used in this file:
PHB-Healthiness Bias summated factor scores, BIAS-Healthiness Bias construct
LocID-Local Identity summated factor scores, LOCALISM-Local Identity construct
CETsum-Consumer Ethnocentrism summated factor scores, ETHNO-Consumer Ethnocentrism construct
GloIDsum-Global Identity summated factor scores, GID-Global Identity construct
AUTH-Authenticity summated factor scores, AUTHENCI-Authenticity construct
GCVsum-Green Consumer Value summated factor scores, GREEN-Green Consumer Values construct
GloAttsum-Global Attitude summated factor scores, GLOBAL-Global Attitude construct
Pricesum-Price Consciousness/Sensibility summated factor scores, PRICESEN-Price Consciousness/Sensibility construct
tn_at-Part worth utility of the local choice, NUTZEN-part worth utility

The following lines were read from file C:\Users\Petra Riefler\Desktop\Aepfel_summatedscores.spl:

!Data

```
 !fpfel
 !Model 2, 16.08.2021 mit factor scores um Multikoll zu beheben
 Observed Variables: PHBsum LocIDsum CETsum GloIDsum AUTHsum GCVsum GloAttsum Pricesum tn_at age gender
edu urban HOUSESIZ food health
 Covariance Matrix:
 0.911
 -0.011 0.756
 0.051 0.086 0.758
 -0.068 -0.025 -0.014 0.791
 0.023 0.128 0.062 0.014 0.807
 0.060 0.006 -0.028 0.038 0.015 0.763
 0.033 -0.003 -0.055 0.034 0.066 0.003 0.745
 -0.043 -0.010 0.010 -0.008 0.024 0.003 0.014 0.746
 4.396 1.291 5.070 1.266 2.644 1.163 -1.519 -4.956 466.543
 0.867 1.933 1.839 -0.585 1.485 2.591 -0.918 -0.975 43.107 243.992
 0.002 -0.004 0.011 0.007 0.021 0.011 0.063 0.008 0.114 0.013 0.251
 -0.034 -0.157 -0.146 0.153 -0.208 0.184 -0.020 -0.069 -1.176 0.198 0.071 1.325
 0.062 0.070 0.011 0.058 -0.033 0.022 0.006 0.070 0.338 -0.311 0.025 -0.032 0.630
 -0.016 -0.061 -0.044 -0.047 -0.104 0.007 0.051 0.133 -1.423 -5.824 -0.001 0.257 0.175 1.182
 0.210 0.153 0.169 0.082 0.103 0.236 0.128 -0.194 5.040 3.992 0.038 0.174 -0.039 -0.188 1.596
 0.223 -0.100 0.093 0.016 0.194 0.535 -0.044 -0.211 7.466 2.917 0.113 0.259 -0.022 -0.049 0.551 2.328
 Sample Size: 251
 Latent Variables: ETHNO GREEN AUTHENTICITY BIAS LOCALISM PRICESENS NUTZEN GLOBAL GID SEX YEARS BILDUNG
LOCATION HCONSC
 Relationships:
 CETsum=1*ETHNO
 set the error variance of CETsum equal to 0.121
 GCVsum = 1*GREEN
 set the error variance of GCVsum equal to 0.163
 AUTHsum = 1*AUTHENTICITY
 set the error variance of AUTHsum equal to 0.098
 LocIDsum = 1*LOCALISM
 set the error variance of LocIDsum equal to 0.158
 Pricesum=1*PRICESENS
 set the error variance of Pricesum equal to 0.208
```

```
PHBsum = 1*BIAS
set the error variance of PHBsum equal to 0.105
GloAttsum = 1*GLOBAL
set the error variance of GloAttsum equal to 0.210
GloIDsum=1*GID
set the error variance of GloIDsum equal to 0.178
tn_at=1*NUTZEN
set the error variance of tn_at equal to 0
NUTZEN = ETHNO GREEN AUTHENTICITY BIAS LOCALISM PRICESENS GLOBAL GID
Options: ND=3 SC RS MI
Path Diagram
End of Problem

Sample Size =   251

!HV Daten aus Studie 1
```

Covariance Matrix

	tn_at	PHBsum	LocIDsum	CETsum	GloIDsum	AUTHsum
tn_at	466.543					
PHBsum	4.396	0.911				
LocIDsum	1.291	-0.011	0.756			
CETsum	5.070	0.051	0.086	0.758		
GloIDsum	1.266	-0.068	-0.025	-0.014	0.791	
AUTHsum	2.644	0.023	0.128	0.062	0.014	0.807
GCVsum	1.163	0.060	0.006	-0.028	0.038	0.015
GloAttsu	-1.519	0.033	-0.003	-0.055	0.034	0.066
Pricesum	-4.956	-0.043	-0.010	0.010	-0.008	0.024

Covariance Matrix

	GCVsum	GloAttsu	Pricesum

```
  GCVsum      0.763
GloAttsu      0.003      0.745
Pricesum      0.003      0.014      0.746

Total Variance = 472.820 Generalized Variance = 47.642

Largest Eigenvalue = 466.722 Smallest Eigenvalue = 0.588

Condition Number = 28.182


!HV Daten aus Studie 1

Number of Iterations = 0

LISREL Estimates (Maximum Likelihood)

        Measurement Equations


   tn_at = 1.000*NUTZEN,, R≤ = 1.000


  PHBsum = 1.000*BIAS, Errorvar.= 0.105, R≤ = 0.885

LocIDsum = 1.000*LOCALISM, Errorvar.= 0.158, R≤ = 0.791

  CETsum = 1.000*ETHNO, Errorvar.= 0.121, R≤ = 0.840

GloIDsum = 1.000*GID, Errorvar.= 0.178, R≤ = 0.775

 AUTHsum = 1.000*AUTHENTI, Errorvar.= 0.0980, R≤ = 0.879

  GCVsum = 1.000*GREEN, Errorvar.= 0.163, R≤ = 0.786
```

```
 GloAttsu = 1.000*GLOBAL, Errorvar.= 0.210, R≤ = 0.718

 Pricesum = 1.000*PRICESEN, Errorvar.= 0.208, R≤ = 0.721


         Structural Equations


   NUTZEN = 7.241*ETHNO + 1.618*GREEN + 3.344*AUTHENTI + 4.638*BIAS + 0.416*LOCALISM - 9.016*PRICESEN -
2.731*GLOBAL
 Standerr  (1.727)       (1.802)       (1.617)          (1.485)      (1.852)           (1.989)
(2.019)
 Z-values   4.193         0.898         2.068            3.122        0.225            -4.533
-1.353
 P-values   0.000         0.369         0.039            0.002        0.822             0.000
0.176

             + 2.619*GID, Errorvar.= 346.038, R≤ = 0.258
     Standerr (1.804)               (33.553)
     Z-values  1.452                 10.313
     P-values  0.147                 0.000


         Covariance Matrix of Independent Variables

                ETHNO      GREEN   AUTHENTI       BIAS   LOCALISM   PRICESEN
             --------   --------   --------   --------   --------   --------
    ETHNO       0.637
              (0.068)
                9.414

    GREEN      -0.028      0.600
              (0.048)    (0.068)
               -0.583      8.809
```

AUTHENTI	0.062	0.015	0.709			
	(0.050)	(0.050)	(0.072)			
	1.252	0.303	9.842			
BIAS	0.051	0.060	0.023	0.806		
	(0.053)	(0.053)	(0.054)	(0.081)		
	0.971	1.137	0.425	9.911		
LOCALISM	0.086	0.006	0.128	-0.011	0.598	
	(0.048)	(0.048)	(0.050)	(0.052)	(0.067)	
	1.788	0.125	2.562	-0.210	8.861	
PRICESEN	0.010	0.003	0.024	-0.043	-0.010	0.538
	(0.047)	(0.048)	(0.049)	(0.052)	(0.047)	(0.067)
	0.211	0.063	0.490	-0.825	-0.211	8.079
GLOBAL	-0.055	0.003	0.066	0.033	-0.003	0.014
	(0.048)	(0.048)	(0.049)	(0.052)	(0.047)	(0.047)
	-1.156	0.063	1.344	0.634	-0.063	0.297
GID	-0.014	0.038	0.014	-0.068	-0.025	-0.008
	(0.049)	(0.049)	(0.050)	(0.054)	(0.049)	(0.048)
	-0.286	0.774	0.278	-1.265	-0.512	-0.165

Covariance Matrix of Independent Variables

	GLOBAL	GID
GLOBAL	0.535	
	(0.067)	
	8.045	
GID	0.034	0.613

(0.049)	(0.071)
0.701	8.682

Covariance Matrix of Latent Variables

	NUTZEN	ETHNO	GREEN	AUTHENTI	BIAS	LOCALISM
NUTZEN	466.543					
ETHNO	5.070	0.637				
GREEN	1.163	-0.028	0.600			
AUTHENTI	2.644	0.062	0.015	0.709		
BIAS	4.396	0.051	0.060	0.023	0.806	
LOCALISM	1.291	0.086	0.006	0.128	-0.011	0.598
PRICESEN	-4.956	0.010	0.003	0.024	-0.043	-0.010
GLOBAL	-1.519	-0.055	0.003	0.066	0.033	-0.003
GID	1.266	-0.014	0.038	0.014	-0.068	-0.025

Covariance Matrix of Latent Variables

	PRICESEN	GLOBAL	GID
PRICESEN	0.538		
GLOBAL	0.014	0.535	
GID	-0.008	0.034	0.613

Log-likelihood Values

	Estimated Model	Saturated Model
Number of free parameters(t)	45	45
-2ln(L)	3228.790	3228.790
AIC (Akaike, 1974)*	3318.790	3318.790
BIC (Schwarz, 1978)*	3477.436	3477.436

```
*LISREL uses AIC= 2t - 2ln(L) and BIC = tln(N)- 2ln(L)


                          Goodness-of-Fit Statistics

 Degrees of Freedom for (C1)-(C2)                     0
 Browne's (1984) ADF Chi-Square (C2_NT)               0.0 (P = 1.0000)

                 The Model is Saturated, the Fit is Perfect !


 !HV Daten aus Studie 1

 Modification Indices and Expected Change

 No Non-Zero Modification Indices for LAMBDA-Y

 No Non-Zero Modification Indices for LAMBDA-X

 No Non-Zero Modification Indices for BETA

 No Non-Zero Modification Indices for GAMMA

 No Non-Zero Modification Indices for PHI

 No Non-Zero Modification Indices for PSI

 No Non-Zero Modification Indices for THETA-EPS

 !HV Daten aus Studie 1

 Standardized Solution

         LAMBDA-Y
```

	NUTZEN
tn_at	21.600

LAMBDA-X

	ETHNO	GREEN	AUTHENTI	BIAS	LOCALISM	PRICESEN
PHBsum	- -	- -	- -	0.898	- -	- -
LocIDsum	- -	- -	- -	- -	0.773	- -
CETsum	0.798	- -	- -	- -	- -	- -
GloIDsum	- -	- -	- -	- -	- -	- -
AUTHsum	- -	- -	0.842	- -	- -	- -
GCVsum	- -	0.775	- -	- -	- -	- -
GloAttsu	- -	- -	- -	- -	- -	- -
Pricesum	- -	- -	- -	- -	- -	0.733

LAMBDA-X

	GLOBAL	GID
PHBsum	- -	- -
LocIDsum	- -	- -
CETsum	- -	- -
GloIDsum	- -	0.783
AUTHsum	- -	- -
GCVsum	- -	- -
GloAttsu	0.731	- -
Pricesum	- -	- -

GAMMA

	ETHNO	GREEN	AUTHENTI	BIAS	LOCALISM	PRICESEN
NUTZEN	0.268	0.058	0.130	0.193	0.015	-0.306

GAMMA

	GLOBAL	GID
NUTZEN	-0.092	0.095

Correlation Matrix of ETA and KSI

	NUTZEN	ETHNO	GREEN	AUTHENTI	BIAS	LOCALISM
NUTZEN	1.000					
ETHNO	0.294	1.000				
GREEN	0.070	-0.045	1.000			
AUTHENTI	0.145	0.092	0.023	1.000		
BIAS	0.227	0.071	0.086	0.030	1.000	
LOCALISM	0.077	0.139	0.010	0.197	-0.016	1.000
PRICESEN	-0.313	0.017	0.005	0.039	-0.065	-0.018
GLOBAL	-0.096	-0.094	0.005	0.107	0.050	-0.005
GID	0.075	-0.022	0.063	0.021	-0.097	-0.041

Correlation Matrix of ETA and KSI

	PRICESEN	GLOBAL	GID
PRICESEN	1.000		
GLOBAL	0.026	1.000	
GID	-0.014	0.059	1.000

PSI

NUTZEN
0.742

Regression Matrix ETA on KSI (Standardized)

	ETHNO	GREEN	AUTHENTI	BIAS	LOCALISM	PRICESEN
NUTZEN	0.268	0.058	0.130	0.193	0.015	-0.306

Regression Matrix ETA on KSI (Standardized)

	GLOBAL	GID
NUTZEN	-0.092	0.095

!HV Daten aus Studie 1

Completely Standardized Solution

LAMBDA-Y

	NUTZEN
tn_at	1.000

LAMBDA-X

	ETHNO	GREEN	AUTHENTI	BIAS	LOCALISM	PRICESEN
PHBsum	- -	- -	- -	0.941	- -	- -
LocIDsum	- -	- -	- -	- -	0.889	- -
CETsum	0.917	- -	- -	- -	- -	- -
GloIDsum	- -	- -	- -	- -	- -	- -
AUTHsum	- -	- -	0.937	- -	- -	- -
GCVsum	- -	0.887	- -	- -	- -	- -
GloAttsu	- -	- -	- -	- -	- -	- -
Pricesum	- -	- -	- -	- -	- -	0.849

LAMBDA-X

	GLOBAL	GID
PHBsum	- -	- -
LocIDsum	- -	- -
CETsum	- -	- -
GloIDsum	- -	0.880
AUTHsum	- -	- -
GCVsum	- -	- -
GloAttsu	0.847	- -
Pricesum	- -	- -

GAMMA

	ETHNO	GREEN	AUTHENTI	BIAS	LOCALISM	PRICESEN
NUTZEN	0.268	0.058	0.130	0.193	0.015	-0.306

GAMMA

	GLOBAL	GID
NUTZEN	-0.092	0.095

Correlation Matrix of ETA and KSI

	NUTZEN	ETHNO	GREEN	AUTHENTI	BIAS	LOCALISM
NUTZEN	1.000					
ETHNO	0.294	1.000				
GREEN	0.070	-0.045	1.000			
AUTHENTI	0.145	0.092	0.023	1.000		
BIAS	0.227	0.071	0.086	0.030	1.000	
LOCALISM	0.077	0.139	0.010	0.197	-0.016	1.000

PRICESEN	-0.313	0.017	0.005	0.039	-0.065	-0.018
GLOBAL	-0.096	-0.094	0.005	0.107	0.050	-0.005
GID	0.075	-0.022	0.063	0.021	-0.097	-0.041

Correlation Matrix of ETA and KSI

	PRICESEN	GLOBAL	GID
PRICESEN	1.000		
GLOBAL	0.026	1.000	
GID	-0.014	0.059	1.000

PSI

NUTZEN
0.742

W_A_R_N_I_N_G: THETA-EPS is not positive definite

THETA-DELTA

PHBsum	LocIDsum	CETsum	GloIDsum	AUTHsum	GCVsum
0.115	0.209	0.160	0.225	0.121	0.214

THETA-DELTA

GloAttsu	Pricesum
0.282	0.279

Regression Matrix ETA on KSI (Standardized)

ETHNO	GREEN	AUTHENTI	BIAS	LOCALISM	PRICESEN

NUTZEN	0.268	0.058	0.130	0.193	0.015	-0.306

Regression Matrix ETA on KSI (Standardized)

	GLOBAL	GID
NUTZEN	-0.092	0.095

Time used 0.016 seconds

3 Tomatoes Structural Model (Summated Scores) for Hypothesis Evaluation

DATE: 8/19/2021
TIME: 12:45

L I S R E L 9.30 (64 Bit)

BY

Karl G. Jˆreskog & Dag Sˆrbom

This program is published exclusively by
Scientific Software International, Inc.
http://www.ssicentral.com

Copyright by Scientific Software International, Inc., 1981-2017
Use of this program is subject to the terms specified in the
Universal Copyright Convention.

!LEGEND for the expression used in this file:
PHB-Healthiness Bias summated factor scores, BIAS-Healthiness Bias construct
LocID-Local Identity summated factor scores, LOCALISM-Local Identity construct
CETsum-Consumer Ethnocentrism summated factor scores, ETHNO-Consumer Ethnocentrism construct
GloIDsum-Global Identity summated factor scores, GID-Global Identity construct
AUTH-Authenticity summated factor scores, AUTHENCI-Authenticity construct
GCVsum-Green Consumer Value summated factor scores, GREEN-Green Consumer Values construct
GloAttsum-Global Attitude summated factor scores, GLOBAL-Global Attitude construct
Pricesum-Price Consciousness/Sensibility summated factor scores, PRICESEN-Price Consciousness/Sensibility construct
tn_at-Part worth utility of the local choice, NUTZEN-part worth utility

The following lines were read from file C:\Users\Petra Riefler\Desktop\Tomaten_summatedscores.spl:

```
!Data
!Tomaten
!Model 2, 16.08.2021 mit factor scores um Multikoll zu beheben
Observed Variables: PHBsum LocIDsum CETsum GloIDsum AUTHsum GCVsum GloAttsum Pricesum tn_at age gender
edu urban HOUSESIZ food health
Covariance Matrix:
0.800
0.067 0.818
0.054 0.003 0.790
0.071 0.010 0.006 0.955
0.085 0.016 -0.003 -0.035 0.739
0.000 0.028 0.135 0.054 0.051 0.736
-0.034 0.031 0.012 0.015 -0.046 0.020 0.903
0.053 0.063 -0.059 0.026 -0.022 -0.092 0.044 0.812
3.471 2.536 7.973 -0.528 1.691 4.084 -0.356 -7.458 453.020
0.462 0.244 0.617 -2.879 2.337 0.516 -2.909 -1.246 35.130 265.632
0.040 -0.008 0.088 -0.007 -0.004 0.063 0.071 0.040 0.839 -1.257 0.250
-0.134 -0.028 -0.112 0.170 -0.115 0.108 0.052 -0.151 -0.188 -0.959 0.003 1.359
0.019 0.078 -0.033 -0.091 -0.004 -0.029 -0.049 0.041 0.280 -0.482 0.020 -0.117 0.672
0.074 0.070 -0.027 0.169 -0.039 -0.104 0.073 0.058 -1.273 -6.878 0.016 0.063 0.126 1.315
0.123 0.079 0.198 0.131 0.088 0.353 0.090 -0.168 4.828 2.449 0.025 0.082 -0.094 -0.162 1.403
0.160 0.113 0.160 0.039 0.133 0.433 -0.077 -0.139 4.961 3.952 0.120 0.251 -0.013 -0.070 0.570 2.295
Sample Size: 253
Latent Variables: ETHNO GREEN AUTHENTICITY BIAS LOCALISM PRICESENS NUTZEN GLOBAL GID SEX YEARS BILDUNG
LOCATION HCONSC
Relationships:
CETsum=1*ETHNO
set the error variance of CETsum equal to 0.121
GCVsum = 1*GREEN
set the error variance of GCVsum equal to 0.163
AUTHsum = 1*AUTHENTICITY
set the error variance of AUTHsum equal to 0.098
LocIDsum = 1*LOCALISM
set the error variance of LocIDsum equal to 0.158
Pricesum=1*PRICESENS
```

```
set the error variance of Pricesum equal to 0.208
GloAttsum = 1*GLOBAL
set the error variance of GloAttsum equal to 0.210
GloIDsum=1*GID
set the error variance of GloIDsum equal to 0.178
PHBsum = 1*BIAS
set the error variance of PHBsum equal to 0.105
tn_at=1*NUTZEN
set the error variance of tn_at equal to 0
NUTZEN = ETHNO GREEN AUTHENTICITY BIAS LOCALISM PRICESENS GLOBAL GID
Options: ND=3 SC RS MI
Path Diagram
End of Problem

Sample Size =   253

!HV Daten aus Studie 1
```

Covariance Matrix

	tn_at	PHBsum	LocIDsum	CETsum	GloIDsum	AUTHsum
tn_at	453.020					
PHBsum	3.471	0.800				
LocIDsum	2.536	0.067	0.818			
CETsum	7.973	0.054	0.003	0.790		
GloIDsum	-0.528	0.071	0.010	0.006	0.955	
AUTHsum	1.691	0.085	0.016	-0.003	-0.035	0.739
GCVsum	4.084	- -	0.028	0.135	0.054	0.051
GloAttsu	-0.356	-0.034	0.031	0.012	0.015	-0.046
Pricesum	-7.458	0.053	0.063	-0.059	0.026	-0.022

Covariance Matrix

	GCVsum	GloAttsu	Pricesum

```
           --------   --------   --------
  GCVsum      0.736
GloAttsu      0.020      0.903
Pricesum     -0.092      0.044      0.812

Total Variance = 459.573 Generalized Variance = 49.559

Largest Eigenvalue = 453.368 Smallest Eigenvalue = 0.520

Condition Number = 29.520



!HV Daten aus Studie 1

Number of Iterations = 0

LISREL Estimates (Maximum Likelihood)

        Measurement Equations


   tn_at = 1.000*NUTZEN,, R≤ = 1.000


  PHBsum = 1.000*BIAS, Errorvar.= 0.105, R≤ = 0.869

LocIDsum = 1.000*LOCALISM, Errorvar.= 0.158, R≤ = 0.807

  CETsum = 1.000*ETHNO, Errorvar.= 0.121, R≤ = 0.847

GloIDsum = 1.000*GID, Errorvar.= 0.178, R≤ = 0.814

 AUTHsum = 1.000*AUTHENTI, Errorvar.= 0.0980, R≤ = 0.867
```

```
  GCVsum = 1.000*GREEN, Errorvar.= 0.163, R≤ = 0.779

GloAttsu = 1.000*GLOBAL, Errorvar.= 0.210, R≤ = 0.767

Pricesum = 1.000*PRICESEN, Errorvar.= 0.208, R≤ = 0.744


        Structural Equations


  NUTZEN = 9.964*ETHNO + 2.654*GREEN + 1.303*AUTHENTI + 4.646*BIAS + 4.311*LOCALISM - 11.747*PRICESEN +
0.126*GLOBAL
 Standerr  (1.495)       (1.716)       (1.477)          (1.436)      (1.502)           (1.690)
(1.492)
 Z-values   6.666         1.547         0.882            3.236        2.871            -6.952
0.0842
 P-values   0.000         0.122         0.378            0.001        0.004             0.000
0.933

            - 0.972*GID, Errorvar.= 245.392, R≤ = 0.458
    Standerr (1.375)              (26.033)
    Z-values -0.707                9.426
    P-values  0.480                0.000


        Covariance Matrix of Independent Variables

               ETHNO      GREEN   AUTHENTI       BIAS   LOCALISM   PRICESEN
            --------   --------   --------   --------   --------   --------
   ETHNO       0.669
             (0.070)
               9.525

   GREEN       0.135      0.573
             (0.049)    (0.065)
```

	2.773	8.756				
AUTHENTI	-0.003	0.051	0.641			
	(0.048)	(0.046)	(0.066)			
	-0.062	1.097	9.756			
BIAS	0.054	- -	0.085	0.695		
	(0.050)	(0.048)	(0.049)	(0.071)		
	1.078	0.000	1.748	9.771		
LOCALISM	0.003	0.028	0.016	0.067	0.660	
	(0.051)	(0.049)	(0.049)	(0.051)	(0.073)	
	0.059	0.574	0.327	1.313	9.075	
PRICESEN	-0.059	-0.092	-0.022	0.053	0.063	0.604
	(0.050)	(0.049)	(0.049)	(0.051)	(0.051)	(0.072)
	-1.169	-1.880	-0.452	1.044	1.226	8.366
GLOBAL	0.012	0.020	-0.046	-0.034	0.031	0.044
	(0.053)	(0.051)	(0.051)	(0.053)	(0.054)	(0.054)
	0.226	0.390	-0.894	-0.636	0.573	0.816
GID	0.006	0.054	-0.035	0.071	0.010	0.026
	(0.055)	(0.053)	(0.053)	(0.055)	(0.056)	(0.055)
	0.110	1.022	-0.662	1.288	0.180	0.469

Covariance Matrix of Independent Variables

	GLOBAL	GID
GLOBAL	0.693	
	(0.080)	
	8.632	

GID	0.015	0.777
	(0.058)	(0.085)
	0.257	9.151

Covariance Matrix of Latent Variables

	NUTZEN	ETHNO	GREEN	AUTHENTI	BIAS	LOCALISM
NUTZEN	453.020					
ETHNO	7.973	0.669				
GREEN	4.084	0.135	0.573			
AUTHENTI	1.691	-0.003	0.051	0.641		
BIAS	3.471	0.054	- -	0.085	0.695	
LOCALISM	2.536	0.003	0.028	0.016	0.067	0.660
PRICESEN	-7.458	-0.059	-0.092	-0.022	0.053	0.063
GLOBAL	-0.356	0.012	0.020	-0.046	-0.034	0.031
GID	-0.528	0.006	0.054	-0.035	0.071	0.010

Covariance Matrix of Latent Variables

	PRICESEN	GLOBAL	GID
PRICESEN	0.604		
GLOBAL	0.044	0.693	
GID	0.026	0.015	0.777

Log-likelihood Values

	Estimated Model	Saturated Model
Number of free parameters(t)	45	45
-2ln(L)	3264.502	3264.502
AIC (Akaike, 1974)*	3354.502	3354.502
BIC (Schwarz, 1978)*	3513.505	3513.505

```
*LISREL uses AIC= 2t - 2ln(L) and BIC = tln(N)- 2ln(L)


                          Goodness-of-Fit Statistics

 Degrees of Freedom for (C1)-(C2)                        0
 Browne's (1984) ADF Chi-Square (C2_NT)                  0.0 (P = 1.0000)

                 The Model is Saturated, the Fit is Perfect !


 !HV Daten aus Studie 1

 Modification Indices and Expected Change

 No Non-Zero Modification Indices for LAMBDA-Y

 No Non-Zero Modification Indices for LAMBDA-X

 No Non-Zero Modification Indices for BETA

 No Non-Zero Modification Indices for GAMMA

 No Non-Zero Modification Indices for PHI

 No Non-Zero Modification Indices for PSI

 No Non-Zero Modification Indices for THETA-EPS

 !HV Daten aus Studie 1

 Standardized Solution

         LAMBDA-Y
```

	NUTZEN
tn_at	21.284

LAMBDA-X

	ETHNO	GREEN	AUTHENTI	BIAS	LOCALISM	PRICESEN
PHBsum	- -	- -	- -	0.834	- -	- -
LocIDsum	- -	- -	- -	- -	0.812	- -
CETsum	0.818	- -	- -	- -	- -	- -
GloIDsum	- -	- -	- -	- -	- -	- -
AUTHsum	- -	- -	0.801	- -	- -	- -
GCVsum	- -	0.757	- -	- -	- -	- -
GloAttsu	- -	- -	- -	- -	- -	- -
Pricesum	- -	- -	- -	- -	- -	0.777

LAMBDA-X

	GLOBAL	GID
PHBsum	- -	- -
LocIDsum	- -	- -
CETsum	- -	- -
GloIDsum	- -	0.881
AUTHsum	- -	- -
GCVsum	- -	- -
GloAttsu	0.832	- -
Pricesum	- -	- -

GAMMA

ETHNO	GREEN	AUTHENTI	BIAS	LOCALISM	PRICESEN

NUTZEN	0.383	0.094	0.049	0.182	0.165	-0.429

GAMMA

	GLOBAL	GID
NUTZEN	0.005	-0.040

Correlation Matrix of ETA and KSI

	NUTZEN	ETHNO	GREEN	AUTHENTI	BIAS	LOCALISM
NUTZEN	1.000					
ETHNO	0.458	1.000				
GREEN	0.253	0.218	1.000			
AUTHENTI	0.099	-0.005	0.084	1.000		
BIAS	0.196	0.079	- -	0.127	1.000	
LOCALISM	0.147	0.005	0.046	0.025	0.099	1.000
PRICESEN	-0.451	-0.093	-0.156	-0.035	0.082	0.100
GLOBAL	-0.020	0.018	0.032	-0.069	-0.049	0.046
GID	-0.028	0.008	0.081	-0.050	0.097	0.014

Correlation Matrix of ETA and KSI

	PRICESEN	GLOBAL	GID
PRICESEN	1.000		
GLOBAL	0.068	1.000	
GID	0.038	0.020	1.000

PSI

NUTZEN
0.542

Regression Matrix ETA on KSI (Standardized)

	ETHNO	GREEN	AUTHENTI	BIAS	LOCALISM	PRICESEN
NUTZEN	0.383	0.094	0.049	0.182	0.165	-0.429

Regression Matrix ETA on KSI (Standardized)

	GLOBAL	GID
NUTZEN	0.005	-0.040

!HV Daten aus Studie 1

Completely Standardized Solution

LAMBDA-Y

	NUTZEN
tn_at	1.000

LAMBDA-X

	ETHNO	GREEN	AUTHENTI	BIAS	LOCALISM	PRICESEN
PHBsum	- -	- -	- -	0.932	- -	- -
LocIDsum	- -	- -	- -	- -	0.898	- -
CETsum	0.920	- -	- -	- -	- -	- -
GloIDsum	- -	- -	- -	- -	- -	- -
AUTHsum	- -	- -	0.931	- -	- -	- -
GCVsum	- -	0.882	- -	- -	- -	- -
GloAttsu	- -	- -	- -	- -	- -	- -
Pricesum	- -	- -	- -	- -	- -	0.862

LAMBDA-X

	GLOBAL	GID
PHBsum	- -	- -
LocIDsum	- -	- -
CETsum	- -	- -
GloIDsum	- -	0.902
AUTHsum	- -	- -
GCVsum	- -	- -
GloAttsu	0.876	- -
Pricesum	- -	- -

GAMMA

	ETHNO	GREEN	AUTHENTI	BIAS	LOCALISM	PRICESEN
NUTZEN	0.383	0.094	0.049	0.182	0.165	-0.429

GAMMA

	GLOBAL	GID
NUTZEN	0.005	-0.040

Correlation Matrix of ETA and KSI

	NUTZEN	ETHNO	GREEN	AUTHENTI	BIAS	LOCALISM
NUTZEN	1.000					
ETHNO	0.458	1.000				
GREEN	0.253	0.218	1.000			
AUTHENTI	0.099	-0.005	0.084	1.000		
BIAS	0.196	0.079	- -	0.127	1.000	

LOCALISM	0.147	0.005	0.046	0.025	0.099	1.000
PRICESEN	-0.451	-0.093	-0.156	-0.035	0.082	0.100
GLOBAL	-0.020	0.018	0.032	-0.069	-0.049	0.046
GID	-0.028	0.008	0.081	-0.050	0.097	0.014

Correlation Matrix of ETA and KSI

	PRICESEN	GLOBAL	GID
PRICESEN	1.000		
GLOBAL	0.068	1.000	
GID	0.038	0.020	1.000

PSI

NUTZEN
0.542

W_A_R_N_I_N_G: THETA-EPS is not positive definite

THETA-DELTA

PHBsum	LocIDsum	CETsum	GloIDsum	AUTHsum	GCVsum
0.131	0.193	0.153	0.186	0.133	0.221

THETA-DELTA

GloAttsu	Pricesum
0.233	0.256

Regression Matrix ETA on KSI (Standardized)

	ETHNO	GREEN	AUTHENTI	BIAS	LOCALISM	PRICESEN
NUTZEN	0.383	0.094	0.049	0.182	0.165	-0.429

Regression Matrix ETA on KSI (Standardized)

	GLOBAL	GID
NUTZEN	0.005	-0.040

Time used 0.000 seconds
